# Systematic evaluation with practical guidelines for single-cell and spatially resolved transcriptomics data simulation under multiple scenarios

**DOI:** 10.1186/s13059-024-03290-y

**Published:** 2024-06-03

**Authors:** Hongrui Duo, Yinghong Li, Yang Lan, Jingxin Tao, Qingxia Yang, Yingxue Xiao, Jing Sun, Lei Li, Xiner Nie, Xiaoxi Zhang, Guizhao Liang, Mingwei Liu, Youjin Hao, Bo Li

**Affiliations:** 1 College of Life Sciences, Chongqing Normal University, Chongqing, 401331 People’s Republic of China; 2 Chongqing Key Laboratory of Big Data for Bio Intelligence, Chongqing University of Posts and Telecommunications, Chongqing, 400065 People’s Republic of China; 3 Institute of Pathology and Southwest Cancer Center, Southwest Hospital, Army Medical University, Chongqing, 400038 People’s Republic of China; 4 Zhejiang Provincial Key Laboratory of Precision Diagnosis and Therapy for Major Gynecological Diseases, Women’s Hospital, Zhejiang University School of Medicine, Hangzhou, 310058 People’s Republic of China; 5 Key Laboratory of Biorheological Science and Technology, Ministry of Education, Bioengineering College, Chongqing University, Chongqing, 400044 People’s Republic of China; 6 Key Laboratory of Clinical Laboratory Diagnostics, College of Laboratory Medicine, Chongqing Medical University, Chongqing, 400016 People’s Republic of China

**Keywords:** Evaluation, Single-cell transcriptomics, Spatially resolved transcriptomics, Data simulation, Guideline

## Abstract

**Background:**

Single-cell RNA sequencing (scRNA-seq) and spatially resolved transcriptomics (SRT) have led to groundbreaking advancements in life sciences. To develop bioinformatics tools for scRNA-seq and SRT data and perform unbiased benchmarks, data simulation has been widely adopted by providing explicit ground truth and generating customized datasets. However, the performance of simulation methods under multiple scenarios has not been comprehensively assessed, making it challenging to choose suitable methods without practical guidelines.

**Results:**

We systematically evaluated 49 simulation methods developed for scRNA-seq and/or SRT data in terms of accuracy, functionality, scalability, and usability using 152 reference datasets derived from 24 platforms. SRTsim, scDesign3, ZINB-WaVE, and scDesign2 have the best accuracy performance across various platforms. Unexpectedly, some methods tailored to scRNA-seq data have potential compatibility for simulating SRT data. Lun, SPARSim, and scDesign3-tree outperform other methods under corresponding simulation scenarios. Phenopath, Lun, Simple, and MFA yield high scalability scores but they cannot generate realistic simulated data. Users should consider the trade-offs between method accuracy and scalability (or functionality) when making decisions. Additionally, execution errors are mainly caused by failed parameter estimations and appearance of missing or infinite values in calculations. We provide practical guidelines for method selection, a standard pipeline Simpipe (https://github.com/duohongrui/simpipe; 10.5281/zenodo.11178409), and an online tool Simsite (https://www.ciblab.net/software/simshiny/) for data simulation.

**Conclusions:**

No method performs best on all criteria, thus a good-yet-not-the-best method is recommended if it solves problems effectively and reasonably. Our comprehensive work provides crucial insights for developers on modeling gene expression data and fosters the simulation process for users.

**Supplementary Information:**

The online version contains supplementary material available at 10.1186/s13059-024-03290-y.

## Background

Rapid advancements in scRNA-seq and SRT technologies provide unprecedented opportunities to investigate gene expression at the cellular and spatial levels, thereby unraveling the cellular heterogeneity and underlying molecular mechanisms of biochemical processes [1–5]. The widespread adoption of these technologies has generated a huge amount of scRNA-seq and SRT data, which has fueled the emergence of computational bioinformatics tools [6]. In this context, researchers have implemented evaluations of the algorithms used in each analytical step to assist users in selecting the most suitable methods [7–19]. Besides the utilization of real data produced by experiments, simulated data have been extensively used as an important reference in benchmark studies [7–9, 12–19], since the well-built data simulation strategy can not only provide explicit ground truth, but also allow users to generate specialized datasets for particular cases by adjusting parameters according to their needs.

In recent years, a plethora of tools have been developed to simulate scRNA-seq data [6] and methods tailored to simulate SRT data have emerged in 2023 [20–22]. Most simulation methods were developed mainly based on statistical distributions, in which a reference dataset was used to estimate important parameters. The new gene expression values were then drawn from the sample space using those estimated parameters [23–26]. Those parametric methods with distributional assumptions are flexible and enable to handle simulation tasks under various scenarios, but the assumptions may not always hold true due to the biological and technical variability in the data [27]. Alternatively, a deep learning-based method was proposed to generate in silico scRNA-seq data [28], utilizing generative adversarial networks (GAN) to avoid pre-defined assumptions. To enhance the interpretability of these methods, researchers have shifted their focus toward simulating scRNA-seq data based on the principle of gene expression regulation [29] and RNA velocity theory [30]. Apart from the gene expression matrices, numerous methods can also be applied to four simulation scenarios for generating different types of ground truth, including disparate cell groups (or spatial domains), differentially expressed genes (DEGs), cell batches, and trajectories. This versatility enables researchers to assess the performance of analytical methods across a spectrum of simulated biological contexts and enhance the robustness of experimental designs.

Given the diversities of underlying models implemented in the simulation methods and various applicable scenarios, it is necessary to comprehensively evaluate their performance. Efforts have been made toward the benchmarking of simulation methods for scRNA-seq data [31, 32], but they solely focused on partial simulation scenarios where methods could be applied, without fully assessing the fidelity and reliability of the simulated ground truth by multiple metrics from different viewpoints. Thus, this incomplete evaluation limits the broad adoption of methods with varied functionalities. For example, Cao et al. [31] only assessed the ability of methods to simulate genes with different expression patterns, and the study by Crowell et al. [32] merely considered the method functionalities of generating different cell batches and clusters. In particular, the simulation methods for SRT data have not been systematically evaluated by previous benchmark studies, and their abilities to generate realistic data and their performances under specific simulation scenarios (e.g., the simulation of spatial domains) are still unclear. Additionally, it is not beneficial for users to choose and use appropriate methods in a user-friendly way, without offering an explicit guideline for method selection and a standard pipeline from data simulation to result assessment. For some simulation methods, execution errors frequently arise during the process of parameter estimation and data simulation, but the underlying reasons have not been summarized and analyzed. As a result, it is not conducive to improving current methods and advising users to adopt the methods with low error rates. In conclusion, addressing these limitations is imperative, as gaining a comprehensive understanding of the pros and cons of existing methods will empower users to make informed decisions. Moreover, it will assist developers in refining algorithms and provide new insights into the characteristics of scRNA-seq and SRT data.

Here, we conducted a comprehensive assessment of 49 simulation methods for scRNA-seq and/or SRT data using 152 real reference datasets to evaluate their accuracy, functionality, scalability, and usability. Different from previous studies, we adopted various metrics and proposed several novel approaches to fully quantify the reliability of the simulated datasets and the functionality performance of methods under four simulation scenarios. Besides measuring the required time and occupied computational resources, we explored the time and memory complexity with respect to the cell or gene numbers for each simulation method. Additionally, we gathered up the error messages during the study and analyzed the underlying reasons for those execution failures. By elaborately summarizing the evaluation results, we provided an explicit guideline of method selection for users and a standard pipeline called Simpipe to achieve the process from data simulation to result assessment. Moreover, users can also access our interactive website (https://www.ciblab.net/software/simshiny/) to select suitable methods and simulate datasets. Our evaluation provides valuable insights for the development of new simulation algorithms and highlights the promising future of data simulation strategies for other prominent research fields, such as multi-omics and multi-modality.

## Results

### The overall benchmark framework

**Fig. 1:**
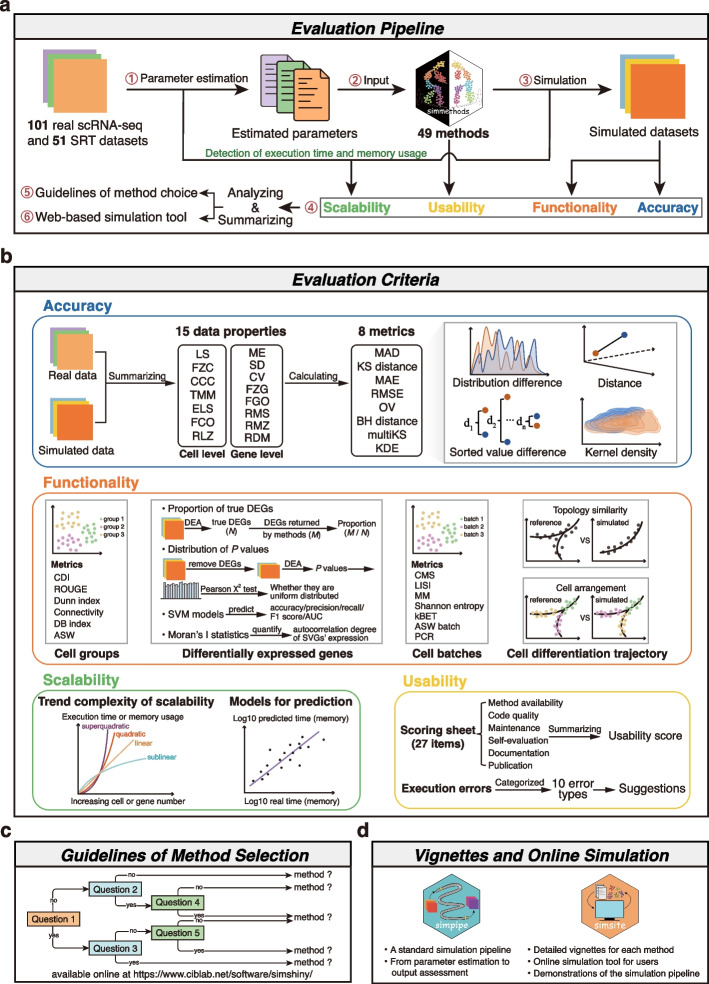
Overall framework of the benchmark study. **a** The evaluation pipeline. Key estimated parameters were learned from 152 reference datasets and then used to simulate new datasets through 49 simulation methods. Next, the method performance in terms of accuracy, functionality, scalability, and usability was evaluated. Based on the benchmark results, a guideline for method selection and an online simulation tool were provided for users. **b** Four evaluation criteria. The adopted criteria include accuracy, functionality, scalability, and usability. Method accuracy was assessed through 8 metrics based on 15 data properties. Functionality was quantified using different metrics under four simulation scenarios. Scalability evaluation was performed to uncover the trend complexity with varying cell or gene numbers and the scalability datasets were modeled for prediction. Usability was evaluated through 27 scoring items across six aspects and the causes of execution errors were summarized. **c** The guideline of method selection and suggestions for users and developers. **d** The standard simulation pipeline Simpipe with corresponding vignettes and online simulation tool. DEA, differential expression analysis; SVGs, spatially variable genes

**Fig. 2:**
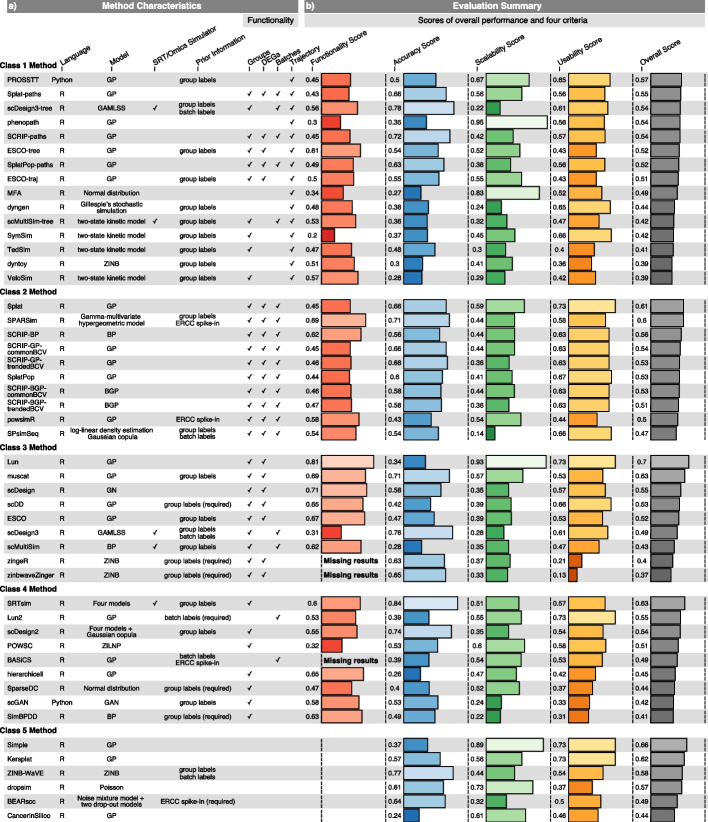
Characteristics of methods and evaluation results based on the four criteria. **a** Method characteristics. The programming language, model, ability to simulate SRT or omics data, required prior information and functionality of 49 methods are listed. All methods were categorized into 5 classes according to the type and number of functionalities they possess (see “ Methods”). They were ranked by the overall scores in each class. **b** Scores of four criteria and overall performance of methods. Due to the absence of simulated cell group labels in zingeR and zinbwaveZinger, results of the functionality of simulating cell groups and DEGs were not available. Notably, BASiCS frequently suffered from execution errors on many datasets; thereby, the functionality performance of simulating cell batches was missing. GP, gamma-Poisson; BGP, beta-gamma-Poisson; BP, beta-Poisson; ZINB, zero-inflated negative binomial; ZILNP, zero-inflated log-normal Poisson; GAN, generative adversarial network; GN, gamma-Normal; GAMLSS, generalized additive model for location, scale and shape; Four models, which means that these methods determine an appropriate distribution to model gene expression from four candidate distributions, including Poisson, zero-inflated Poisson (ZIP), negative binomial (NB, i.e., GP), and ZINB distribution

A systematic and comprehensive evaluation of 49 simulation methods was performed to assess their accuracy, functionality, scalability, and usability, by using 101 scRNA-seq and 51 SRT datasets generated by 24 platforms, such as Smart-seq2 and Stereo-seq (Fig. 1a, Additional file 1: Fig. S1 and Additional file 2: Table S1-3). To clarify the simulation scenarios that current methods can be applied to, we summarized four essential aspects of functionality, including the abilities to simulate cell groups (or spatial domains), DEGs, cell batches, and cell differentiation trajectories (Fig. 1b). All methods were categorized into 5 classes, according to the type and number of simulation scenarios they can be employed (Fig. 2a, Additional file 2: Table S1 and Methods). Moreover, we unified the parameter names across different functionalities and methods to simplify user-defined settings (Additional file 1: Supplementary Note Sect. 1, Additional file 2: Table S2).

Except for the methods lacking functionality (assessed by three criteria), the remains were assessed using four criteria (Fig. 1b, Methods): (1) Accuracy was assessed by 8 metrics based on 15 data properties on the cell or gene level, reflecting the ability to generate “realistic” simulated data. (2) Functionality evaluation was an unbiased quantification of method performance in four simulation scenarios with different metrics. (3) Scalability evaluation was performed to monitor the occupied computational resources and unveil the relationship between execution time or memory usage and the number of cells or genes. (4) Usability assessment was conducted by manually scoring the terms of the checklist which was summarized from nine authoritative references [33–41]. Usability covered six aspects: method availability, code quality, self-evaluation, maintenance, documentation, and publication (Additional file 2: Table S4).

Guidelines and recommendations for users to select the suitable method were provided based on our benchmark results. We also encapsulated 49 methods within the Simmethods package with comprehensive vignettes (https://github.com/duohongrui/simmethods). Furthermore, we established a standard pipeline called Simpipe (https://github.com/duohongrui/simpipe), along with an online data simulation tool Simsite (https://www.ciblab.net/software/simshiny/), to streamline simulation tasks and evaluate their outputs (Fig. 1c, d).

### Accuracy performance of methods over various metrics and data properties

We observed that SRTsim, a specialized simulator for SRT data, achieved the highest accuracy score (0.84), followed by scDesign3-tree (0.78), ZINB-WaVE (0.77), scDesign3 (0.76), and scDesign2 (0.74) (Fig. 2a, b). It is noted that ZINB-WaVE has also been validated to generate more realistic data by other studies [31, 32]. In contrast, CancerInSilico, hierarchicell, MFA, scMultiSim, VeloSim, and dyntoy cannot generate realistic data due to their poor performance in accuracy assessment. Although each class had at least one method ranking within the top 10 for accuracy, Class2 methods performed better and ranked higher than other classes in general, with a mean accuracy score of 0.60.

**Fig. 3:**
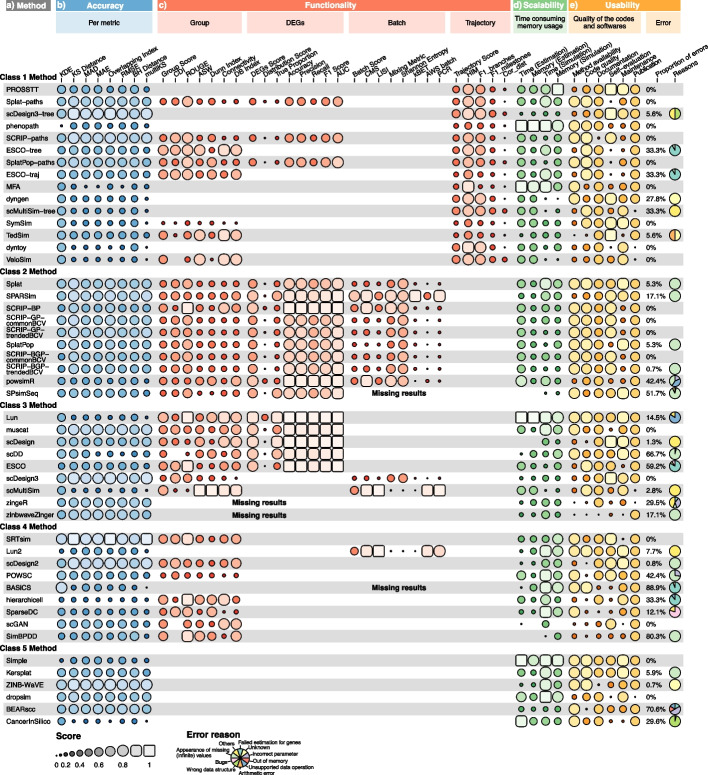
Detailed results of the four evaluation criteria. **a** Methods used for the benchmark study. They are ranked according to their overall score within each class (see Fig. 2). **b** The accuracy scores of each metric. **c** Metric scores for each simulation functionality. SPsimSeq produced errors on the reference datasets with batch effects, which led to the missing results on the functionality performance of simulating cell batches. The reasons for the missing results in zingeR, zinbwaveZinger, and BASiCS have been illustrated in Fig. 2. **d** Scalability scores for execution time and memory usage in parameter estimation or data simulation step. **e** Usability scores in six aspects and the information of execution errors for each method

SRTsim, ZINB-WaVE, scDesign3-tree, scDesign3, SPARSim, muscat, and scDesign2 showed superior performance on all accuracy metrics (Fig. 3a, b), while some methods such as phenopath, Lun2, and hierarchicell typically yielded low scores. Additionally, for Class2 methods, the scores of most metrics (e.g., KS: 0.68 $\pm$ 0.11, MAD: 0.59 $\pm$ 0.06, and OV: 0.64 $\pm$ 0.12) were higher and more robust than those of other classes (Additional file 1: Fig. S2a), suggesting that they possess the powerful ability to simulate data which resembled the real data.

Regarding the scores given to each data property, some methods constantly achieved good performance on the characterization of different properties from real data (e.g., SRTsim, scDesign3, and ZINB-WaVE), while some of the others (e.g., hierarchicell, MFA, scMultiSim, and CancerInSilico) demonstrated poorly to simulate data that resembled the real one (Additional file 1: Fig. S3). We also found that some data properties, such as the cell–cell correlation (CCC) and fraction of cell outliers (FCO), can only be captured well by certain methods. For instance, the FCO property was well characterized by SPARsim (score = 0.89) and SRTsim (score = 0.86). SRTsim also showed an obviously good performance in CCC (score = 0.90). Remarkably, the methods showing significant differences in accuracy scores between gene-level and cell-level properties performed excellently in the former but faltered in the latter (Additional file 1: Fig. S4). That is probably because current methods mainly focus on the modeling of various gene-level properties, such as mean and standard deviation of gene expression, gene-wise dropout, and gene–gene correlations, but only a few of cell-level properties (e.g., cell library size and cell-wise dropout) were characterized from real data. For the Class2 methods, they still showed appreciable performance across many data properties (Additional file 1: Fig. S2b), such as library size per cell (LS: 0.70 ± 0.11), effective library size per cell (ELS: 0.69 ± 0.11), and fraction of zeros per cell (FZC: 0.60 ± 0.10), as their scores were more robust and higher than those of other classes.

### Accuracy performance of methods on different platforms and techniques

**Fig. 4:**
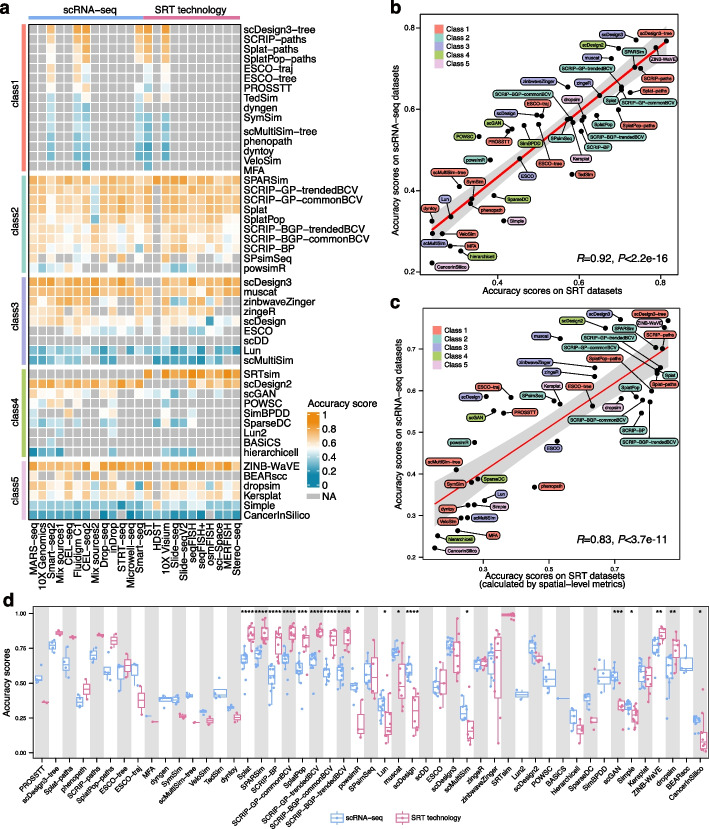
Accuracy performance of methods on different platforms and techniques. **a** Heatmap of accuracy scores on different platforms. Methods are grouped by each class and ranked according to their accuracy scores. “Mix sources1” indicates the data derived from two experimental platforms (Smart-seq2 and 10 × Genomics). “Mix sources2” indicates the mixture data derived from platforms of CEL-seq and CEL-seq2. **b** The correlation between the accuracy scores on SRT datasets and scRNA-seq datasets (Pearson’s* r* = 0.92). The 95% confidence interval is shown in gray. **c** The “spatial-level” metrics (spatial-KDE and spatial-multiKS) were adopted to recalculate the accuracy scores for the simulated SRT datasets (see “ Methods”). The correlation analysis was performed between the “spatial-level” scores on SRT datasets and scores on scRNA-seq datasets (Pearson’s* r* = 0.83). **d** Boxplots of accuracy scores on the datasets generated by scRNA-seq or SRT technology for each method. Each dot represents one method applied on a platform. The two-sided Wilcoxon test was performed on the accuracy scores derived from scRNA-seq and SRT datasets. *, *P* < 0.05; **, *P* < 0.01; ***, *P* < 0.001; ****, *P* < 0.0001

Except for Mix sources2 (Pearson’s *r* = 0.58, *P* < 0.01), strong correlations were observed between the scores of different platforms and overall accuracy (Additional file 1: Fig. S5). It suggests that the methods with excellent performance in accuracy (e.g., SRTsim, scDesign2, scDesign3, and ZINB-WaVE) can also be used across a wide range of platforms (Fig. 4a and Additional file 1: Fig. S3b). Additionally, the significant differences in accuracy performance between the read-based and UMI-based platforms were observed in many methods (Additional file 1: Fig. S6), showing their distinct preferences for the count quantification strategy of a given data.

The accuracy scores obtained from SRT and scRNA-seq techniques were highly correlated (Pearson’s* r* = 0.92) (Fig. 4b). By employing “spatial-level” metrics (“spatial-KDE” and “spatial-multiKS,” see “ Methods”) to specifically quantify the similarity between simulated and real SRT datasets, we still observed a high correlation of method accuracy performance (Pearson’s* r* = 0.83) on two types of reference data (Fig. 4c). It was revealed that well-performed simulation methods tailored to scRNA-seq data can also generate reliable SRT datasets without obvious conflicts. Unexpectedly, some methods like Splat, SPARSim, SCRIP (containing all five different variants), SplatPop, dropsim, and ZINB-WaVE performed significantly better on SRT data than scRNA-seq data (*P* < 0.01), highlighting their strong compatibility in simulating SRT data (Fig. 4d). In contrast, powsimR (*P* < 0.05), Lun (*P* < 0.05), muscat (*P* < 0.05), scDesign (*P* < 0.0001), and scGAN (*P* < 0.001) were more adept at simulating scRNA-seq data (Fig. 4d).

### Accuracy performance of different models

**Fig. 5:**
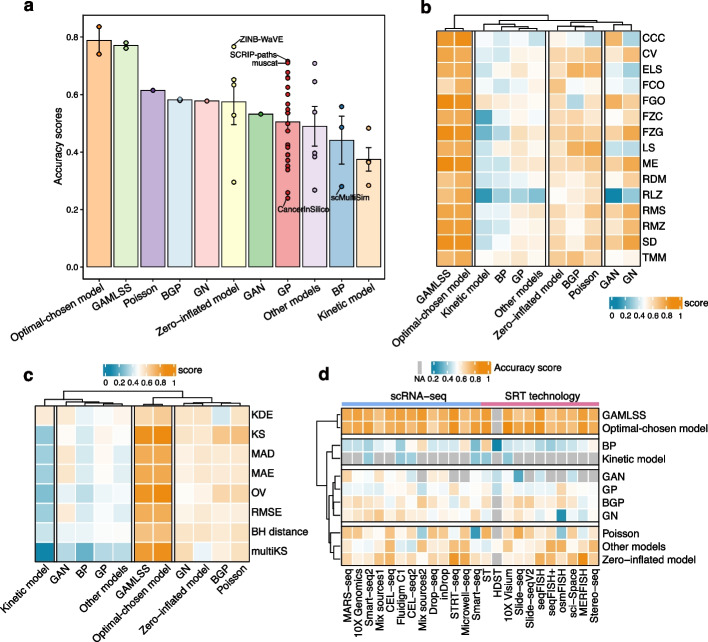
Accuracy performance of different models. **a** The bar plot of the accuracy scores of different models. Each dot represents a simulation method belonging to the model group. The error bar represents the mean ± s.e. of the accuracy scores in each model. **b–d** The heatmap showing the scores of different models across data properties, metrics, and transcriptomics technologies. The optimal-chosen model is defined as the algorithm used to determine the best-fit statistical distribution for each gene when modeling. All models are clustered into 4 groups using the *k*-means algorithm

To uncover the accuracy performance of methods based on different distributional assumptions or algorithms, we summarized the scores across methods with the same underlying models (Fig. 5a). In general, the methods with the optimal-chosen model or generalized additive model for location, scale, and shape (GAMLSS) had superior performance in accuracy, while the methods using kinetic model and beta-Poisson (BP) distribution demonstrated poorly and yielded low accuracy scores. Additionally, we observed the considerably varied performance of methods within the same model group. ZINB-WaVE, built on the zero-inflated negative binomial (ZINB) distribution, had the highest accuracy score in the model group. SCRIP-paths and muscat performed best among the methods employing Gamma-Poisson (GP) distribution. However, CancerInSilico and scMultiSim had the lowest ranking of accuracy within their categories. It was indicated that besides the choice of prior distributional assumption, the accuracy performance of methods may also be influenced by other crucial factors, such as the ability of methods to characterize diverse gene expression patterns and account for the data variability.

Based on the scores of different metrics and data properties, the models were clustered into four clusters. The optimal-chosen model and GAMLSS consistently showed the powerful ability to characterize different data properties (Fig. 5b) from real data and better performance under various metrics (Fig. 5c). The models based on zero-inflated, Poisson, and Beta-Gamma-Poisson (BGP) distributions were clustered together, as they yielded mediocre scores of metrics and data properties in general. We also observed that the original Poisson distribution model consistently outperformed other variants (e.g., BP and GP) across all properties and metrics (Fig. 5b, c), thus resulting in a higher overall accuracy score than other variant models (Fig. 5a). Furthermore, we found the property of CCC cannot be well captured by most models (Fig. 5b). The relation of library size and zero proportion of cells (RLZ) was also hard to be characterized, especially for the GAN and kinetic model. That was because they obtained low scores both on the library size (LS) and the fraction of zeros per cell (FZC).

The optimal-chosen model and GAMLSS demonstrated excellent simulation outcomes across real datasets generated by various platforms, whereas the BP distribution merely performed well on a few platforms (Fig. 5d). Compared with the models based on GP, BGP, Gamma-Normal (GN) distributions and GAN, Poisson, and zero-inflated distribution models showed better performance on most scRNA-seq and SRT platforms in general. Notably, the Poisson distribution model yielded a particularly low accuracy score on Smart-seq.

### Method performance on different functionalities

**Fig. 6:**
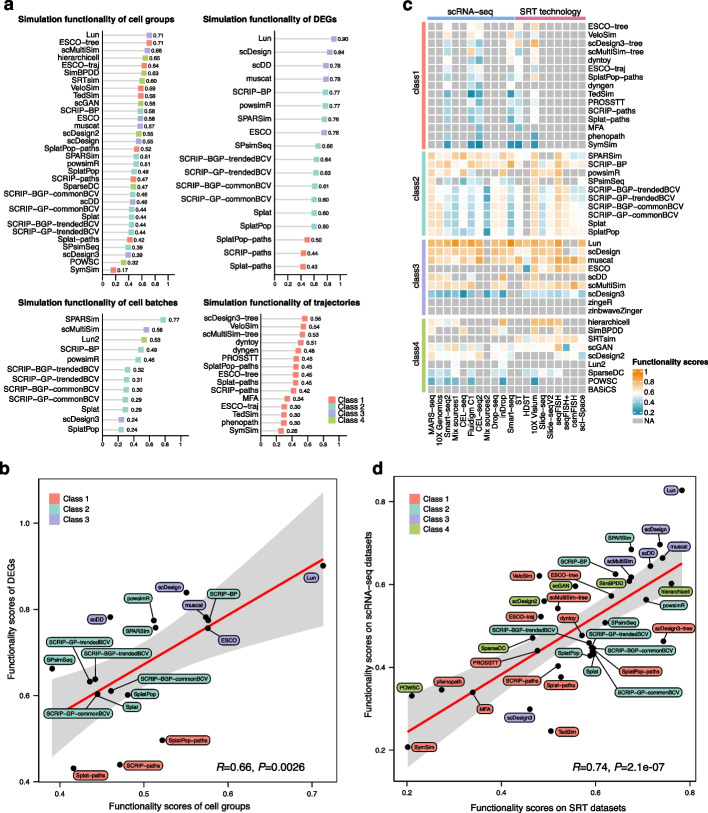
Functionality performance of methods. **a** Lollipop plots of the ranked methods in each simulation functionality. Method classes are marked by different colors. **b** The correlation between the functionality scores of simulated cell groups and DEGs (Pearson’s* r* = 0.66). The 95% confidence interval is shown in gray. **c** Functionality scores of methods applied to the reference data generated by different platforms. The methods are grouped by each class and ranked according to their functionality scores. **d** The correlation between the functionality scores on SRT datasets and scRNA-seq datasets (Pearson’s* r* = 0.74)

To assess the performance of the methods in each functionality, rankings were determined based on their respective functionality scores (Fig. 6a). Lun was the best method for simulating datasets with specified cell groups (score = 0.71) and DEGs (score = 0.90). Among methods with simulation functionality of cell batches, SPARSim achieved relatively high scores in all metrics (Additional file 1: Fig. S7) and showed the most remarkable performance (score = 0.77), followed by scMultiSim (0.56) and Lun2 (0.53) (Fig. 6a). Class1 methods performed poorly on F1_milestones_ and Cor_dist_ metrics (Additional file 1: Fig. S7), resulting in low scores in trajectory evaluation. This suggested that current methods cannot accurately simulate the cells that should be assigned to the correct milestones or positions within a trajectory.

We conducted the correlation analysis to explore the relationship between the method performance of different functionalities. The results revealed that the functionality scores of cell groups were correlated with those of DEGs (Pearson’s *r* = 0.66) (Fig. 6b). Additionally, the functionality performance of simulating cell groups and batches (Pearson’s *r* = 0.65), as well as DEGs and cell batches (Pearson’s *r* = 0.81), also exhibited the positive consistency (Additional file 1: Fig. S8a,c), but the correlation was relatively low between the functionality scores of cell groups and trajectories (Pearson’s *r* = 0.38, Additional file 1: Fig. S8b).

Moreover, we noticed that a few methods, such as SPARSim and muscat, consistently demonstrated favorable performance in both accuracy and functionality. However, several methods, including Lun, hierarchicell, and scDesign3, exhibited considerable differences between their accuracy and functionality performance (Fig. 2). Generally, the correlation between the scores of the accuracy and functionality assessment was not statistically significant (Pearson’s *r* = -0.01, *P* = 0.95), which also indicated the disagreement of method performance on the two evaluation criteria (Additional file 1: Fig. S9).

Specifically, we adopted Moran’s *I* statistics to quantify the degree of autocorrelation for the simulated spatially variable genes (SVGs) in mimicked SRT data. The results showed that Moran’s *I* statistics were close to 0 (Additional file 1: Fig. S10), indicating that there is no relationship between the gene expression levels within adjacent spots. Consequently, the simulation methods can hardly generate SRT data with clear expression patterns of SVGs in space. This limitation may arise from the incapacity of scRNA-seq data simulators to additionally integrate spatial coordinates and effectively capture the gene expression patterns across spatial domains from reference SRT data.

### Functionality performance on datasets generated by diverse platforms and techniques

We further investigated the effect of reference datasets generated by different platforms on the functionality performance. Many methods in Class3 such as Lun, scDesign, muscat, and scMultiSim performed well on all platforms (Fig. 6c), and the functionality scores were generally higher than those of other classes (Additional file 1: Fig. S11a). However, the performance of scDesign3, SymSim, and POWSC was completely opposite (Fig. 6c), resulting in low functionality scores (Fig. 2b). When it came to simulating cell groups, Class3 methods still had the outstanding ability on many platforms. While Class2 methods performed well in accuracy, they showed the inferior ability to simulate cell groups (Additional file 1: Fig. S11b).

There was a high correlation (Pearson’s *r* = 0.74, *P* < 0.001) between the functionality scores of the SRT and the scRNA-seq data (Fig. 6d). This correlation also held true when evaluating the functionality of groups (Pearson’s *r* = 0.82, *P* < 0.001) (Additional file 1: Fig. S12a) or DEGs (Pearson’s *r* = 0.80, *P* < 0.001) (Additional file 1: Fig. S12b), showing the consistent performance of method functionalities between datasets generated by both scRNA-seq and SRT techniques. For the group simulation functionality, the majority of the top performing methods (e.g., Lun, ESCO-tree, scMultiSim, ESCO-traj, VeloSim, and scGAN) (Fig. 6a) obtained higher scores on scRNA-seq data than SRT data (Additional file 1: Fig. S13a). Conversely, for the functionalities related to DEGs and trajectories, most simulation methods tailored to scRNA-seq data exhibited better performance on SRT data (Additional file 1: Fig. S13b,c).

### Scalability evaluation of simulation methods

For users, running time and consumed computational resources are essential for the practical applications of methods. By constructing the generalized linear model (see “ Methods”), we analyzed the effects of cell or gene numbers on running time and memory consumption. Our findings revealed that most methods exhibited either linear or sublinear time (or memory) complexity with respect to the cell or gene numbers (Additional file 1: Fig. S14-21). However, dyntoy had the quadratic time complexity in the estimation step and quadratic memory complexity in the simulation step as the cell or gene numbers increased. We also observed that the memory consumption of SRTsim tended to increase super-quadratically with the cell numbers when it was modeling gene expression data, while scDesign exhibited this trend as the gene numbers increased in the data simulation step. We illustrated the reasons for the negative slopes of the fitted lines for some methods and other special situations in Additional file 1: Supplementary Discussion.

To predict the time and memory consumption required by each simulation method, we modeled the data of execution time and memory usage using random forest (RF) and the shape constrained additive model (SCAM) [42] (“ Methods”). Subsequently, the actual time (memory) and the predicted time (memory) were compared by calculating the Pearson correlation coefficients between them, which revealed a high degree of correlation (Additional file 1: Fig. S22-23).

In the parameter estimation step, scGAN, BASiCS, scDD, scDesign2, scDesign3, TedSim, Lun2, and ZINB-WaVE exhibited longer execution time compared with other methods, while some methods such as scDesign2, scDesign3, scDesign3-tree, zinbwaveZinger, SparseDC, ZINB-WaVE, and BEARscc consumed more memory resources (Fig. 3a, d). In particular, we found that scDesign2, scDesign3, and ZINB-WaVE showed powerful capability of generating more realistic data in accuracy assessment (Fig. 2) at the expense of more time and memory consumption for modeling the gene expression data. In the simulation step, SimBPDD, SPsimSeq, scMultiSim-tree, scMultiSim, dyngen and VeloSim demonstrated lower time scalability scores. Additionally, dyngen and Velosim also required significantly more memory, indicating that they may not be suitable for simulating large datasets (Fig. 3a, d). Overall, some methods were capable of completing simulation tasks within reasonable time and memory constraints, such as phenopath, Splat, Simple, Lun, PROSSTT, and dropsim, allowing users to make a trade-off between accuracy and scalability in certain situations.

### Usability and execution errors

To quantify the usability of each method, scoring rules were established in terms of method availability, code quality, self-evaluation, documentation, and publication (Additional file 2: Table S4). We found that not all methods met the basic principles. For instance, zinbwaveZinger lacks usage instructions and illustrative examples, and an official software license, thus it has the lowest usability score (Additional file 1: Fig. S24a,b). Although no method achieves flawless performance across all six aspects (Fig. 3e), some methods such as Kersplat (score = 0.73), Lun (score = 0.73), Lun2 (score = 0.73), Simple (score = 0.73), Splat (score = 0.73), SplatPop (score = 0.67), SymSim (score = 0.66), SPsimSeq (score = 0.66), and scDD (score = 0.66) demonstrated excellent usability (Fig. 2a, b and Additional file 1: Fig. S24a). Therefore, they can be served as paradigms for software development, evaluation, and maintenance.

We further summarized 10 categories of execution errors (Fig. 3e, Additional file 2: Table S5). Approximately 61% of methods encountered errors, and the error proportions varied greatly across methods, ranging from 0.7% (SCRIP-BGP-trendedBCV and ZINB-WaVE) to 88.9% (BASiCS) (Fig. 3a, e). The error type called “appearance of missing (infinite) values” accounted for a substantial part in many methods. This can be attributed to unexpected missing values in vectors or matrices, resulting in errors when they are utilized in conditional statements or mathematical calculations. Certain methods, such as BASiCS, hierarchicell, and ESCO, frequently encountered failures in parameter estimation. It was the error termed “failed estimation for genes” that generally occurred in the situation where the expression values of some genes may not conform with the distribution or the gene-level properties cannot be successfully fitted by the model implemented in these methods. Drawing upon the main causes, we provided developers with practical recommendations to avoid common errors (Additional file 2: Table S6).

### Practical guidelines for method selection

**Fig. 7:**
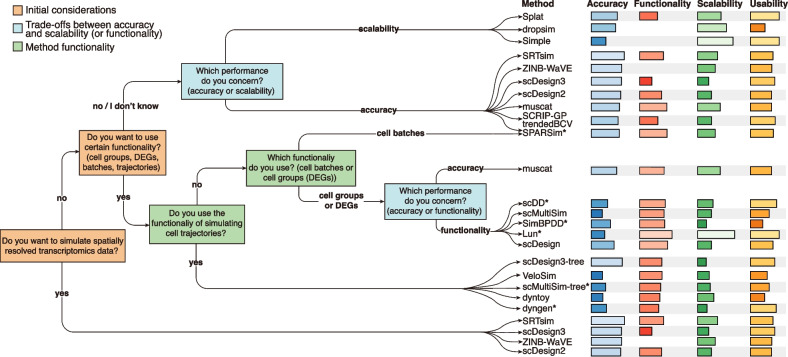
Practical guidelines for selecting simulation methods. When selecting a simulation method, users should consider two critical questions: whether or not to simulate SRT data and whether or not to use the method functionality under specific simulation scenarios. In some cases, users should trade-off the accuracy against scalability (or functionality) depending on the preferred aspect of method performance. Additionally, some methods, such as SPARSim, scDD, and SimBPDD have more than 10% error rates in our study, which are marked by an asterisk. Barplots of scores on the four evaluation criteria are shown next to the guideline

Based on the benchmark results, we provided a practical guideline to assist users in selecting appropriate simulation methods (Fig. 7). Users should consider whether to simulate SRT data and use the method functionality under specific simulation scenarios. As no method excels across all evaluation criteria, users are supposed to consider the trade-offs between accuracy and scalability, as well as accuracy and functionality. In some cases, a good-yet-not-the-best method is recommended as long as it provides a feasible solution. To facilitate this process, we have developed an online website that guides users step by step in selecting the suitable simulation method based on their requirements (https://www.ciblab.net/software/simshiny/).

## Discussion

We comprehensively evaluated 49 simulation methods designed for scRNA-seq and/or SRT data in terms of accuracy, functionality, scalability, and usability with practical guidelines for method selection. Generally, the accuracy performance of most simulation methods may be influenced by three key factors: (1) the choice of prior distributional assumption, (2) the diversity of gene expression patterns, and (3) the ability to account for data variability (i.e., technical and biological variation). Most simulation methods typically assume that the gene expression in scRNA-seq data follows a specific distribution (e.g., Poisson, ZIP, NB, and ZINB). However, there is no consensus on the distributional assumption adopted for modeling gene expression in scRNA-seq data. For example, some studies argued that NB is sufficient for modeling UMI-based scRNA-seq data [24, 43, 44], while others advocated the Poisson distribution is a better fit [45, 46]. For plate-based scRNA-seq data, there is still a controversy regarding whether zero-inflated models should be used [24, 43, 47]. Given these disagreements, developers are advised to initially model the data using several candidate statistical distributions and subsequently adopt the appropriate distributional assumption to better accommodate diverse gene expression data [48]. It has been proven that the expression levels of all genes within a scRNA-seq dataset do not follow the same statistical distribution [24, 47, 48], reflecting the diversity of gene expression patterns. Consequently, modeling gene expression data using a single parametric distribution may lead to the loss of important information about data properties. Additionally, confounding technical and biological effects can prevent the simulation method from accurately capturing the heterogeneity in the scRNA-seq data, which may affect the reliability and fidelity of simulated data. Effectively disentangling these variance effects is pivotal in modeling gene expression data, which is conducive to explicitly identifying gene expression patterns, improving the goodness-of-fit of the model and generating more realistic simulated data. In brief, to improve the accuracy and flexibility of simulation methods, developers should comprehensively consider and account for diverse gene expression patterns and data variability based on the reasonable choice of a distribution assumption.

According to the results of the accuracy assessment, we found the performance of different models varied considerably (Fig. 5a). Based on the optimal-chosen model, SRTsim and scDesign2 exhibit superior accuracy performance (Fig. 2). Specifically, they judged and selected the most suitable distribution for each gene from four candidate distributions (Poisson, ZIP, NB, and ZINB), avoiding the limitation of modeling gene expression data only using a single parametric distribution. This strategy can effectively characterize diverse gene expression patterns in real data, improve the goodness-of-fit, and can be applied across a broad spectrum of gene expression data. Furthermore, scDesign3, a multi-omics data simulator derived from GAMLSS, has two outstanding advantages in effectively characterizing data properties and gene expression heterogeneity from real-world data. On the one hand, scDesign3 allows the introduction of smooth functions and various covariates (e.g., experimental conditions, cell types, pseudotime, and spatial coordinates) to model mean gene expression, dispersion, and the proportion of zeros, which can be flexibly adapted to the data with different cell states and experimental designs. On the other hand, scDesign3 and scDesign2 model the joint distribution of multiple genes based on the marginal distributions of individual genes. By preserving the structure of gene correlations, both methods can precisely delineate intricate relationships across different levels of gene expression in real datasets, thus the expression patterns will be further consolidated in simulated data. While scGAN, a deep learning-based method, can discern different gene expression patterns, its performance on accuracy and functionality was found to be mediocre (Figs. 2 and Fig. 6a). This may stem from its sensitivity to the variance effects because generating high-quality cells usually requires training scGAN on the library-size normalized data, as mentioned in the original paper [28]. The methods relying on the two-state kinetic model, such as VeloSim, SymSim, TedSim, and scMultiSim-tree, exhibited poorly in terms of accuracy (Fig. 2) because they solely simulate data based on the provided topology structure and kinetic parameters, neglecting the characterization of gene expression and variability from the real data.

Well-performed methods in accuracy (e.g., scDesign3, ZINB-WaVE, SPARSim, Splat, and SCRIP-GP-trendedBCV, see Fig. 2) employ disparate approaches to accurately capture and interpret the sparsity and variability nature of scRNA-seq data. For example, ZINB-WaVE models the probability of dropouts in terms of cell- and gene-level covariates, which can accurately characterize the sparsity in scRNA-seq data. ZINB-WaVE also included a set of unobserved covariates to account for the presence of unwanted variation (e.g., batch effect) and the biological effect of interest (e.g., cell type). SPARSim considers the compositionality nature of RNA sequencing data [49], in which RNA capture and sequencing are the sampling process from a fixed sample size without replacement. By applying a multivariate hypergeometric distribution, SPARSim can explicitly model the biological and technical variability, as well as effectively reproduce the sparsity in terms of percentages of zeros. Another popular simulation method Splat learns the probability that a gene count should be a zero by exploring the relationship between the mean gene expression and the proportions of zeros through a logistic function. Furthermore, Splat enforces the mean–variance trend by utilizing the biological coefficient of variation (BCV) for each gene to simulate biological effects based on the dispersion estimated from real data. Built upon the model implemented in Splat, SCRIP-GP-trendedBCV describes the dependency between mean gene expression and BCV through a generalized additive model (GAM) to capture the biological variability from the real data, which can achieve the accurate simulation of data dispersion.

Conversely, simulation methods tend to demonstrate poorly in accuracy if the characteristics of real data are not comprehensively captured (e.g., scDD, BASiCS, Simple, Lun, scMultiSim, and VeloSim, see Fig. 2). As scDD samples from genes with less than 75% zero values, it can only simulate relatively highly expressed genes and lack the ability to characterize the high proportion of zeros in scRNA-seq data. BASiCS is capable of explicitly characterizing the unexplained technical variability or noise, by modeling the observed values of 92 extrinsic molecules derived by the External RNA Controls Consortium (ERCC). Currently, this is the most effective way to distinguish confounding technical variability of the data. Nevertheless, BASiCS does not characterize the data properties associated with zero values to account for sparsity. As a basic data simulation method, Simple only models the mean gene expression using a Gamma distribution and simulates genes with over-dispersion merely by customizing the dispersion parameter, which cannot accurately reproduce many properties of the real data, such as the library size of cells and the proportion of zeros per cell. Built upon the Simple, Lun adjusts the mean expression of each cell using a transformed scaling factor drawn from a normal distribution, which represents the contribution of technical variability to observed gene expression. However, it does not account for the sparsity and biological variability of scRNA-seq data. Some methods, such as scMultiSim, VeloSim, dyntoy, SymSim, and dyngen, only required the pre-defined trajectory topology or the tree-structured input which describes the differentiation relationship of cell types for data simulation. Thus, they ignore the strict characterization of essential data properties from real data and cannot generate simulated data that resembles the real one.

Our results also revealed that some simulation methods developed for scRNA-seq data have powerful compatibility to simulate SRT data (Fig. 4d). This is because most SRT and scRNA-seq data share common features in terms of gene expression and data characteristics, such as the excessive zeros and the over-dispersion [50]. Importantly, gene expression of most SRT data also follows the NB or Poisson distribution [50], which is similar to that of scRNA-seq data. Hence, based on these similarities, it is reasonable that the methods tailored to scRNA-seq data can also simulate reliable SRT data. In addition, the study by Zhao et al. has also confirmed that the over-dispersion and zero inflation observed in SRT data are primarily due to the heterogeneity of gene expression and spatial distribution of cell types across regions [50]. Thus, over-dispersion and zero inflation in SRT data can be regarded as biologically important information, which is concordant with the interpretation and understanding of scRNA-seq data [51, 52]. It is suggested that in some cases, developers can generally transfer the modeling strategy from scRNA-seq data to SRT data. However, caution should also be exercised to ascertain whether the method consistently demonstrates excellent performance and flexible applicability, given the SRT data derived from different technologies and experimental designs.

Some simulation algorithms may cause data distortion and over-simulation when generating datasets with ground truth. Methods such as Splat, SCRIP, Lun, and scMultiSim solely rely on the customized parameters to simulate the ground truth, without utilizing prior information (e.g., real labels of cell types and batches) to capture heterogeneity or variance effects in real data. Many gene- or cell-level data properties in the simulated data are controlled by customized parameters, such as the difference of gene expression levels, the number of cell groups and the degree of batch effects. However, excessively flexible parameter settings can easily distort the data, so that the simulated data cannot reflect the real biological changes or differences, which may lead to over-simulation and affect the accuracy of the method. In some cases, when users need to distinguish technical and biological variations of the data, they should be cautious in choosing these methods for simulating datasets with ground truth.

Moreover, a positive correlation between the scores of different functionalities was observed in our results (Fig. 6b and Additional file 1: Fig. S8), indicating a potential relationship between the performance of methods across different functionalities. For instance, the gene expression levels of DEGs between cell groups may influence the reliability of the simulated cell groups, because whether cell groups can be clearly distinguished largely depends on the extent of differences in the expression levels of DEGs. When there are substantial differences in gene expression, it can enhance the heterogeneity between simulated cell groups and result in a clear separation of different populations in the plots of *t*-distributed stochastic neighbor embedding (t-SNE) or uniform manifold approximation and projection (UMAP). Conversely, if the difference is not significant, the gene expression patterns of different cell groups will be more similar, thereby reducing the purity and distinctiveness of cell groups.

## Conclusions

Overall, the simulation methods built upon the optimal-chosen model (i.e., SRTsim and scDesign2) and GAMLSS (i.e., scDesign3) had the best accuracy performance. ZINB-WaVE, SPARSim, Splat, SCRIP-paths, muscat, and SCRIP-GP-trendedBCV also demonstrated competitive ability to simulate realistic datasets. Additionally, Splat, SPARSim, SCRIP, SplatPop, dropsim, and ZINB-WaVE had powerful compatibility for simulating SRT data. Lun was the best method for simulating data with explicit cell groups and reliable DEGs, while SPARSim and scDesign3-tree performed best in the simulation functionality of cell batches and trajectories, respectively. As no methods consistently outperformed others on all evaluation criteria, trade-offs should be carefully considered for users between accuracy and functionality, as well as accuracy and scalability. For usability, more than half of the methods would produce execution errors with considerably varied proportions. “Failed estimation for genes” and “Appearance of missing (infinite) values” are the two main causes of the execution failures. Based on the evaluation results, we provided practical guidelines, a standard simulation pipeline Simpipe and an interactive tool Simsite for users to select the suitable method and perform simulation tasks. Our study will help users to navigate the space of prevalent simulation methods, guide developers toward proposing more scalable and efficient methods and promote their understanding of the characteristics of gene expression data.

## Methods

### Simulation methods

We collected 49 simulation methods designed for scRNA-seq and SRT data under the survey of literature (Additional file 2: Table S1). Among them, SRTsim, scDesign3, scDesign3-tree, scMultiSim, and scMultiSim-tree were specifically developed for simulating SRT data. We classified the methods into 5 categories through two steps. First, the methods that were able to simulate trajectory data were gathered into a separate category, because the requirement of simulating cell trajectories should be initially considered when users intend to apply the method functionality based on the practical guideline (Fig. 7). Next, we divided the remaining methods into 4 classes, by the number of application scenarios where they can be applied. The classification of methods is shown below:

- Class1 methods can simulate data with cell differentiation trajectory;
- Class2 methods can be simultaneously applied to three simulation scenarios: simulation of cell groups (or spatial domains), DEGs, and cell batches;
- Class3 methods are limited to concurrent use in only two simulation scenarios;
- Class4 methods have only one useful simulation functionality;
- Class5 methods can only simulate gene expression data without having other functionalities.

Additionally, we unified the names of some key parameters across different methods, making them convenient and easy to set in our standard simulation pipeline. These parameters control the settings related to the simulated data size, cell groups, DEGs, cell batches, and the ERCC RNA spike-in. The detailed information of these parameters and settings in the R programming environment are illustrated in Additional file 1: Supplementary Note Sect. 1. The available parameters of each method are listed in Additional file 2: Table S2.

### Real reference datasets

In total, 152 real reference datasets (101 scRNA-seq and 51 SRT datasets) with raw counts were collected from 13 scRNA-seq platforms and 11 technologies for spatial transcriptomics. For data preprocessing, cells (or spots in spatial data) with zero count across all genes were removed. Given the absence of benchmark datasets with explicit spatial trajectory, we collected and processed 13 SRT datasets generated from 6 cancer types, since the process of cancer invasion and metastasis has been well known to follow a linear trajectory model [53, 54], which was also verified in our study (Additional file 1: Supplementary Note Sect. 2 and Additional file 1: Fig. S25). Among them, seven 10 × Visium datasets lack domain annotation, and deconvolution analysis was performed using SpaCET [55]. The annotation result was the cell type with the highest proportion in each spot. The detailed information of the datasets is available in Additional file 2: Table S3.

The reference datasets contain four types of prior information as input: (1) labels of cell groups; (2) labels of cell batches; (3) ERCC spike-in controls; (4) information of cellular differentiation trajectory, such as the milestone network, trajectory topology, and Newick string representing the differentiation process. For those methods that necessarily demand for the prior information as input, the datasets were selectively used based on the method functionality. For example, scDD requires the labels of cell groups as input for parameter estimation, thus only the reference datasets with explicit prior knowledge of cell groups were used.

### Parameter estimation and data simulation

Parameter estimation is a critical step for methods to extract and learn the characteristics from the real datasets. However, SPsimSeq, scDesign, and SimBPDD have merged the parameter estimation and data simulation steps, so their performances in the parameter estimation step are not evaluated. In addition, scMultiSim-tree, SymSim, VeloSim, PROSSTT, dyntoy, and dyngen do not have an independent function for estimating the parameters, so we defined the process of converting the raw reference datasets into the input data as their parameter estimation step (Additional file 1: Supplementary Note Sect. 3.3).

In the data simulation step, an important problem is how to fully use the information of the reference datasets (such as the number of cell groups and the proportion of DEGs) to conduct an objective and unbiased evaluation. Before the simulation, we extracted and prepared the input information related to cell groups, DEGs, and batches from the reference datasets, to ensure the consistency of some cell- or gene-level information between the simulated and real data in this study. For example, if the real data contains two cell batches and three cell groups, we will simulate datasets with the same number of cell batches and groups. Similarly, data consistency is ensured by setting the proportion of DEGs between cell groups. Specifically, the input information is optionally used depending on the parameters that the methods contain. Detailed descriptions of extracting information and setting parameters before the simulation are illustrated in Additional file 1: Supplementary Note Sect. 4.2.

Except for scGAN, all computations were performed under Ubuntu 20.04.6 LTS with Intel Xeon(R) W-2255 (3.70 GHz) processors and 256 GB of RAM using a single CPU core in R (Version 4.2.3). The evaluation of scGAN was implemented under the CenOS release 7.4.1708, and the computational process was accelerated by the NVIDIA GeForce RTX 2080 Ti GPU (12GB).

### Summary of data properties

Fifteen data properties (7 on the cell level and 8 on the gene level) were summarized to compare the similarity between the real and simulated datasets. Most of them are characterized by one-dimensional vectors (cell level: 6 data properties, gene level: 5 data properties). For the property of cell–cell correlation, the upper triangle matrix from the cell correlation matrix was extracted using the *upper.tri* function in R. To represent the intrinsic characteristics of gene expression matrices, we also considered the relationship of two properties (1 out of 7 on the cell level and 3 out of 8 on the gene level). The included data properties are shown below and the detailed information can be found in Additional file 2: Table S7.

- LS: Library Size per cell
- FZC: Fraction of Zeros per Cell
- CCC: Cell–Cell Correlation
- TMM: TMM normalization factor per cell
- ELS: Effective Library Size per cell
- FCO: Fraction of Cell Outliers
- RLZ: Relation of Library size and the fraction of Zeros in cells
- ME: Mean Expression values of genes
- SD: Standard Deviation of gene expression values
- CV: Coefficient of Variance of gene expression values
- FZG: Fraction of Zeros per Gene
- FGO: Fraction of Gene Outliers
- RMS: Relation of Mean expression and Standard variance values of genes
- RMZ: Relation of Mean expression values and the fraction of Zeros in genes
- RDM: Relation of the Dispersion values and Mean expression values of genes

### Accuracy evaluation

The accuracy of simulation methods was assessed using eight metrics to compare the similarity of data properties derived from the real and corresponding simulated data. For one-dimensional properties, the values in the numeric vectors were sorted in ascending order, and six metrics were calculated based on them, including median absolute deviation (MAD), Kolmogorov–Smirnov distance (KS distance), mean absolute error (MAE), root mean square error (RMSE), overlapping index (OV) [56], and Bhattacharyya distance (BH distance). For two-dimensional data properties, the multi-dimensional Kolmogorov–Smirnov test (multiKS) [57, 58] and the kernel-density based on two-sample comparison test (KDE) were performed. The metrics used for the accuracy evaluation were illustrated in detail in Additional file 1: Supplementary Note Sect. 5.1.

Specifically, we employed the variant versions of multiKS and KDE, termed “spatial-multiKS” and “spatial-KDE,” to account for the “spatial level” of accuracy for the simulated SRT data. They can quantify the similarity between the simulated and real SRT data by considering the spatial positions or coordinates (axes of *X* and *Y*) as two-dimensional features defined in both the real and simulated data. Unfortunately, except for SRTsim and scDesign3, other methods do not provide spatial positions for the simulated SRT data, which raised an inevitable challenge in calculating the metrics. To solve this problem, we adopted the Hungarian algorithm to match the simulated spatial spots with those spots in the real SRT data, so that the spatial coordinates of spots in real data can be transferred to the matched simulated spots.

### Functionality evaluation

#### Assessment of simulated cell groups/spatial domains

To quantify the reliability of simulated cell groups in scRNA-seq data or spatial domains in SRT data, we adopted six metrics that are commonly used to evaluate different aspects of clustering performance [59], including average silhouette width (ASW) [60], Dunn index, Connectivity [61], Davies-Bouldin index (DB index), clustering deviation index (CDI) [62], and ROUGE [63]. The detailed information of the metrics is described in Additional file 1: Supplementary Note Sect. 5.2.

#### Assessment of simulated cell batches

Seven metrics were used to measure the degree of batch effects in the simulated data, including cell-specific mixing score (CMS) [64], local inverse Simpson’s index (LISI) [65], mixing metric (MM) [66], Shannon entropy [67], kBET [68], average silhouette width for batch (ASW batch), and principal component regression score (PCR score) [68]. The five metrics, CMS, LISI, MM, Shannon entropy, and kBET, determined the degree of batch-wise diversity within the *k*-nearest neighborhood (KNN) graphs, while the remaining two metrics reflected the reliability of batch populations and contribution of batch effects in the simulated data. In the study, we set $k=\sqrt{n}$ (*n* is the cell number in the data) to construct KNN graphs [69]. The detailed information about these batch-specific metrics is illustrated in Additional file 1: Supplementary Note Sect. 5.3. It should be noted that the methods were expected to demonstrate powerful performance under this simulation scenario when the prominent batch effects were detected in the simulated data.

#### Assessment of simulated DEGs

Three approaches were applied to evaluate the ability of methods to simulate DEGs.

(1) We chose the most optimal algorithm (edgeRQLFDetRate, edgeR QLF model including the cellular detection rate [70]) that performed best in a previous benchmark study to identify DEGs [8]. All identified DEGs were considered as the ground truth. Then, the ratio of candidate DEGs (returned by the simulation method) to the true DEGs was calculated. For three or more groups, it was weighted by the proportion of DEGs from pairwise groups to all DEGs.

$$P=\left\{\begin{array}{lr}P^{true}&(n=2)\\\sum\nolimits_{i=1}^{n}\frac{P_{i}^{true}\cdot N_{i}^{given}}{N^{given}}&(n > 2)\end{array}\right.$$

where ${P}^{true}$ is the proportion of true DEGs identified from two cell groups through the edgeRQLFDetRate algorithm; ${N}_{i}^{given}$ denotes the DEG number returned by the simulation method from the $i$ th group pair; ${N}^{given}$ denotes the total DEG number of all group pairs.

(2) We hypothesized that if there are no DEGs between two-sample groups, the false positive rate of detected DEGs should be approximately 0.05 and *P* values should follow a uniform distribution [8]. Otherwise, the potential DEGs exist between pairwise groups. To determine the reliability of potential DEGs returned by the simulation methods, we directly removed them from the gene expression matrix and then the differential expression analysis was performed using edgeRQLFDetRate to obtain the *P* values. Next, we defined the null hypothesis that the *P* values follow a uniform distribution. The Pearson chi-squared test was then used to determine whether to reject the null hypothesis under the threshold ($P\le 0.05$). If the null hypothesis is accepted for the dataset, the distribution score is assigned to 1. For three or more groups, the DEGs in each pair of groups were removed and the null hypothesis was tested. Finally, the distribution score was defined as the mean values across all group pairs.

$$\mathrm{distribution}\;\mathrm{score}\;=\left\{\begin{array}{lc}0\;\mathrm{or}\;1&(n=2)\\\frac1{N}\sum\nolimits_{i=1}^{N}{S}_{i}&(n>2)\end{array}\right.$$

where ${S}_{i}$ is the distribution score of the $i$ th group pair; $N=\left(\genfrac{}{}{0pt}{}{n}{2}\right)$, which denotes the number of paired groups. $n$ is the number of cell groups.

(3) The third approach is to construct support vector machine (SVM) models using the simulated gene expression matrices. Genes with expression values of zero standard deviation were filtered out. All DEGs in the simulated gene expression matrix were then extracted to form a new dataset. We randomly split 80% of the new data as the training set and the remaining 20% as the test set. SVM models were trained using the radial kernel and tenfold cross-validation. Accuracy, precision, recall, F1 score and area under ROC curve (AUC) were applied to assess the model performance. For multi-class classification, macro-averaged precision, recall and F1 score were computed.

#### Assessment of simulated SVGs

For the simulated SRT data, the reliability of spatially variable genes (SVGs) was also assessed by the approaches used for the DEGs in simulated scRNA-seq data. Furthermore, we evaluated the simulated SVGs using Moran’s *I* statistics which are commonly used to quantify the degree of autocorrelation of gene expression in space [71–73]. The expression patterns of SVGs are supposed to exhibit high spatial autocorrelation if the expression values of spots have a strong relationship with those spots near them. Moran’s *I* statistics range from − 1 to 1, where a value close to 1 indicates a clear spatial pattern, a value close to 0 indicates the random gene expression and a value close to − 1 indicates a chessboard-like pattern.

#### Assessment of simulated trajectories

To quantify the similarity between the simulated and the real trajectory, we performed trajectory inference for the simulated data using Slingshot [74] and then applied four metrics proposed by Saelens et al. [17], including Hamming–Ipsen–Mikhailov distance (HIM), F1 score for branches (F1_branches_), F1 score for milestones (F1_milestones_), and Correlation between geodesic distances (Cor_dist_). Each of the four metrics targets a different aspect of the trajectory. HIM quantifies the similarity of two trajectories in terms of global topology and local degrees. The F1 scores reflect the arrangements of cells assigned to either branches (F1_branches_) or milestones (F1_milestones_) in both simulated and real trajectories. The metric Cor_dist_ calculates the correlation of relative distances of matched simulated and real cells. The detailed information about the metrics is described in Additional file 1: Supplementary Note Sect. 5.4.

### Scalability evaluation

To assess scalability, we up- and down-scaled seven reference datasets to generate 40 new datasets between 100 and 10,000 cells, and 500 and 10,000 genes (Additional file 2: Table S8). Time consumption and memory usage were monitored using the *peakRAM* function in the *peakRAM* R package. To alleviate the impact of irrelevant factors on the detection of memory usage and time consumption, each dataset was repeatedly tested three times. The maximum running memory was no more than 256 GB; otherwise, the missing values were produced.

Inspired by Saelens et al. [17], we built a generalized linear model: $y\approx {log}\left(x\right)+sqrt\left(x\right)+x+{x}^{2}+{x}^{3}$, to explore the relationship between the cell (gene) numbers and the execution time (memory usage). Here, $y$ denotes the time (s) or memory (MB), and $x$ denotes the cell or gene numbers. The trend and complexity of the relationship can be determined by the coefficient $coef$:

$$\left\{\begin{array}{ll} \text{sublinear}, & {coef}_{log(x)}\;>\;0.25\;\text{or}\;{coef}_{{sqrt}(x)}\;>\;0.25\\ \text{linear}, & {{coef}}_{x}\;>\;0.25\\ \text{quadratic}, & {{coef}}_{x^2}\;>\;0.25\\ \text{superquadratic}, & {{coef}}_{x^3}\;>\;0.25\\ \text{negative}\;\text{slope},& {coef}\;<\;0 \end{array}\right.$$

If no coefficient is above 0.25, the largest coefficient will be chosen. In addition, if there are two or more terms whose coefficients are above 0.25, the more complex trend is adopted.

Because of the high proportions of errors that occurred in certain methods such as BASiCS, there are limited training samples for these methods. Therefore, we united the datasets monitored within all executions and the scalability datasets detected from the pre-defined data with gradient cell or gene numbers. Next, we randomly split the combined datasets into the training (80%) and test data (20%) and applied the shape constrained additive model (SCAM) [42] and random forest (RF) to construct models for each method, in order to predict the execution time and memory usage when the cell and gene numbers are given. After that, we evaluated the models by calculating the Pearson correlation coefficients between the log10-transformed values of the predicted time (or memory) and the actual time (or memory).

The selection of the tree numbers in RF is of great importance for the model performance. To deal with this issue, we employed RF by setting different tree numbers (10, 20, 30, 40, 50, 60, 70, 80, 90, 100, 200, 300, 400, 500, 800, 1000, 3000, 4000, 5000) and repeated the procedure 10 times under the settings of each tree number. Finally, we chose 500 trees to grow in RF as the Pearson correlation coefficients showed high stability and robustness in this watershed (Additional file 1: Fig. S26-29).

### Usability evaluation

Based on the evaluation criteria and recommendations from nine authoritative references [33–41], we created a checklist of scoring rules (Additional file 2: Table S4) for usability evaluation, including method availability, code quality, self-evaluation, maintenance, documentation, and publication.

The method availability refers to whether the method is freely accessible to users, easy to install, and whether it requires any special equipment or hardware. The code quality covers the aspects of code modularization, classifications, and the implementation of unit testing. The self-evaluation category is frequently overlooked, which checks whether the evaluation or comparison has been performed between the newly developed methods and others in the original paper. Also, it assesses the test for the method sensitivity and the number of adopted criteria. In the maintenance category, we quantified the method in the aspects of the method updates, version control, and responses to the questions raised by users. The documentation of a method plays an essential role in usability for users. We quantified it in terms of the documentation interface, function descriptions, installation guides, and result presentations attached to the method. Finally, we assessed some aspects related to the publication in which the method was proposed, such as whether the manuscript has been peer-reviewed or not.

To obtain usability scores, firstly we allocated weights for the scoring items depending on the frequency they were mentioned in the nine references. We multiplied the manual scores by the corresponding weights to get the values and they were then summed up by each aspect of usability. Next, the values from the same aspect of usability were scaled to the standard normal distribution (*μ* = 0, *σ* = 1) and were subsequently transformed into (0,1) by the cumulative distribution function (CDF). The values of six aspects are visualized in Fig. 3e. The final usability score for each method was obtained (Fig. 2b) by calculating the arithmetic mean of values from the six aspects of usability.

### Score normalization and aggregation

We summarized the final scores of accuracy, functionality, and scalability by two stages of data processing. In the first stage, we normalized the scores on three levels: datasets, metrics, and data properties, to transform them into the range from 0 to 1. In particular, the raw measurements of scalability were initially log2-transformed before being processed in downstream steps. As an example of calculating the accuracy scores (Additional file 1: Fig. S30), we first scaled the scores from the same dataset across different methods to the standard normal distribution (*μ* = 0, *σ* = 1). They were then transformed into (0,1) by the CDF. As the values closing to 1 usually indicate higher accuracy, the values of some metrics therefore need to be subtracted from 1 to keep them harmonized. Subsequently, across different methods and datasets, we scaled the scores from the same data property and metric into [0, 1] by the following formula:

$$\frac{{x}_{m}^{p}-min({x}_{m}^{p})}{{max(x}_{m}^{p})-min({x}_{m}^{p})}$$

where ${x}_{m}^{p}$ indicates the scores of the data property $p$ on the metric $m$.

In the stage of score aggregation, we aggregated the scores across data properties, datasets and metrics, sequentially. In this way, we finally obtained the summarized scores of the accuracy criterion. Similarly, the process for aggregating functionality and scalability scores can be conducted following the above steps. However, the calculation process of the functionality or scalability scores does not involve the level of aggregating data properties.

To rank methods, the overall score for each method was calculated using the formula:

$$\text{overall score} = \frac{{\text{score}}_{\text{accuracy}}+ \text{ } {\text{score}}_{\text{functionality}}+ \text{ } {\text{score}}_{\text{scalability}}\text{+}{\text{ score}}_{\text{usability}}}{4}$$

Additionally, for methods without functionality, the following formula was adopted:

$$\text{overall score} = \frac{{\text{score}}_{\text{accuracy}}+ \text{ } {\text{score}}_{\text{scalability}}+ \text{ } {\text{score}}_{\text{usability}}}{3}$$

### Web-based vignettes, guidelines, and data simulation tool

We established a website (https://www.ciblab.net/software/Simsite/) to illustrate the usage of our simulation pipeline (https://github.com/duohongrui/simpipe) [75] by using rmarkdown (v2.23) and blogdown (v1.18) R packages. The online guideline of method selection and data simulation tool was built using golem (v0.4.1), shiny (v1.7.4.1) and shinythemes (v1.2.0) R packages, which can be accessed at https://www.ciblab.net/software/simshiny/. The local version of the online tool can also be installed in R from Github (https://github.com/duohongrui/simshiny) [76].

### Supplementary Information

Additional file 1. The file contains supplementary figures (Fig S1-S30), supplementary discussions and supplementary notes.

Additional file 2. The file contains supplementary tables (Table S1-S8).

Additional file 3. Review history.

## Data Availability

The reference scRNA-seq and SRT datasets analyzed during this study are available in the Zenodo repository (10.5281/zenodo.8251596) [84]. The detailed information of all 152 reference datasets is listed in Additional file 2: Table S3.

## References

[1] Xue R, Zhang Q, Cao Q, Kong R, Xiang X, Liu H, Feng M, Wang F, Cheng J, Li Z (2022). Liver tumour immune microenvironment subtypes and neutrophil heterogeneity. Nature.

[2] Rao A, Barkley D, Franca GS, Yanai I (2021). Exploring tissue architecture using spatial transcriptomics. Nature.

[3] Galeano Nino JL, Wu H, LaCourse KD, Kempchinsky AG, Baryiames A, Barber B, Futran N, Houlton J, Sather C, Sicinska E (2022). Effect of the intratumoral microbiota on spatial and cellular heterogeneity in cancer. Nature.

[4] Garcia-Alonso L, Lorenzi V, Mazzeo CI, Alves-Lopes JP, Roberts K, Sancho-Serra C, Engelbert J, Mareckova M, Gruhn WH, Botting RA (2022). Single-cell roadmap of human gonadal development. Nature.

[5] Kuppe C, Ramirez Flores RO, Li Z, Hayat S, Levinson RT, Liao X, Hannani MT, Tanevski J, Wunnemann F, Nagai JS (2022). Spatial multi-omic map of human myocardial infarction. Nature.

[6] Zappia L, Phipson B, Oshlack A (2018). Exploring the single-cell RNA-seq analysis landscape with the scRNA-tools database. PLoS Comput Biol.

[7] Luecken MD, Buttner M, Chaichoompu K, Danese A, Interlandi M, Mueller MF, Strobl DC, Zappia L, Dugas M, Colome-Tatche M, Theis FJ (2022). Benchmarking atlas-level data integration in single-cell genomics. Nat Methods.

[8] Soneson C, Robinson MD (2018). Bias, robustness and scalability in single-cell differential expression analysis. Nat Methods.

[9] Pratapa A, Jalihal AP, Law JN, Bharadwaj A, Murali TM (2020). Benchmarking algorithms for gene regulatory network inference from single-cell transcriptomic data. Nat Methods.

[10] Abdelaal T, Michielsen L, Cats D, Hoogduin D, Mei H, Reinders MJT, Mahfouz A (2019). A comparison of automatic cell identification methods for single-cell RNA sequencing data. Genome Biol.

[11] Yu L, Cao Y, Yang JYH, Yang P (2022). Benchmarking clustering algorithms on estimating the number of cell types from single-cell RNA-sequencing data. Genome Biol.

[12] Xi NM, Li JJ (2021). Benchmarking computational doublet-detection methods for single-cell RNA sequencing data. Cell Syst.

[13] Hou W, Ji Z, Ji H, Hicks SC (2020). A systematic evaluation of single-cell RNA-sequencing imputation methods. Genome Biol.

[14] Cole MB, Risso D, Wagner A, DeTomaso D, Ngai J, Purdom E, Dudoit S, Yosef N (2019). Performance assessment and selection of normalization procedures for single-cell RNA-seq. Cell Syst.

[15] Li B, Zhang W, Guo C, Xu H, Li L, Fang M, Hu Y, Zhang X, Yao X, Tang M (2022). Benchmarking spatial and single-cell transcriptomics integration methods for transcript distribution prediction and cell type deconvolution. Nat Methods.

[16] Ahlmann-Eltze C, Huber W (2023). Comparison of transformations for single-cell RNA-seq data. Nat Methods.

[17] Saelens W, Cannoodt R, Todorov H, Saeys Y (2019). A comparison of single-cell trajectory inference methods. Nat Biotechnol.

[18] Nguyen HCT, Baik B, Yoon S, Park T, Nam D (2023). Benchmarking integration of single-cell differential expression. Nat Commun.

[19] Junttila S, Smolander J, Elo LL (2022). Benchmarking methods for detecting differential states between conditions from multi-subject single-cell RNA-seq data. Briefings Bioinf..

[20] Zhu J, Shang L, Zhou X (2023). SRTsim: spatial pattern preserving simulations for spatially resolved transcriptomics. Genome Biol.

[21] Song D, Wang Q, Yan G, Liu T, Sun T, Li JJ (2024). scDesign3 generates realistic in silico data for multimodal single-cell and spatial omics. Nat Biotechnol.

[22] Li H, Zhang Z, Squires M, Chen X, Zhang X. scMultiSim: simulation of single cell multi-omics and spatial data guided by gene regulatory networks and cell-cell interactions. Research Square. 2023. 10.21203/rs.3.rs-3301625/v1. 10.1038/s41592-025-02651-0 PMC12226025 40247122

[23] Zappia L, Phipson B, Oshlack A (2017). Splatter: simulation of single-cell RNA sequencing data. Genome Biol.

[24] Vieth B, Ziegenhain C, Parekh S, Enard W, Hellmann I (2017). powsimR: power analysis for bulk and single cell RNA-seq experiments. Bioinformatics.

[25] Papadopoulos N, Gonzalo PR, Soding J (2019). PROSSTT: probabilistic simulation of single-cell RNA-seq data for complex differentiation processes. Bioinformatics.

[26] Li WV, Li JJ (2019). A statistical simulator scDesign for rational scRNA-seq experimental design. Bioinformatics.

[27] Assefa AT, Vandesompele J, Thas O (2020). SPsimSeq: semi-parametric simulation of bulk and single-cell RNA-sequencing data. Bioinformatics.

[28] Marouf M, Machart P, Bansal V, Kilian C, Magruder DS, Krebs CF, Bonn S (2020). Realistic in silico generation and augmentation of single-cell RNA-seq data using generative adversarial networks. Nat Commun.

[29] Cannoodt R, Saelens W, Deconinck L, Saeys Y (2021). Spearheading future omics analyses using dyngen, a multi-modal simulator of single cells. Nat Commun.

[30] Zhang Z, Zhang X. VeloSim: simulating single cell gene-expression and RNA velocity. bioRxiv. 2021. 10.1101/2021.01.11.426277.

[31] Cao Y, Yang P, Yang JYH (2021). A benchmark study of simulation methods for single-cell RNA sequencing data. Nat Commun.

[32] Crowell HL, Morillo Leonardo SX, Soneson C, Robinson MD (2023). The shaky foundations of simulating single-cell RNA sequencing data. Genome Biol.

[33] Gannon F (2001). The essential role of peer review. EMBO Rep.

[34] Wilson G, Aruliah DA, Brown CT, Chue Hong NP, Davis M, Guy RT, Haddock SH, Huff KD, Mitchell IM, Plumbley MD (2014). Best practices for scientific computing. PLoS Biol.

[35] Boulesteix AL (2015). Ten simple rules for reducing overoptimistic reporting in methodological computational research. PLoS Comput Biol.

[36] Artaza H, Chue Hong N, Corpas M, Corpuz A, Hooft R, Jimenez RC, Leskosek B, Olivier BG, Stourac J, SvobodovaVarekova R (2016). Top 10 metrics for life science software good practices. F1000Res.

[37] JimÈnez R, Kuzak M, Alhamdoosh M, Barker M, Batut B, Borg M, Capella-Gutierrez S, Chue Hong N, Cook M, Corpas M (2017). Four simple recommendations to encourage best practices in research software. F1000Res.

[38] Silva LB, Jimenez RC, Blomberg N, Luis Oliveira J (2017). General guidelines for biomedical software development. F1000Res.

[39] Taschuk M, Wilson G (2017). Ten simple rules for making research software more robust. PLoS Comput Biol.

[40] Karimzadeh M, Hoffman MM (2018). Top considerations for creating bioinformatics software documentation. Briefings Bioinf.

[41] Beaulieu-Jones BK, Greene CS (2017). Reproducibility of computational workflows is automated using continuous analysis. Nat Biotechnol.

[42] Pya N, Wood SN (2015). Shape constrained additive models. Stat Comput.

[43] Chen W, Li Y, Easton J, Finkelstein D, Wu G, Chen X (2018). UMI-count modeling and differential expression analysis for single-cell RNA sequencing. Genome Biol.

[44] Grun D, Kester L, van Oudenaarden A (2014). Validation of noise models for single-cell transcriptomics. Nat Methods.

[45] Magali S, Davide C, Stefan S, Alexander van O, Tarjei SM. Characterization of directed differentiation by high-throughput single-cell RNA-Seq. bioRxiv. 2014. 10.1101/003236.

[46] Ziegenhain C, Vieth B, Parekh S, Reinius B, Guillaumet-Adkins A, Smets M, Leonhardt H, Heyn H, Hellmann I, Enard W (2017). Comparative analysis of single-cell RNA sequencing methods. Mol Cell.

[47] Jiang R, Sun T, Song D, Li JJ (2022). Statistics or biology: the zero-inflation controversy about scRNA-seq data. Genome Biol.

[48] Dharmaratne M, Kulkarni AS, TaherianFard A, Mar JC (2022). scShapes: a statistical framework for identifying distribution shapes in single-cell RNA-sequencing data. GigaScience..

[49] Quinn TP, Erb I, Richardson MF, Crowley TM (2018). Understanding sequencing data as compositions: an outlook and review. Bioinformatics.

[50] Zhao P, Zhu J, Ma Y, Zhou X (2022). Modeling zero inflation is not necessary for spatial transcriptomics. Genome Biol.

[51] Svensson V (2020). Droplet scRNA-seq is not zero-inflated. Nat Biotechnol.

[52] Choi K, Chen Y, Skelly DA, Churchill GA (2020). Bayesian model selection reveals biological origins of zero inflation in single-cell transcriptomics. Genome Biol.

[53] Ren Y, Huang Z, Zhou L, Xiao P, Song J, He P, Xie C, Zhou R, Li M, Dong X (2023). Spatial transcriptomics reveals niche-specific enrichment and vulnerabilities of radial glial stem-like cells in malignant gliomas. Nat Commun.

[54] Shang L, Zhou X (2022). Spatially aware dimension reduction for spatial transcriptomics. Nat Commun.

[55] Ru B, Huang J, Zhang Y, Aldape K, Jiang P (2023). Estimation of cell lineages in tumors from spatial transcriptomics data. Nat Commun.

[56] Pastore M, Calcagni A (2019). Measuring distribution similarities between samples: a distribution-free overlapping index. Front Psychol.

[57] Fasano G, Franceschini A (1987). A multidimensional version of the Kolmogorov-Smirnov test. Mon Not R Astron Soc.

[58] Puritz C, Ness-Cohn E, Braun R. fasano. franceschini. test: an implementation of a multidimensional KS test in R. arXiv. 2021. 10.48550/arXiv.2106.10539.

[59] Liu T, Fang ZY, Zhang Z, Yu Y, Li M, Yin MZ (2024). A comprehensive overview of graph neural network-based approaches to clustering for spatial transcriptomics. Comput Struct Biotechnol J.

[60] Rousseeuw PJ (1987). Silhouettes: a graphical aid to the interpretation and validation of cluster analysis. J Comput Appl Math.

[61] Brock G, Pihur V, Datta S, Datta S (2008). clValid: an R package for cluster validation. J Stat Softw.

[62] Fang J, Chan C, Owzar K, Wang L, Qin D, Li QJ, Xie J (2022). Clustering Deviation Index (CDI): a robust and accurate internal measure for evaluating scRNA-seq data clustering. Genome Biol.

[63] Liu B, Li C, Li Z, Wang D, Ren X, Zhang Z (2020). An entropy-based metric for assessing the purity of single cell populations. Nat Commun.

[64] Lütge A, Zyprych-Walczak J, Kunzmann UB, Crowell HL, Calini D, Malhotra D, Soneson C, Robinson MD (2021). Cell MixS: quantifying and visualizing batch effects in single-cell RNA-seq data. Life Sci Alliance.

[65] Korsunsky I, Millard N, Fan J, Slowikowski K, Zhang F, Wei K, Baglaenko Y, Brenner M, Loh PR, Raychaudhuri S (2019). Fast, sensitive and accurate integration of single-cell data with Harmony. Nat Methods.

[66] Stuart T, Butler A, Hoffman P, Hafemeister C, Papalexi E, Mauck WM, Hao Y, Stoeckius M, Smibert P, Satija R (2019). Comprehensive integration of single-cell data. Cell.

[67] Chazarra-Gil R, van Dongen S, Kiselev VY, Hemberg M (2021). Flexible comparison of batch correction methods for single-cell RNA-seq using BatchBench. Nucleic Acids Res.

[68] Buttner M, Miao Z, Wolf FA, Teichmann SA, Theis FJ (2019). A test metric for assessing single-cell RNA-seq batch correction. Nat Methods.

[69] Sanchis-Segura C, Ibanez-Gual MV, Aguirre N, Cruz-Gomez AJ, Forn C (2020). Effects of different intracranial volume correction methods on univariate sex differences in grey matter volume and multivariate sex prediction. Sci Rep.

[70] Robinson MD, McCarthy DJ, Smyth GK (2010). edgeR: a Bioconductor package for differential expression analysis of digital gene expression data. Bioinformatics.

[71] Hu J, Li X, Coleman K, Schroeder A, Ma N, Irwin DJ, Lee EB, Shinohara RT, Li M (2021). SpaGCN: Integrating gene expression, spatial location and histology to identify spatial domains and spatially variable genes by graph convolutional network. Nat Methods.

[72] Shi X, Zhu J, Long Y, Liang C (2023). Identifying spatial domains of spatially resolved transcriptomics via multi-view graph convolutional networks. Brief Bioinform..

[73] Lin X, Gao L, Whitener N, Ahmed A, Wei Z (2022). A model-based constrained deep learning clustering approach for spatially resolved single-cell data. Genome Res.

[74] Street K, Risso D, Fletcher RB, Das D, Ngai J, Yosef N, Purdom E, Dudoit S (2018). Slingshot: cell lineage and pseudotime inference for single-cell transcriptomics. BMC Genomics.

[75] Duo HR, Li YH, Lan Y, Tao JX, Yang QX, Xiao YX, Sun J, Li L, Nie XE, Zhang XX, et al. Simpipe. Github. 2024. https://github.com/duohongrui/simpipe.

[76] Duo HR, Li YH, Lan Y, Tao JX, Yang QX, Xiao YX, Sun J, Li L, Nie XE, Zhang XX, et al. Simshiny. Github. 2024. https://github.com/duohongrui/simshiny.

[77] Duo HR, Li YH, Lan Y, Tao JX, Yang QX, Xiao YX, Sun J, Li L, Nie XE, Zhang XX, et al. Simbenchmark. Github. 2024. https://github.com/duohongrui/simbenchmark.

[78] Duo HR, Li YH, Lan Y, Tao JX, Yang QX, Xiao YX, Sun J, Li L, Nie XE, Zhang XX, et al. Simbenchmark. Zenodo. 2024. 10.5281/zenodo.11178453.

[79] Duo HR, Li YH, Lan Y, Tao JX, Yang QX, Xiao YX, Sun J, Li L, Nie XE, Zhang XX, et al. Simmethods. Github. 2024. https://github.com/duohongrui/simmethods.

[80] Duo HR, Li YH, Lan Y, Tao JX, Yang QX, Xiao YX, Sun J, Li L, Nie XE, Zhang XX, et al. Simmethods. Zenodo. 2024. 10.5281/zenodo.11179432.

[81] Duo HR, Li YH, Lan Y, Tao JX, Yang QX, Xiao YX, Sun J, Li L, Nie XE, Zhang XX, et al. Simpipe. 2024. Zenodo. 10.5281/zenodo.11178409.

[82] Duo HR, Li YH, Lan Y, Tao JX, Yang QX, Xiao YX, Sun J, Li L, Nie XE, Zhang XX, et al. Simpipe2docker. Github. 2024. https://github.com/duohongrui/simpipe2docker.

[83] Duo HR, Li YH, Lan Y, Tao JX, Yang QX, Xiao YX, Sun J, Li L, Nie XE, Zhang XX, et al. Simpipe2docker. 2024. Zenodo. 10.5281/zenodo.11179420.

[84] Duo HR, Li YH, Lan Y, Tao JX, Yang QX, Xiao YX, Sun J, Li L, Nie XE, Zhang XX, et al. Systematic evaluation with practical guidelines for single-cell and spatially resolved transcriptomics data simulation under multiple scenarios. Datasets. Zenodo. 2024. 10.5281/zenodo.8251596. 10.1186/s13059-024-03290-y PMC11149245 38831386

