# Supplementary material for: Systematic evaluation with practical guidelines for single-cell and spatially resolved transcriptomics data simulation under multiple scenarios
Source: Genome Biol. 2024 Jun 3;25:145. doi: 10.1186/s13059-024-03290-y (PMC11149245; doi:10.1186/s13059-024-03290-y)
Supplement: Supplementary file 1 — Additional file 1. The file contains supplementary figures (Fig S1-S30), supplementary discussions and supplementary notes. [file 13059_2024_3290_MOESM1_ESM.docx]

**Additional file 1**

**Systematic evaluation with practical guidelines for single-cell and spatially resolved transcriptomics data simulation under multiple scenarios**

Hongrui Duo^1,†^, Yinghong Li^2,†^, Yang Lan^3,†^, Jingxin Tao^1^, Qingxia Yang^4^, Yingxue Xiao^1^, Jing Sun^1^, Lei Li^1^, Xiner Nie^5^, Xiaoxi Zhang^1^, Guizhao Liang^5^, Mingwei Liu^6^, Youjin Hao^1,*^, Bo Li^1,*^

^1^College of Life Sciences, Chongqing Normal University, Chongqing 401331, P. R. China;

^2^Chongqing Key Laboratory of Big Data for Bio Intelligence, Chongqing University of Posts and Telecommunications, Chongqing 400065, P. R. China;

^3^Institute of Pathology and Southwest Cancer Center, Southwest Hospital, Army Medical University, Chongqing, 400038, P. R. China;

^4^Zhejiang Provincial Key Laboratory of Precision Diagnosis and Therapy for Major Gynecological Diseases, Women's Hospital, Zhejiang University School of Medicine, Hangzhou 310058, P. R. China;

^5^Key Laboratory of Biorheological Science and Technology, Ministry of Education, Bioengineering College, Chongqing University, Chongqing, 400044, P. R. China;

^6^Key Laboratory of Clinical Laboratory Diagnostics, College of Laboratory Medicine, Chongqing Medical University, Chongqing 400016, P. R. China.

^†^These authors contributed equally to this work as co-first authors: Hongrui Duo, Yinghong Li and Yang Lan.

^*^Correspondence should be addressed to Bo Li (libcell@cqnu.edu.cn) or Youjin Hao (haoyoujin@hotmail.com).

**List of Additional file 1**

[Supplementary Figure 1](#_Toc166596714)

[Supplementary Discussion 31](#_Toc166596715)

[**Discussion S1 The negative slope of trend complexity of scalability.** 31](#_Toc166596716)

[**Discussion S2 Quadratic complexity of time with varying gene numbers for scGAN in the data simulation step.** 31](#_Toc166596717)

[Supplementary Note 32](#_Toc166596718)

[**1 Unifying parameter names for all methods** 32](#_Toc166596719)

[**2 Datasets with cell differentiation trajectory** 33](#_Toc166596720)

[**3 Parameter estimation from real datasets** 34](#_Toc166596721)

[**3.1 Principles of parameter estimation** 34](#_Toc166596722)

[**3.2 Prior information required for parameter estimation** 35](#_Toc166596723)

[**3.3 Parameter estimation for the datasets with cell trajectory** 35](#_Toc166596724)

[**4 Data simulation** 36](#_Toc166596725)

[**4.1 Principles of the data simulation process** 36](#_Toc166596726)

[**4.2 Preparing essential information for simulation input** 36](#_Toc166596727)

[**4.3 Output of the simulated data** 37](#_Toc166596728)

[**5 Evaluation metrics** 37](#_Toc166596729)

[**5.1 Metrics for accuracy criterion** 37](#_Toc166596730)

[**5.2 Metrics for assessment of simulated cell groups/spatial domains** 39](#_Toc166596731)

[**5.3 Metrics for assessment of simulated cell batches** 41](#_Toc166596732)

[**5.4 Metrics for assessment of simulated trajectories** 42](#_Toc166596733)

# Supplementary Figure





**Fig S1 Overview of datasets and simulation methods.**

**a**, Pie plot showing the number and proportion of scRNA-seq datasets generated by 13 platforms. ‘Mix sources1’ indicates the data derived from two experimental platforms (Smart-seq2 and 10× Genomics). ‘Mix sources2’ indicates the mixture data derived from platforms of CEL-seq and CEL-seq2. **b**, Pie plot showing the number and proportion of SRT datasets generated by 11 platforms. **c**, The number of scRNA-seq datasets based on the two quantification strategies for counts. **d**, Scatter plot showing the cell and gene numbers of datasets derived from different platforms. **e**, Pie plot showing the number and proportion of methods for each algorithm model. The optimal-chosen model is defined as the algorithm used to determine the best-fit statistical distribution for each gene from four candidate distributions, including Poisson, zero-inflated Poisson (ZIP), negative binomial (NB, i.e., GP) and ZINB distribution. **f**, Number of methods in each functionality. Notably, some methods may possess more than two simulation functionalities, such as Splat and SPsimSeq. GP, gamma-Poisson; BGP, beta-gamma-Poisson; BP, beta-Poisson; GN, Gamma-Normal; GAMLSS, generalized additive model for location, scale and shape; GAN, generative adversarial network.


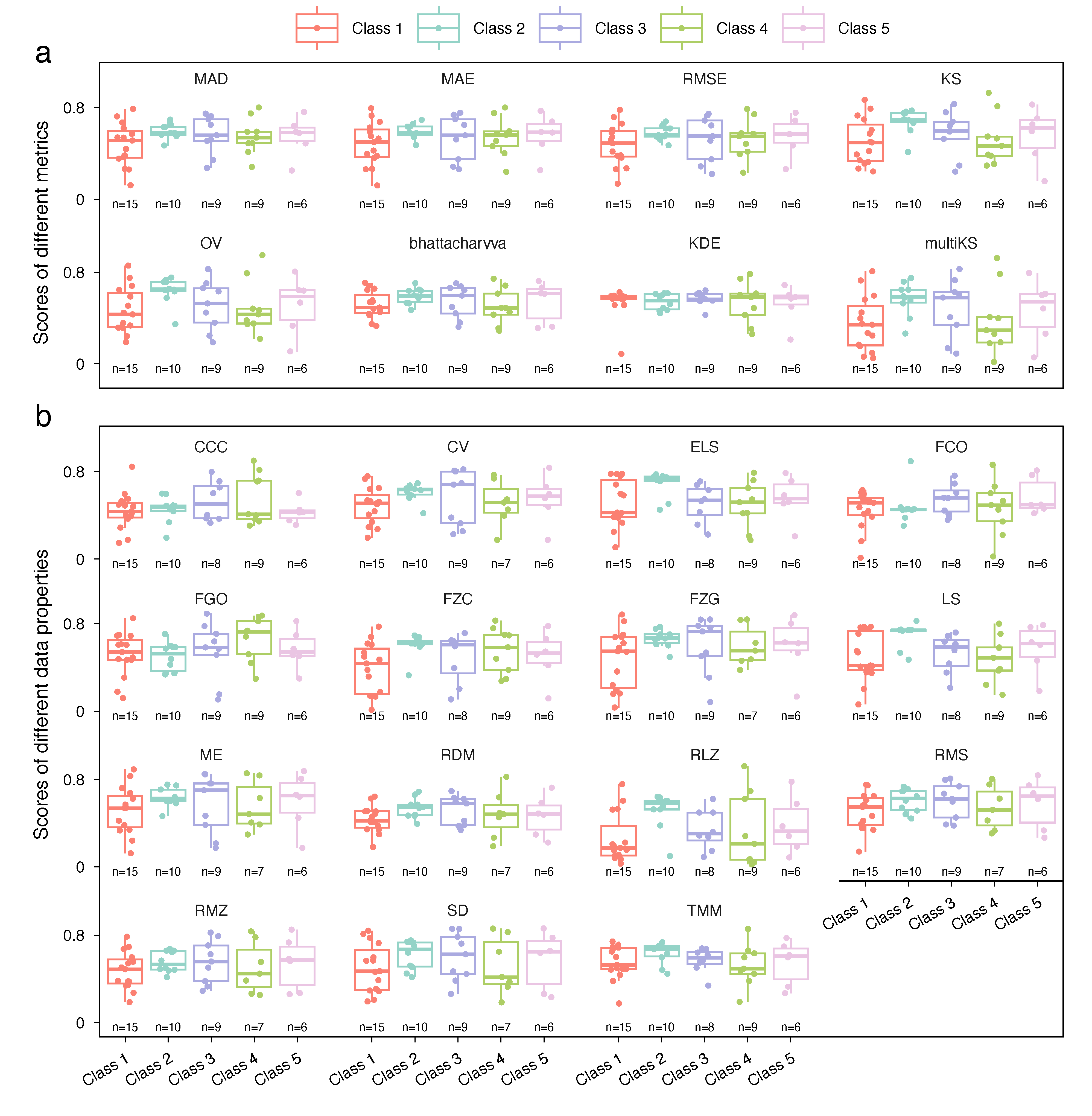


**Fig S2 Accuracy scores of metrics and data properties for each method class.**

The lower and upper hinges correspond to the first and third quartiles, and the center refers to the median value. The upper (lower) whiskers extend from the hinge to the largest (smallest) value no further (at most) than 1.5 × interquartile range from the hinge. Each dot represents one method belonging to the class. The sample size (n) is shown below the boxplots.


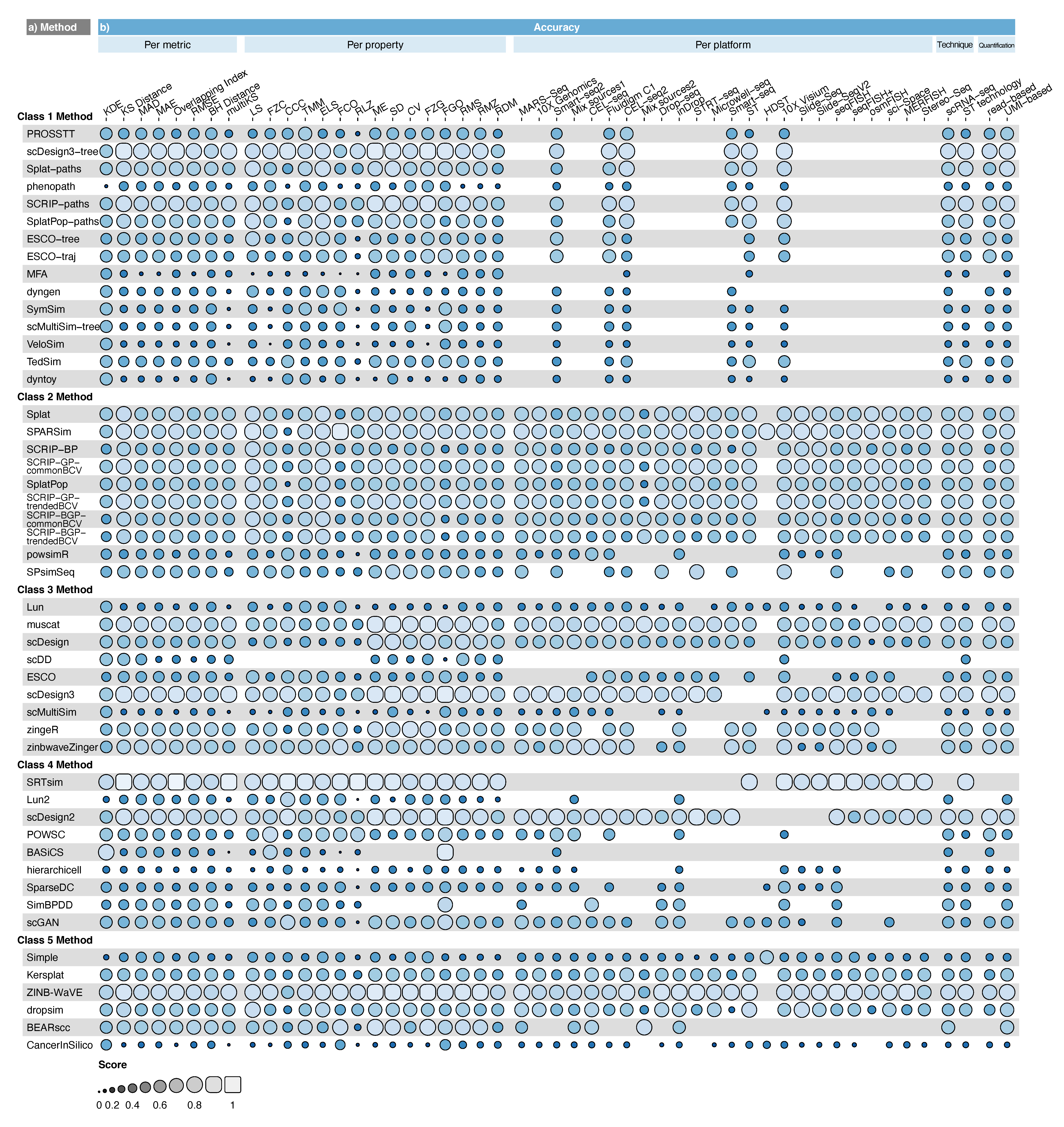


**Fig S3 Detailed accuracy performance of methods.**

**a**, Simulation methods are ranked based on the overall scores in each class. **b**, Accuracy scores of methods on different metrics, data properties, platforms, techniques and quantification strategies of counts.


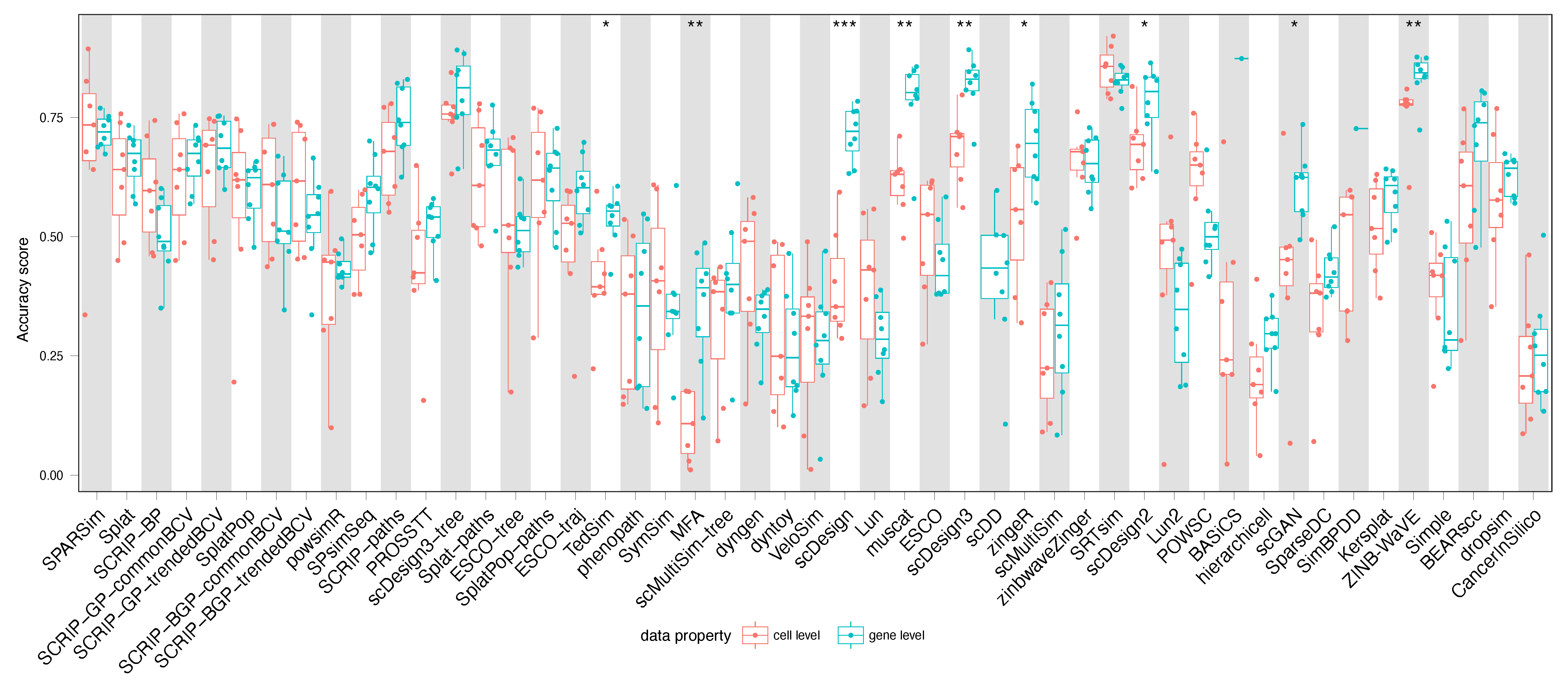


**Fig S4 Accuracy scores of methods on gene-level and cell-level data properties.**

The two-sided Wilcoxon test was performed between the accuracy scores of gene-level and cell-level data properties. *, *P*<0.05; **, *P*<0.01; ***, *P*<0.001; n=7 for cell-level properties; n=8 for gene-level properties; n=1 for gene-level properties in BASiCS and SimBPDD.


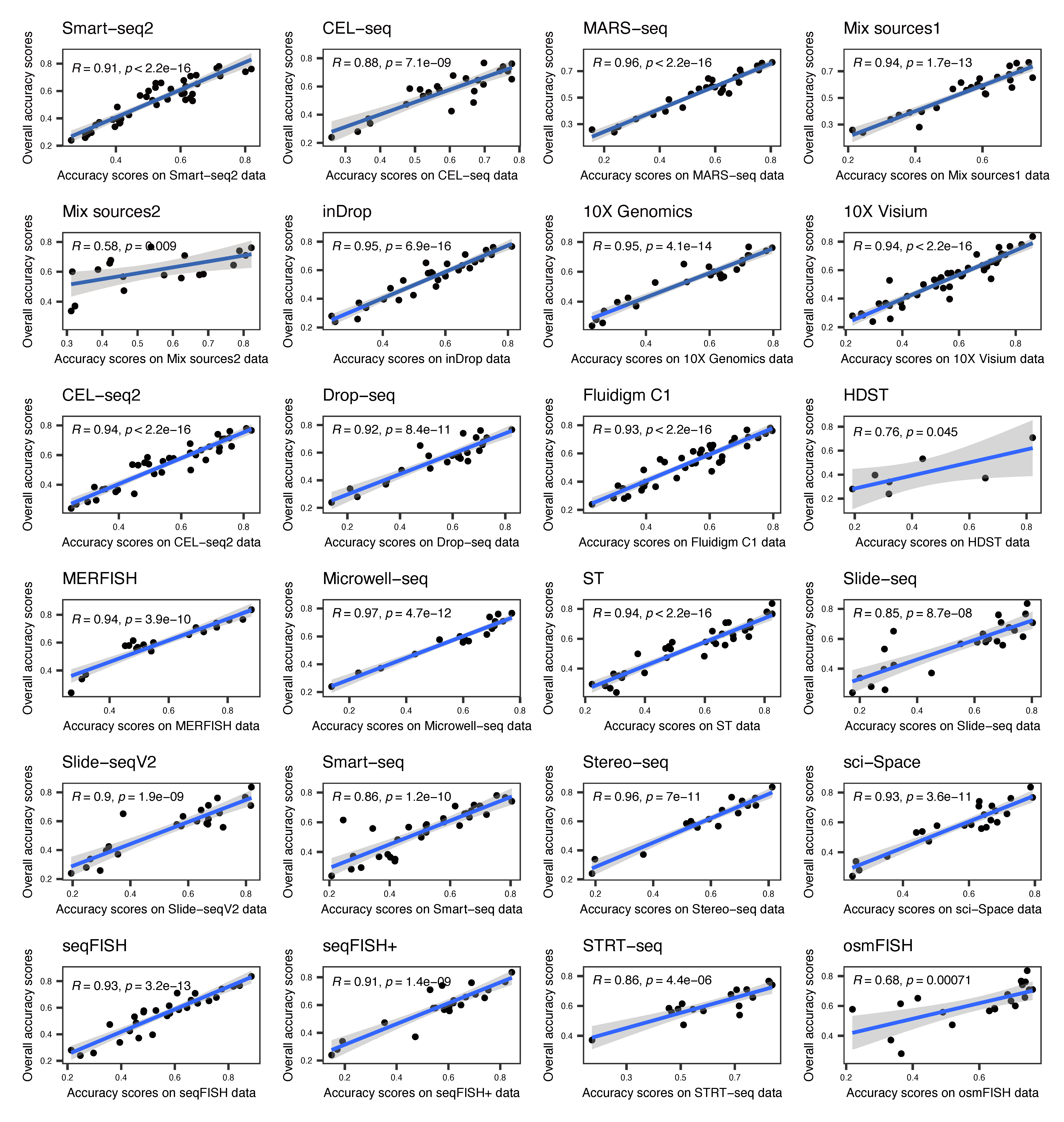


**Fig S5 Correlation between the scores on overall accuracy and each platform.**

‘Mix sources1’ indicates the data derived from two experimental platforms (Smart-seq2 and 10× Genomics). ‘Mix sources2’ indicates the mixture data derived from platforms of CEL-seq and CEL-seq2. The Pearson correlation coefficients and *P* values were calculated. The 95% confidence interval of the fitted line was marked in grey.

**
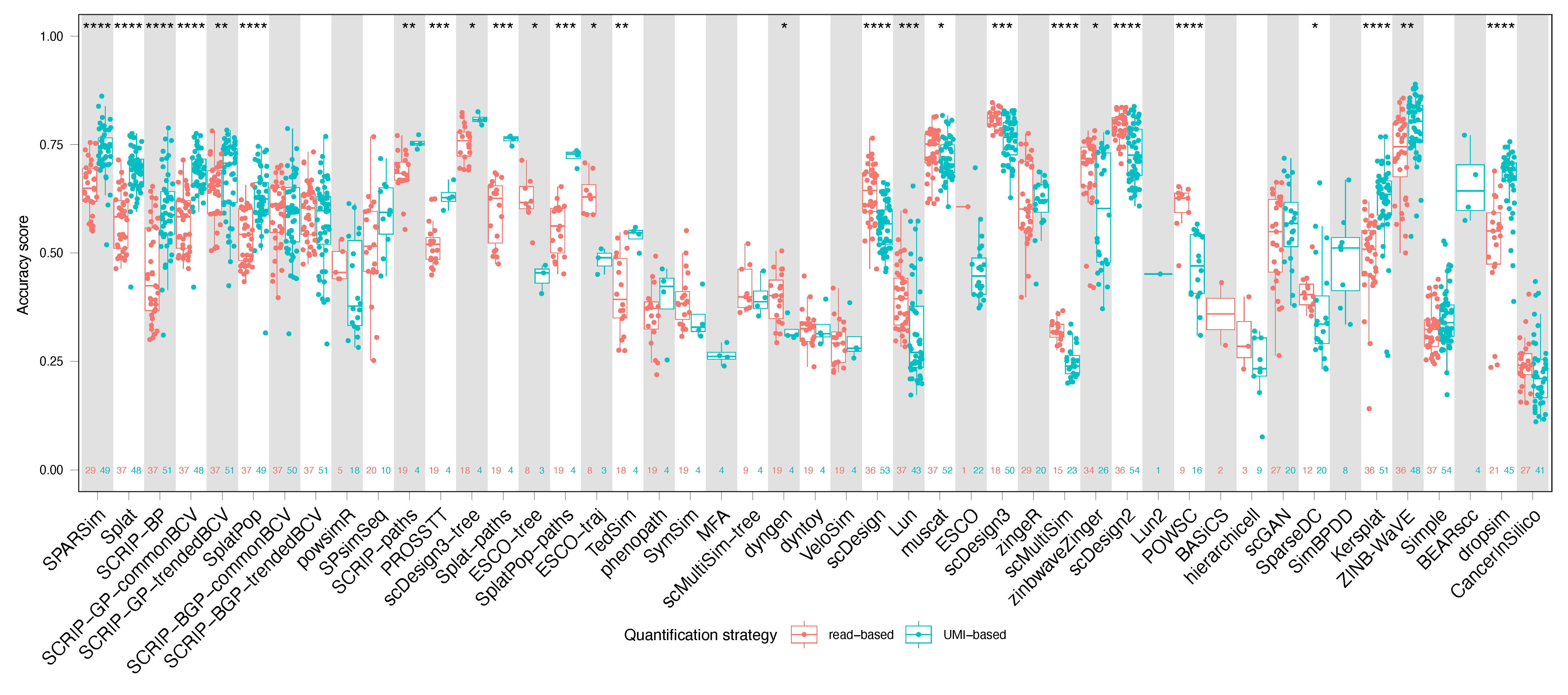
**

**Fig S6 Accuracy scores of methods on read-based and UMI-based quantification strategies of scRNA-seq data.**

The two-sided Wilcoxon test was performed between the accuracy scores on the read-based and UMI-based quantification strategies. The sample size (n) is shown below the boxplots. ^*^, *P*<0.05; ^**^, *P*<0.01; ^***^, *P*<0.001; ^****^, *P*<0.0001.

**
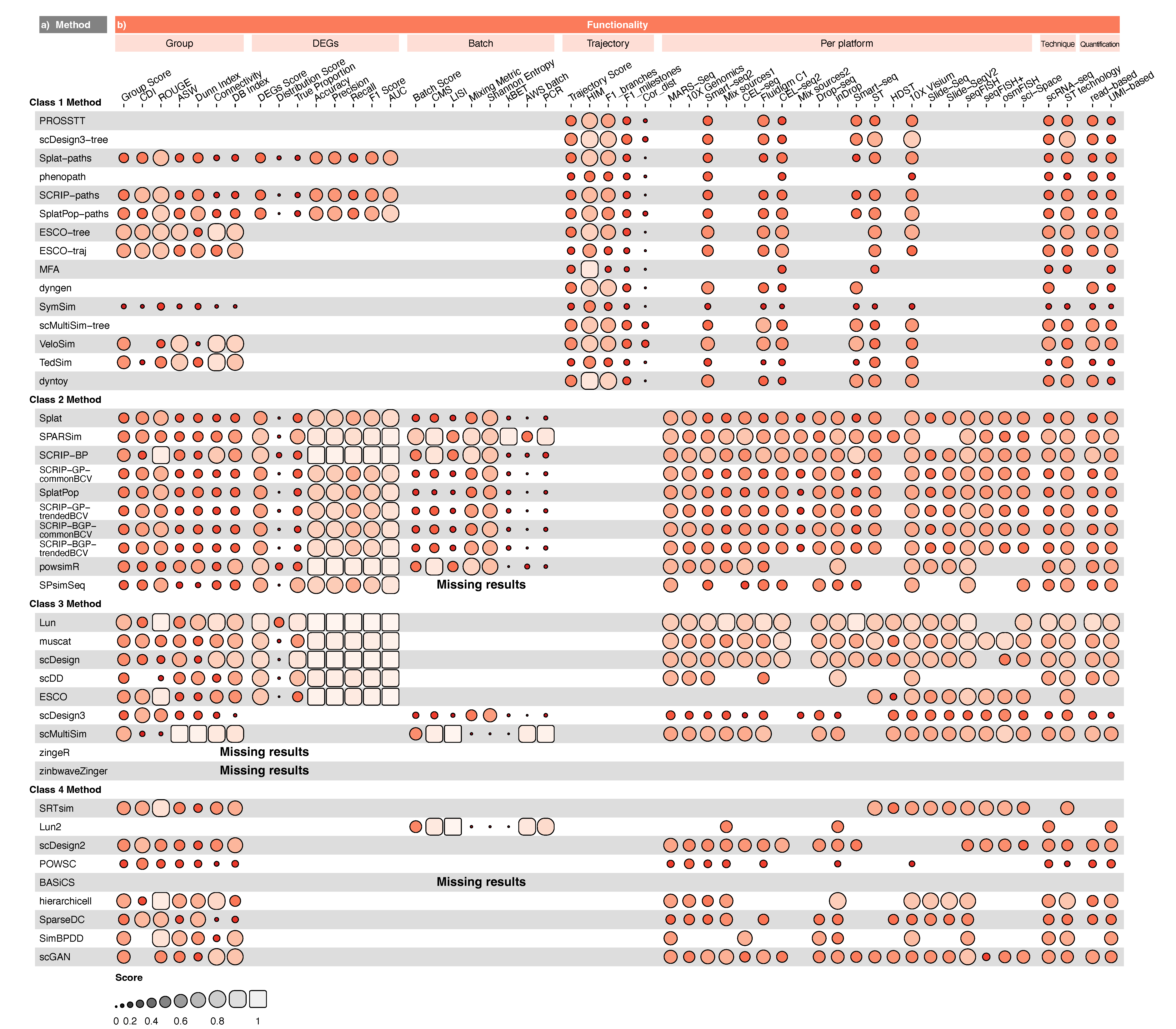
**

**Fig S7 Detailed functionality performance of methods.**

**a**, The simulation methods were ranked according to their overall scores in each class. **b**, Functionality scores of methods on different metrics, platforms, techniques and quantification strategies of counts. Due to the lack of simulated cell group labels in zingeR and zinbwaveZinger, evaluation results on the simulation functionalities of cell groups and DEGs were not available. In addition, SPsimSeq and BASiCS produced errors on the reference datasets with batch effects, which led to the missing results on the functionality performance of simulating cell batches.


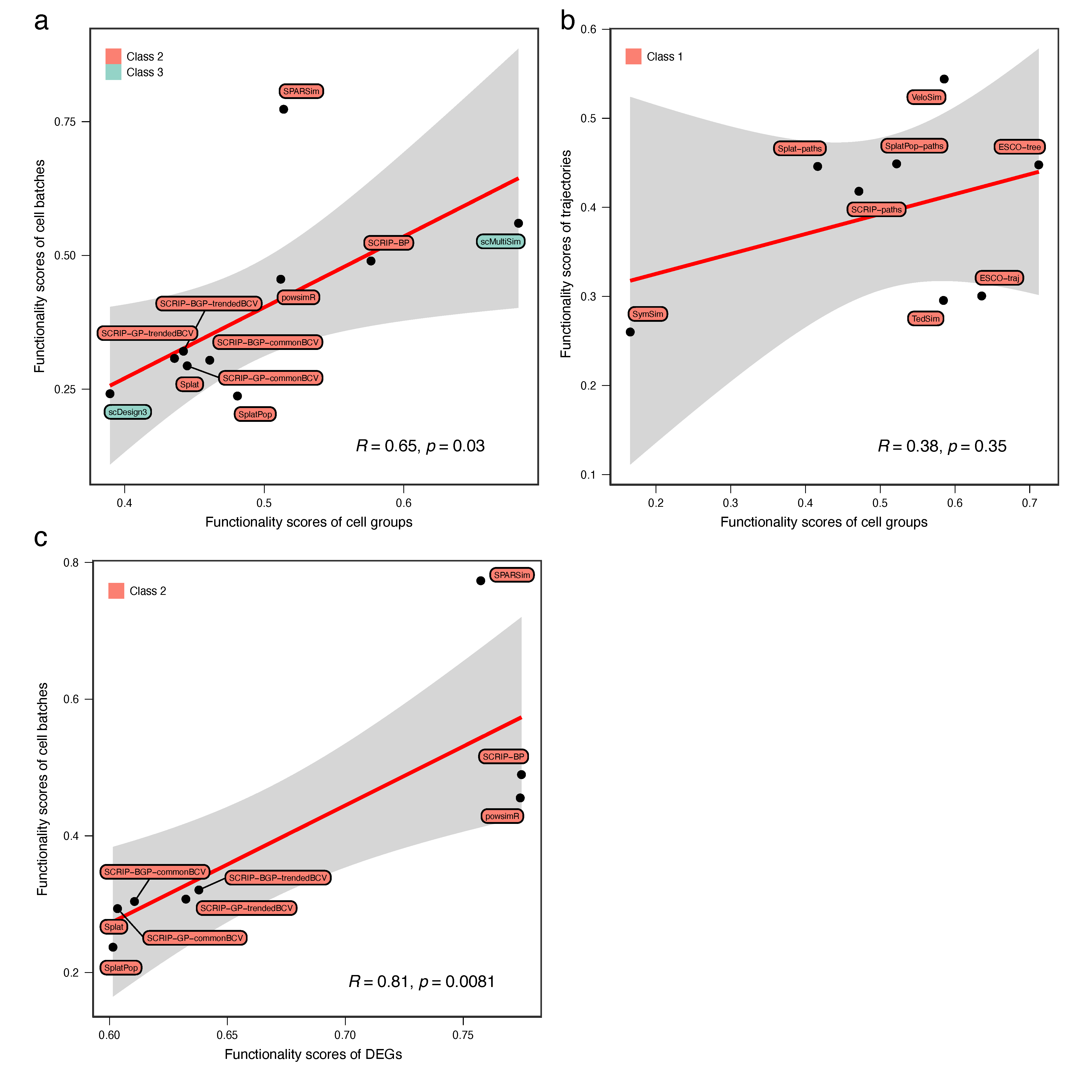


**Fig S8 Correlation between the scores of different method functionalities.**

The correlation between the functionality scores of the simulated cell groups and cell batches (**a**), cell groups and trajectories (**b**), or DEGs and cell batches (**c**). The Pearson correlation coefficient and *P* value were calculated. The 95% confidence interval of the fitted line was marked in grey.


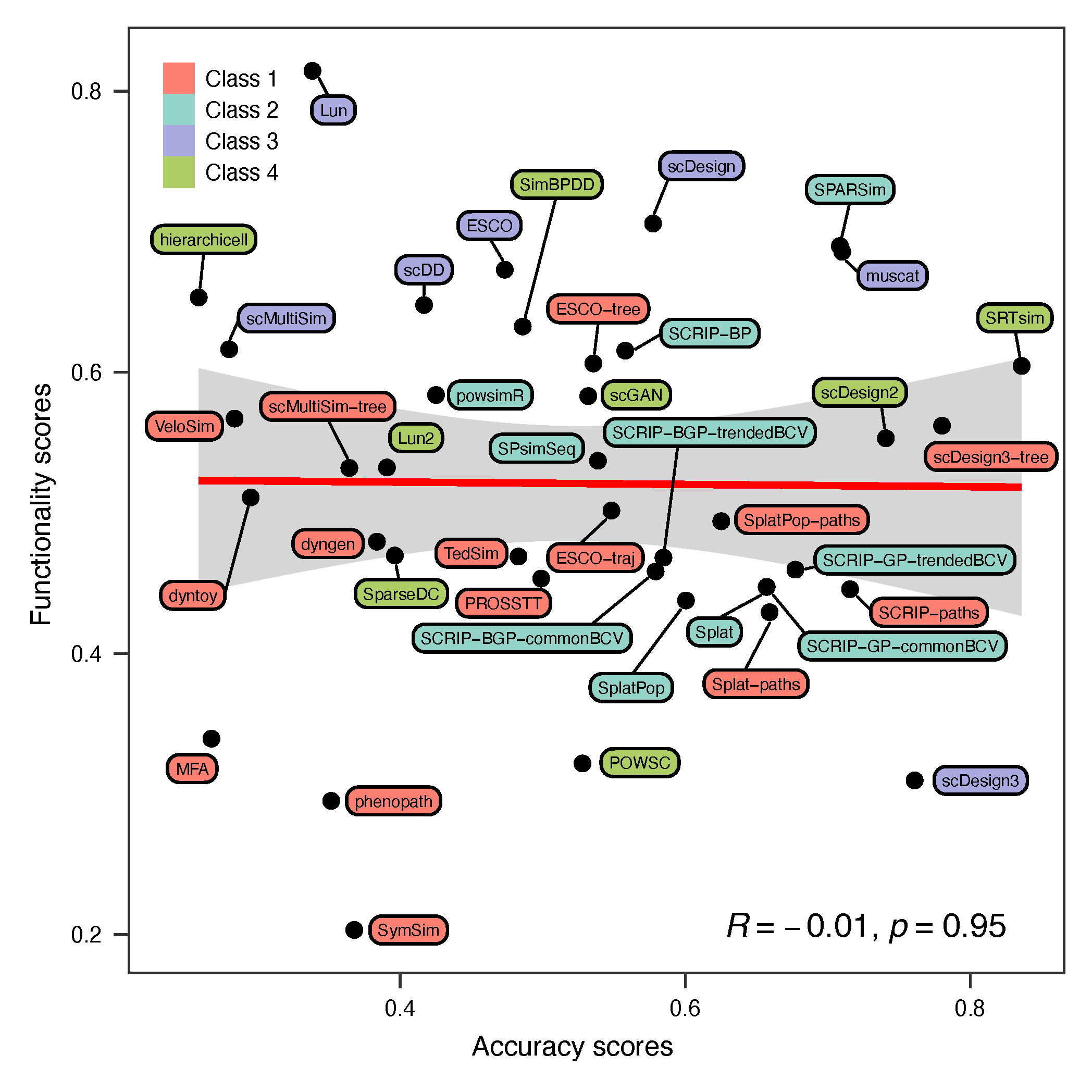


**Fig S9 Correlation between the scores of accuracy and functionality.**

The Pearson correlation coefficient and *P* value were calculated. The 95% confidence interval of the fitted line was marked in grey.


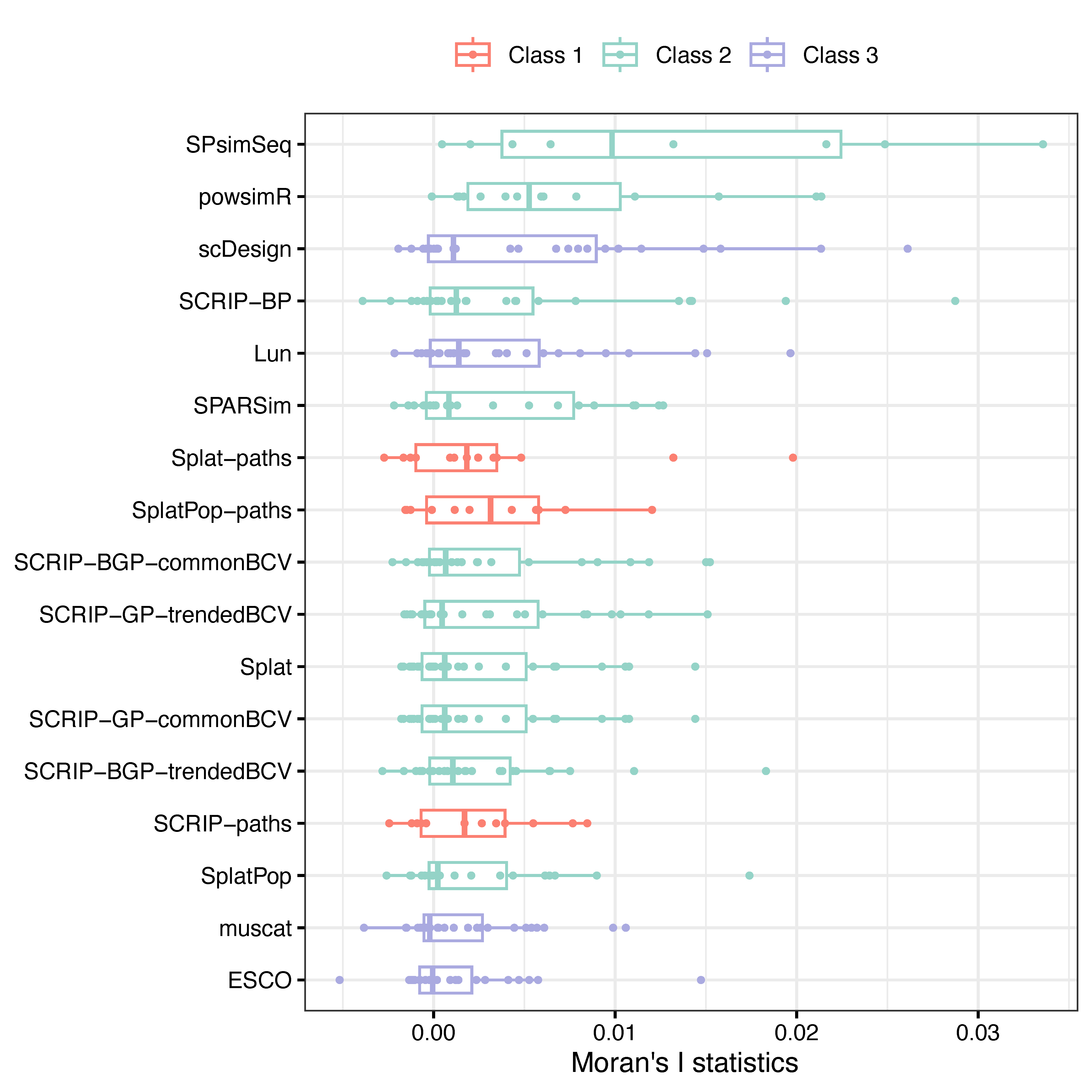


**Fig S10 Boxplots of Moran’s I statistics for SVGs generated by simulation methods.**


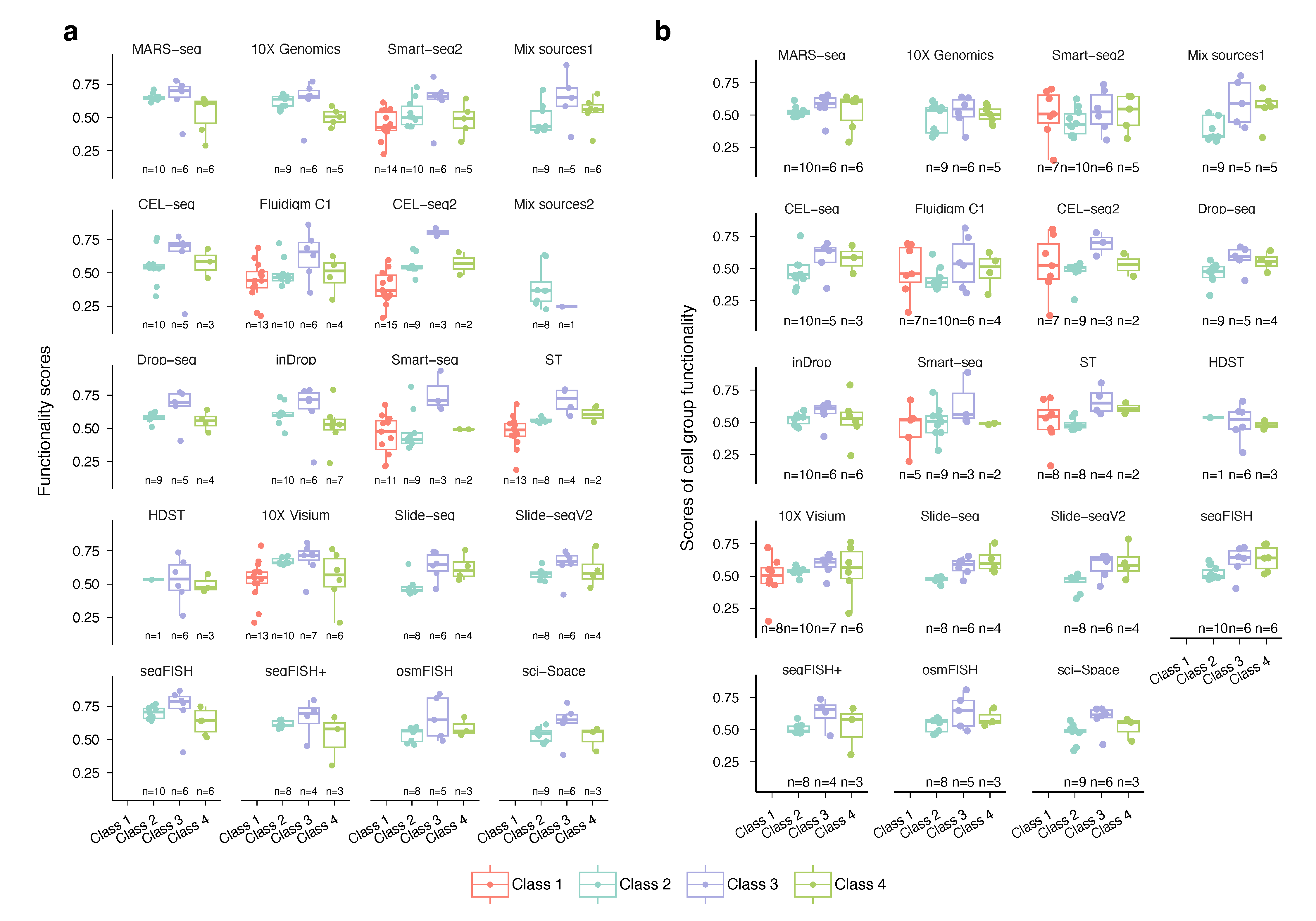


**Fig S11 Scores of overall or cell group functionality across platforms in different method classes.**

**a**, Boxplots of overall functionality scores across platforms in different method classes. **b**, Scores of simulation functionality of cell groups for each method class across different platforms. The sample size (n) is shown below the boxplots.


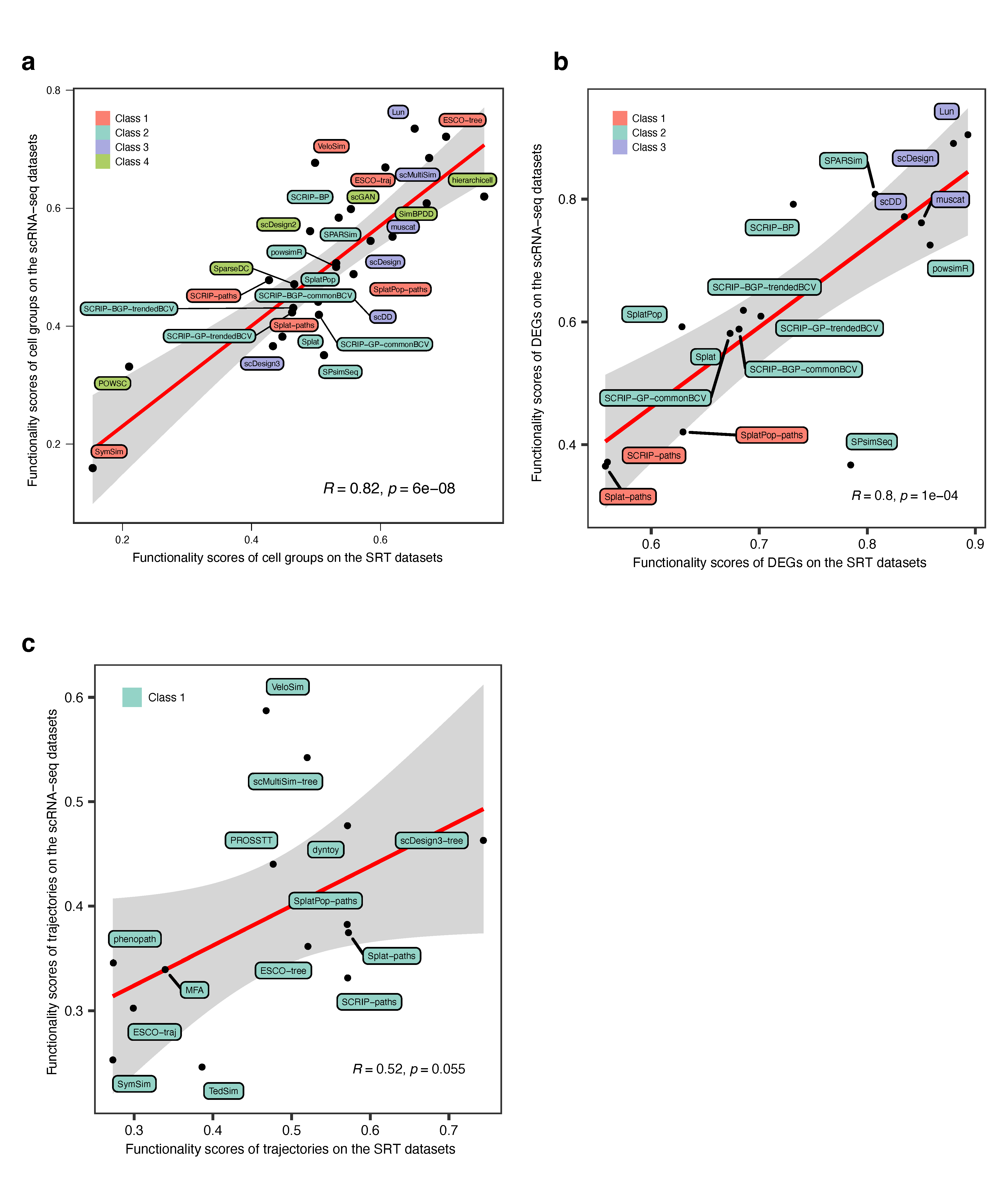


**Fig S12 Correlation between the scores on SRT and scRNA-seq datasets for different method functionalities.**

**a-c**, The Pearson correlation coefficients and *P* values were calculated between the scores on the scRNA-seq and SRT datasets for simulating cell groups, DEGs and cell trajectories. The 95% confidence interval of the fitted line was marked in grey.


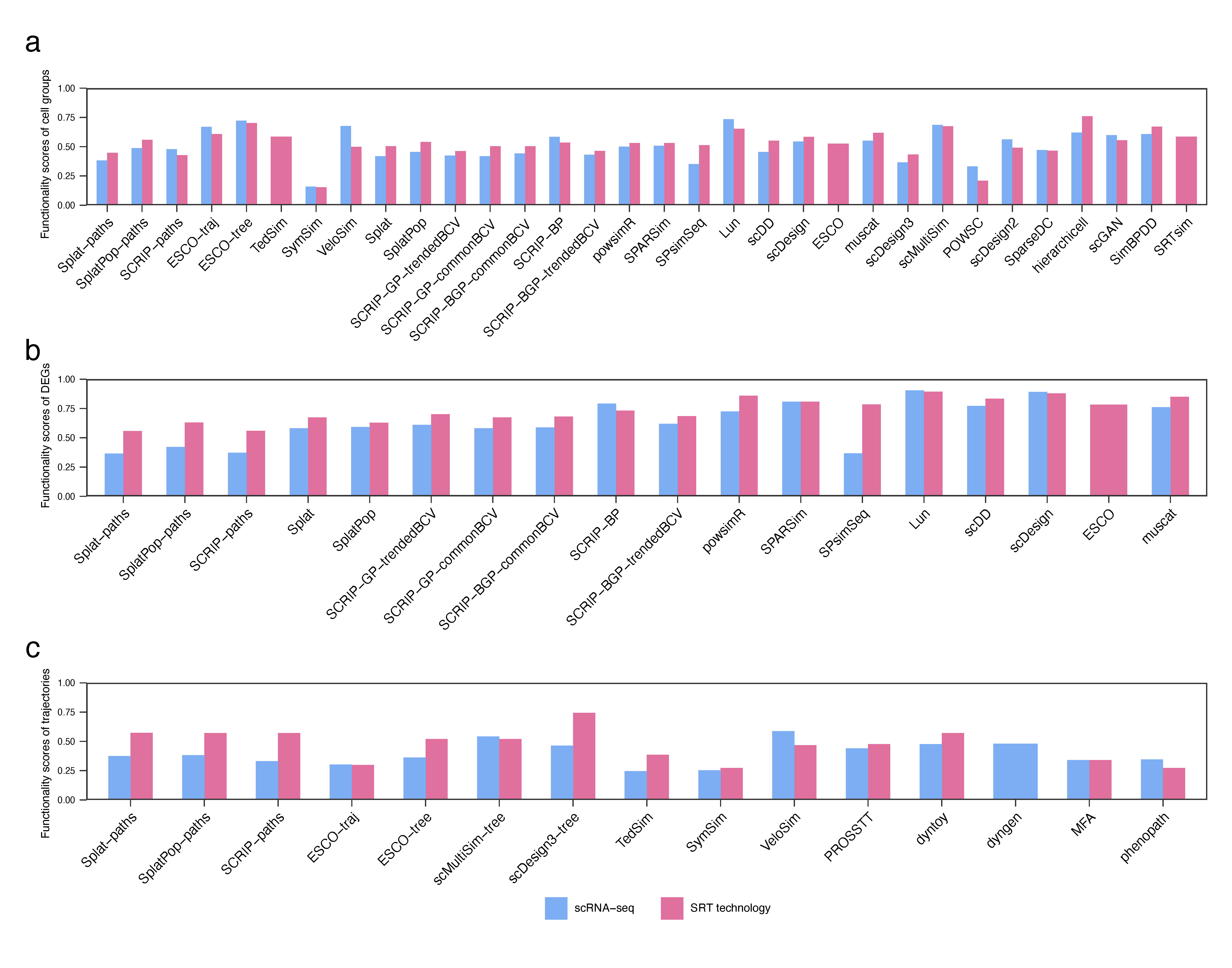


**Fig S13 Scores of three functionalities on the scRNA-seq and SRT data.**

**a-c**, Scores of the functionality of simulating cell groups, DEGs and trajectories on the scRNA-seq and SRT datasets.


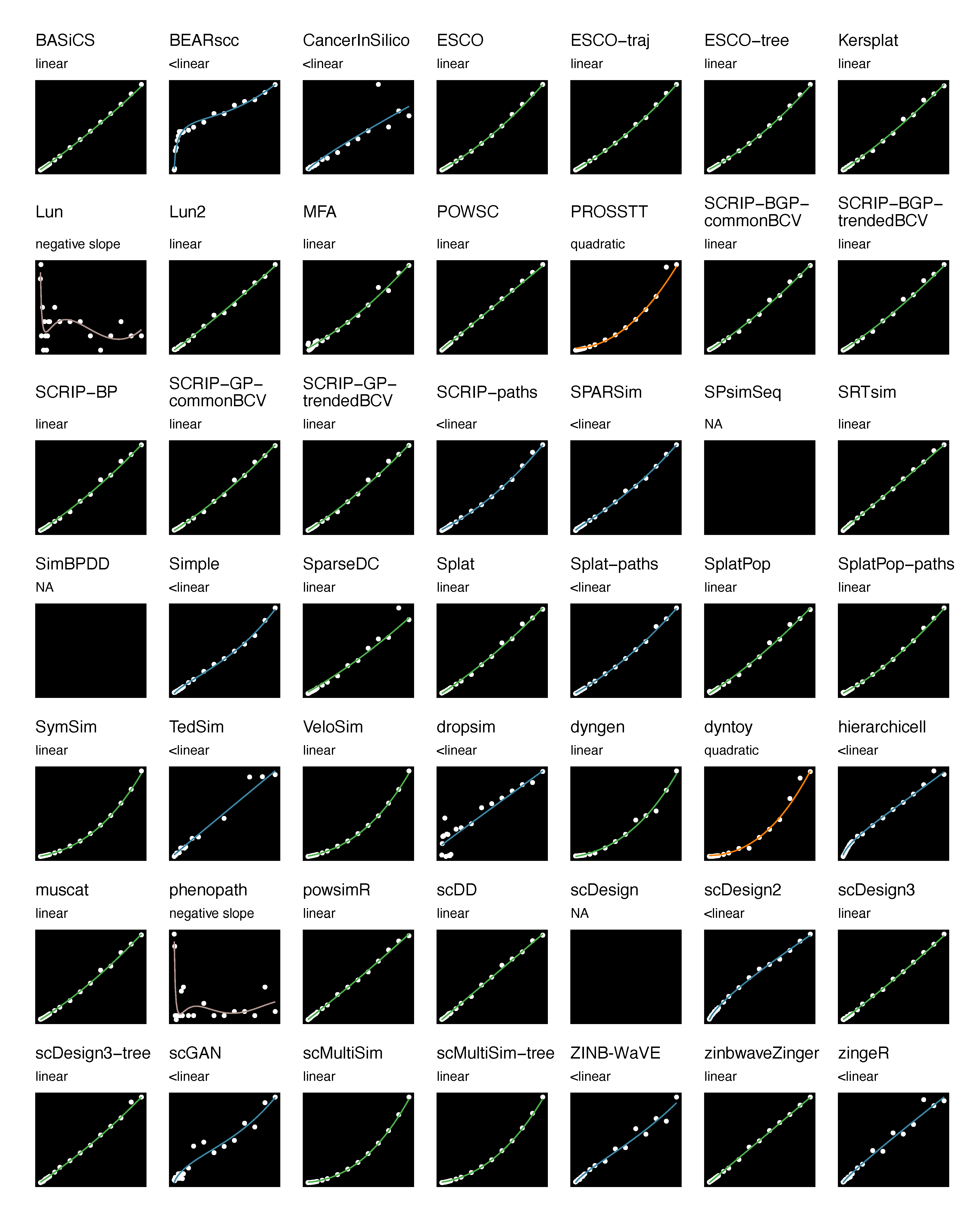


**Fig S14 Time complexity of methods with increased cell numbers in the parameter estimation step.**

**
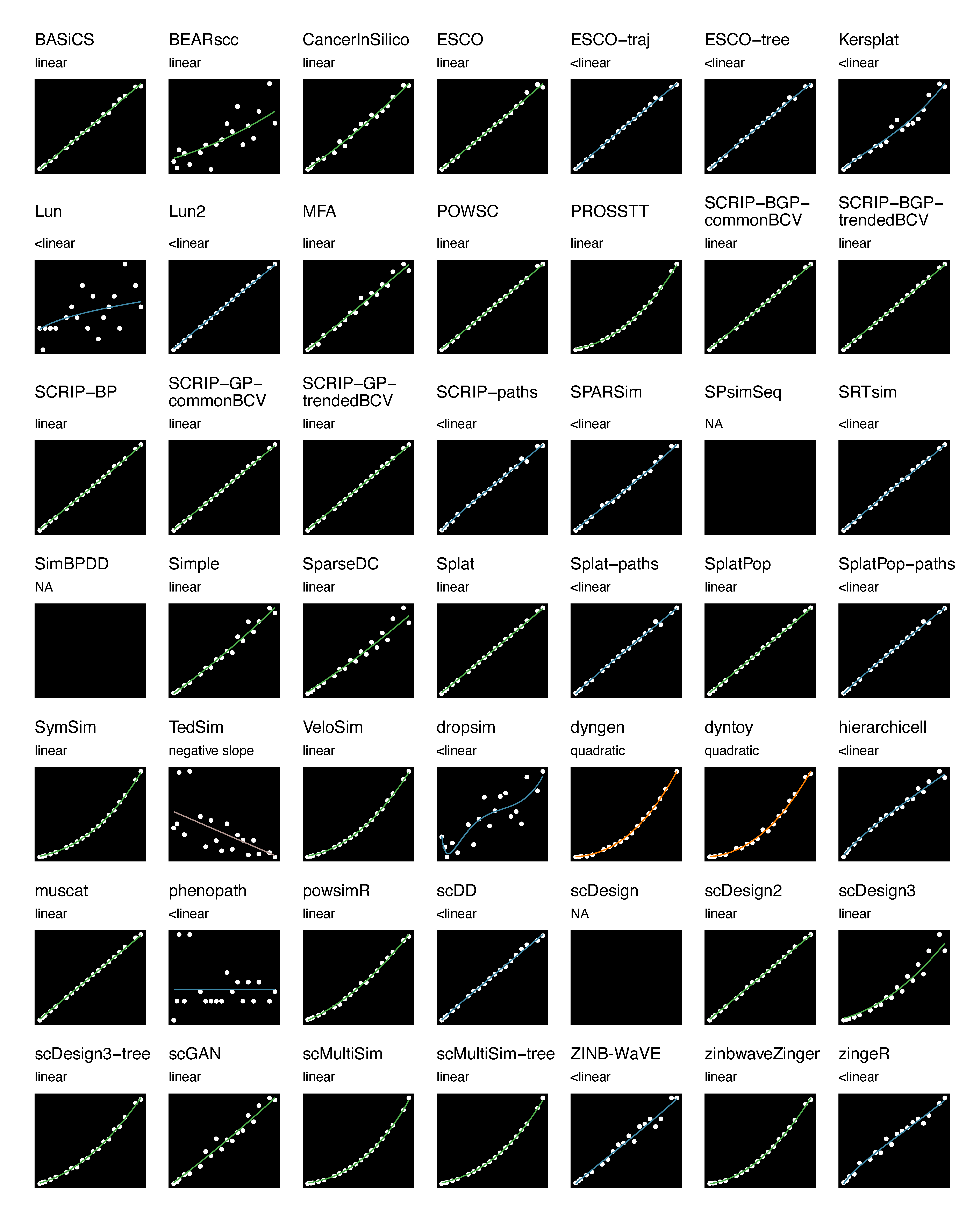
**

**Fig S15 Time complexity of methods with increased gene numbers in the parameter estimation step.**

**
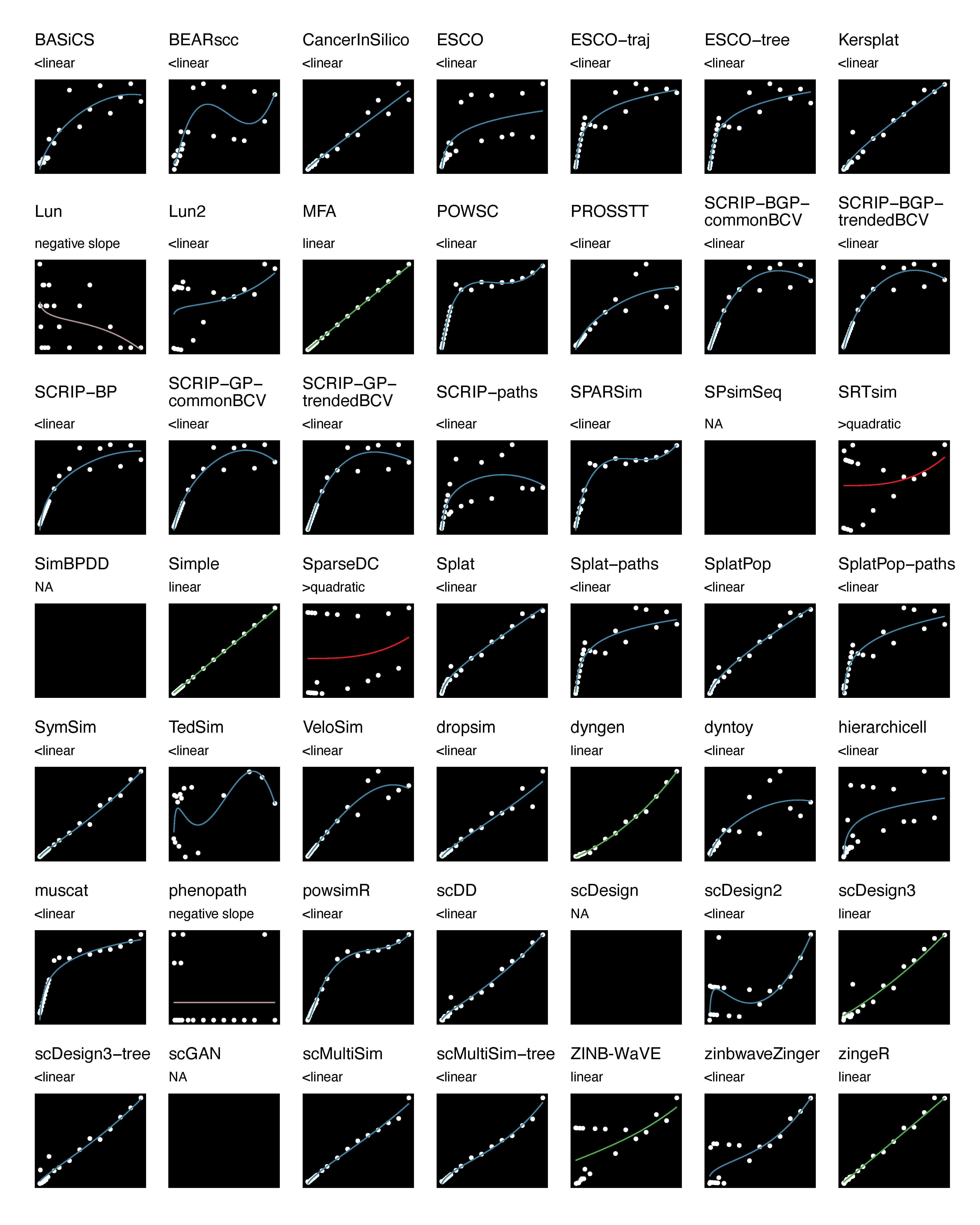
**

**Fig S16 Memory complexity of methods with increased cell numbers in the parameter estimation step.**

**
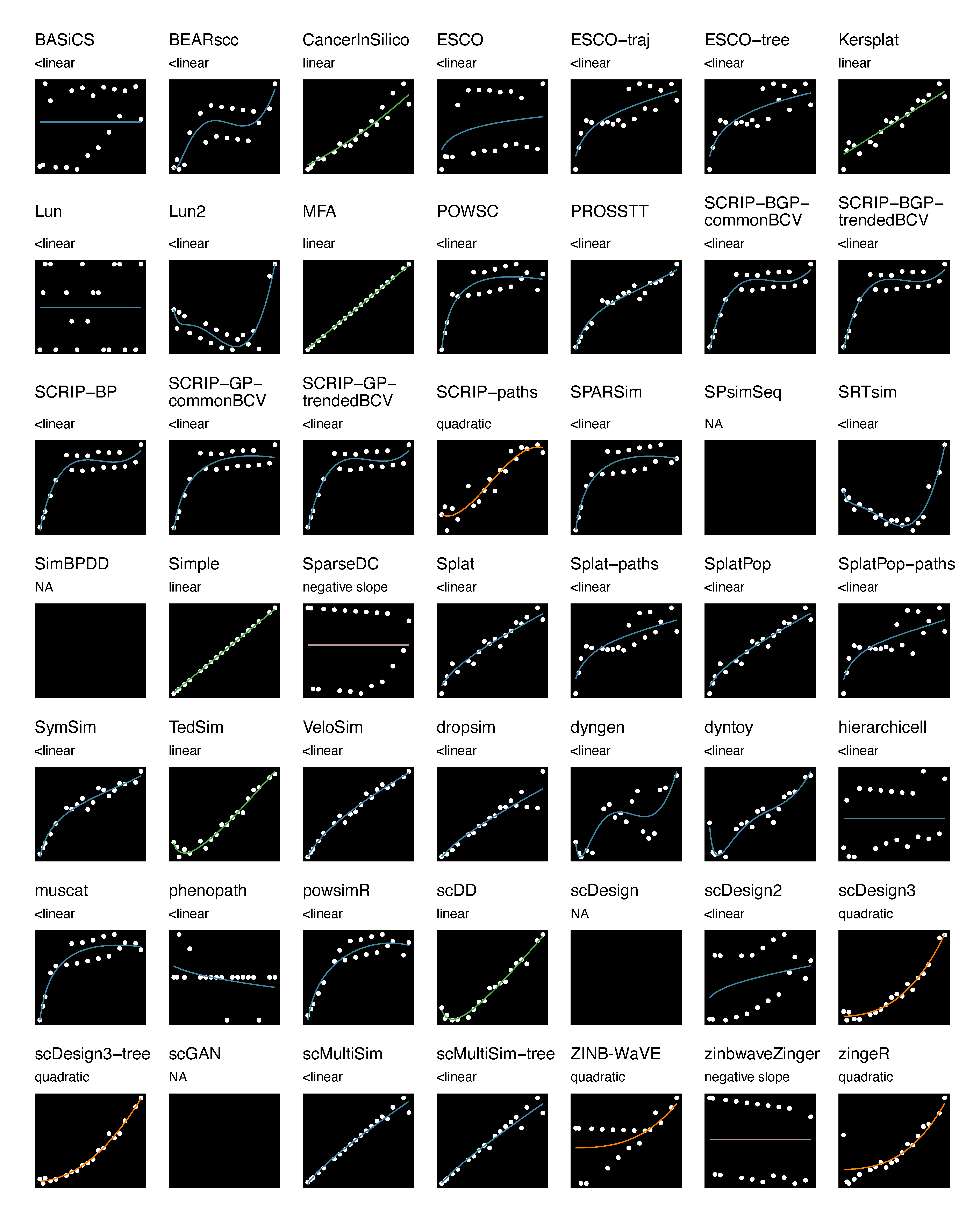
**

**Fig S17 Memory complexity of methods with increased gene numbers in the parameter estimation step.**

**
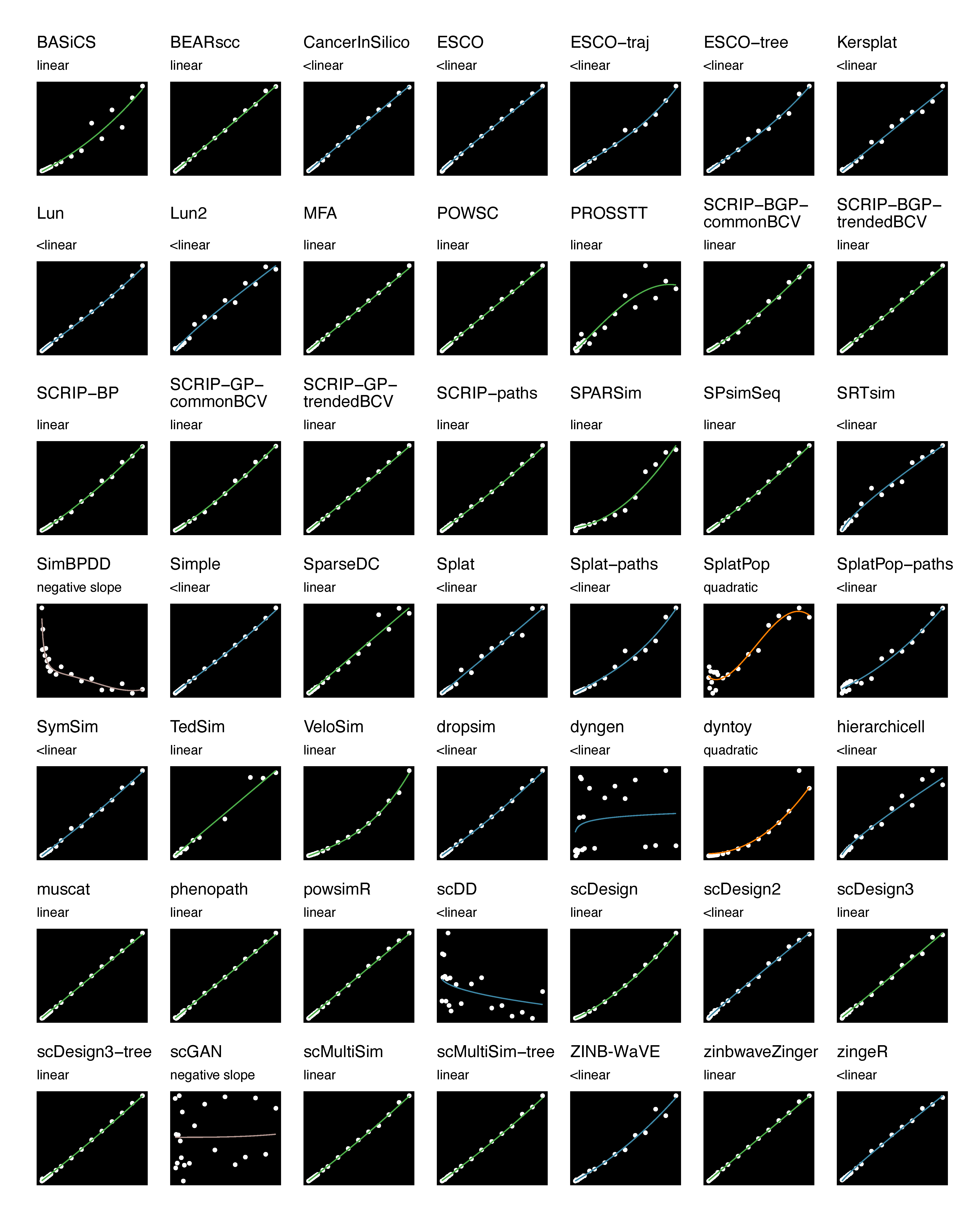
**

**Fig S18 Time complexity of methods with increased cell numbers in the data simulation step.**

**
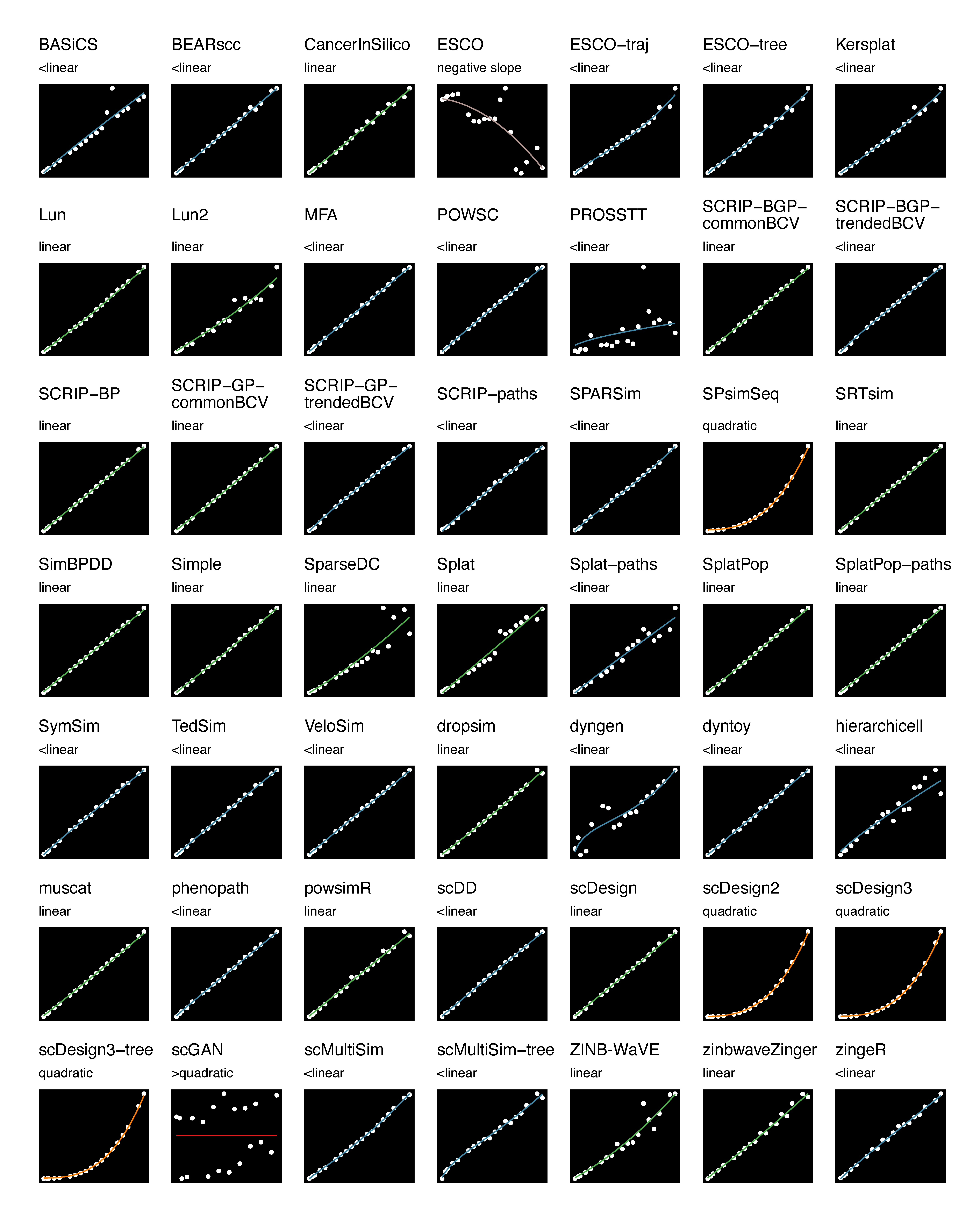
**

**Fig S19 Time complexity of methods with increased gene numbers in the data simulation step.**

**
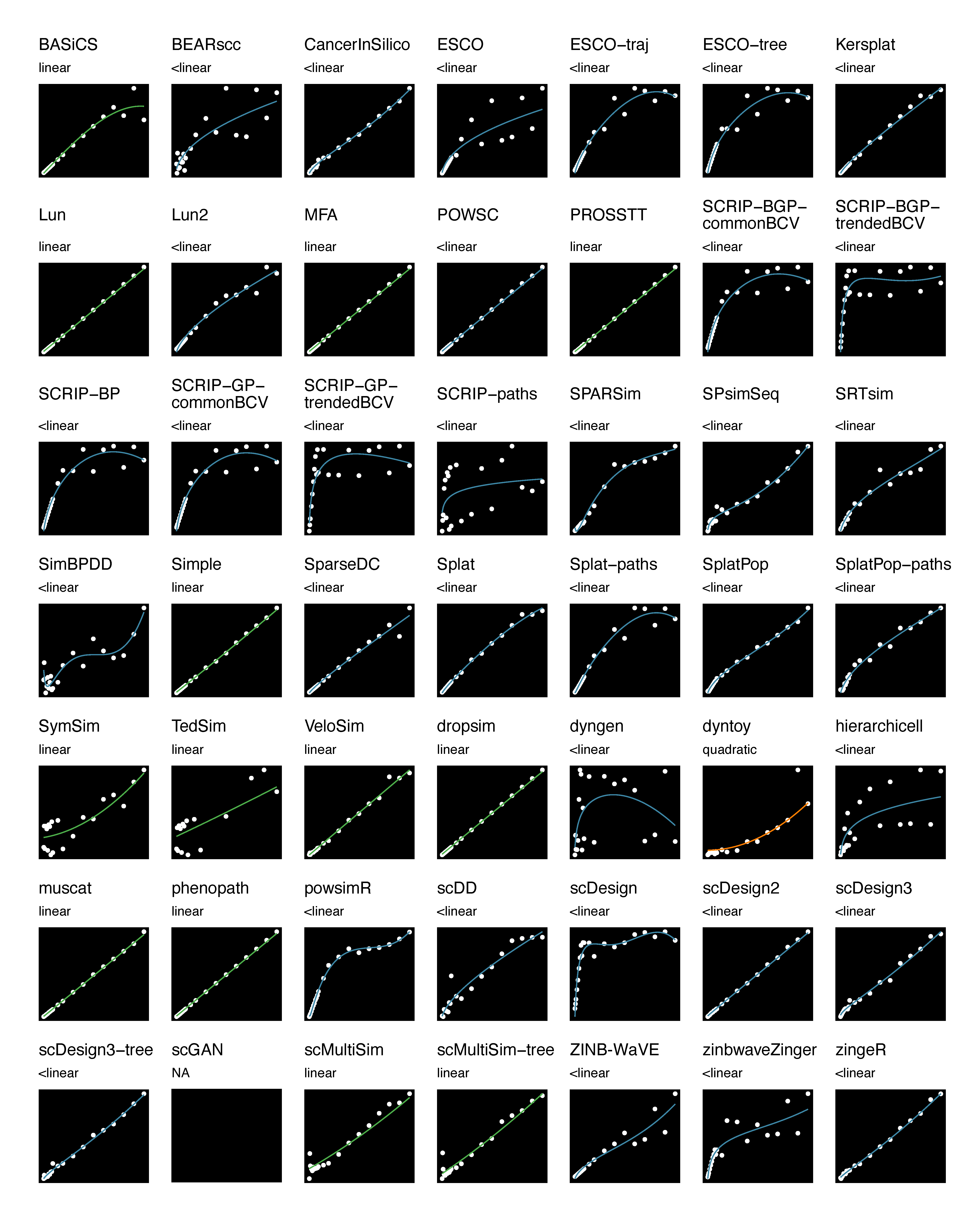
**

**Fig S20 Memory complexity of methods with increased cell numbers in the data simulation step.**

**
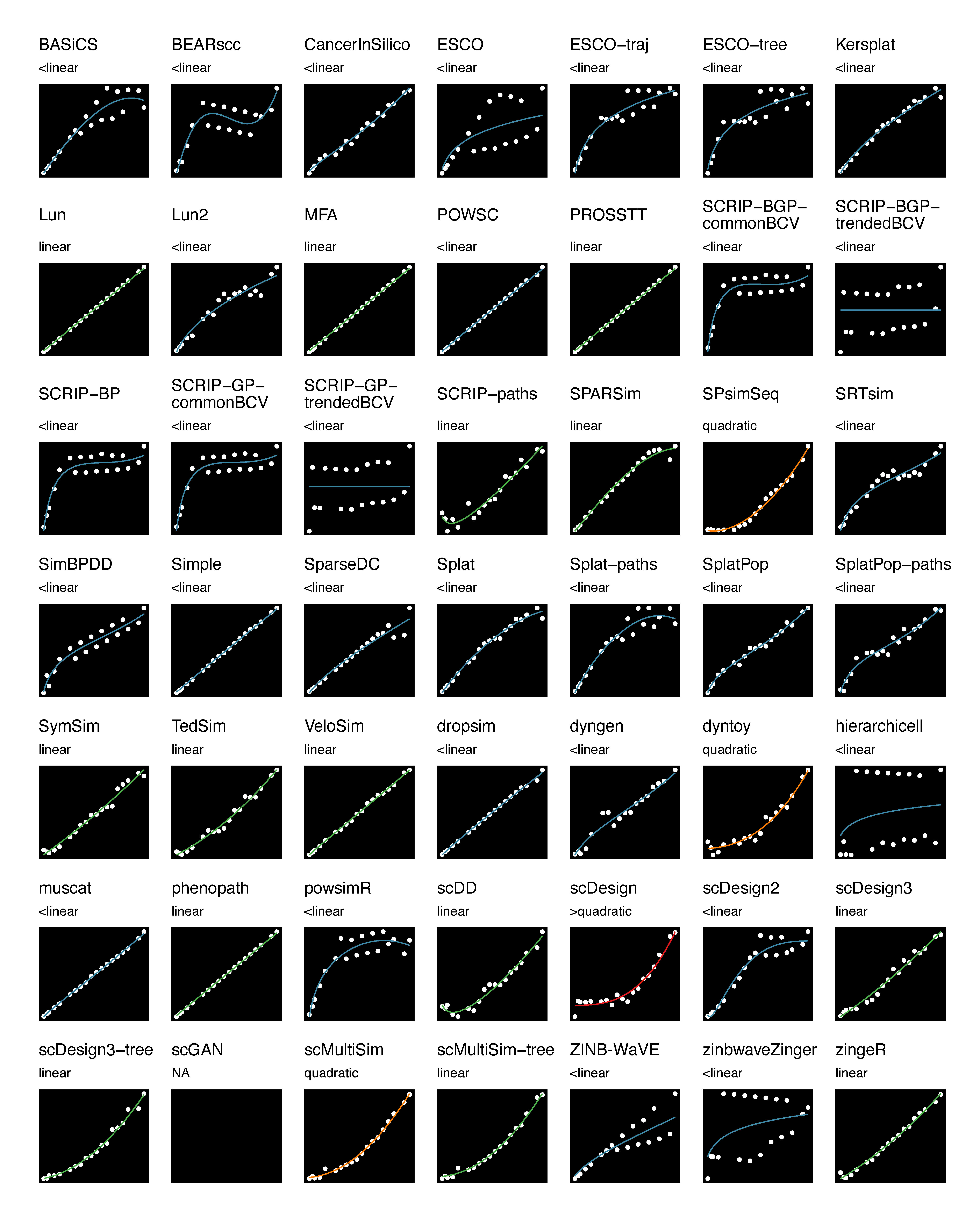
**

**Fig S21 Memory complexity of methods with increased gene numbers in the data simulation step.**

**
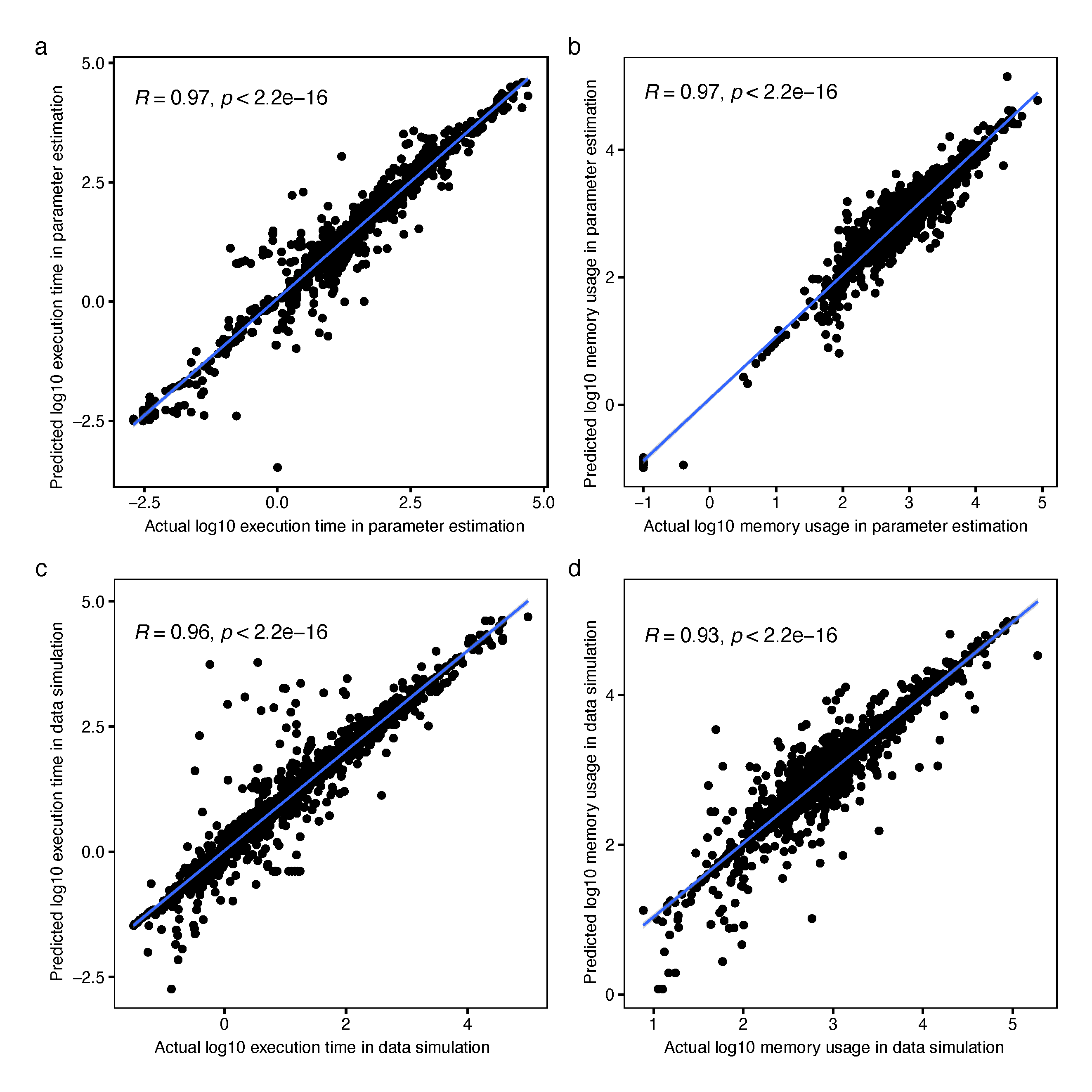
**

**Fig S22 Prediction performance of the shape constrained additive model (SCAM).**

The correlation between the actual and predicted log-normalized execution time (s) or memory usage (MB) in the parameter estimation (**a-b**) and the data simulation steps (**c-d).** The 95% confidence interval of the fitted line was marked in grey.

**
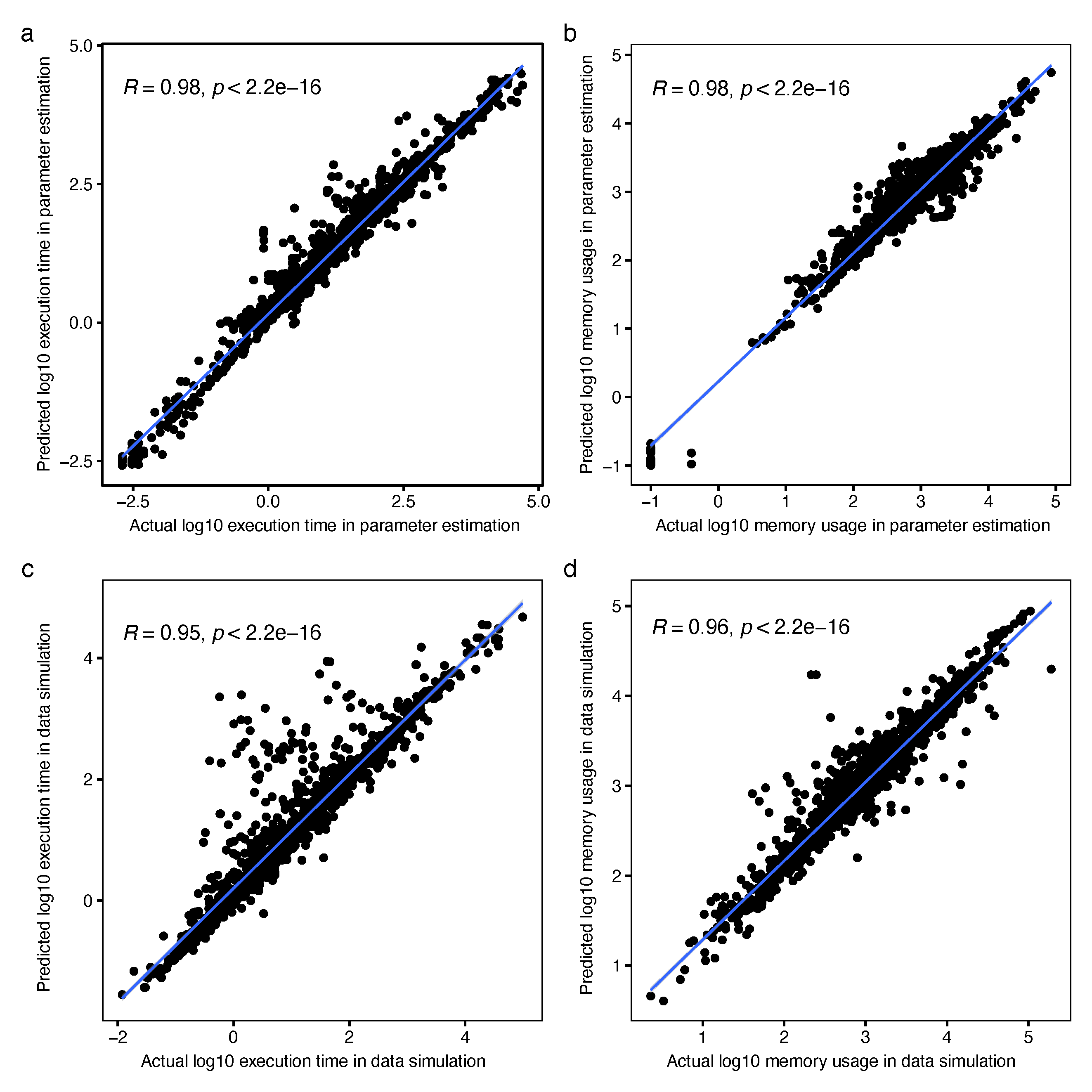
**

**Fig S23 Prediction performance of the random forest (RF) model.**

The correlation between the actual and predicted log-normalized execution time (s) or memory usage (MB) in the parameter estimation (**a-b**) and the data simulation steps (**c-d**). The 95% confidence interval of the fitted line was marked in grey.


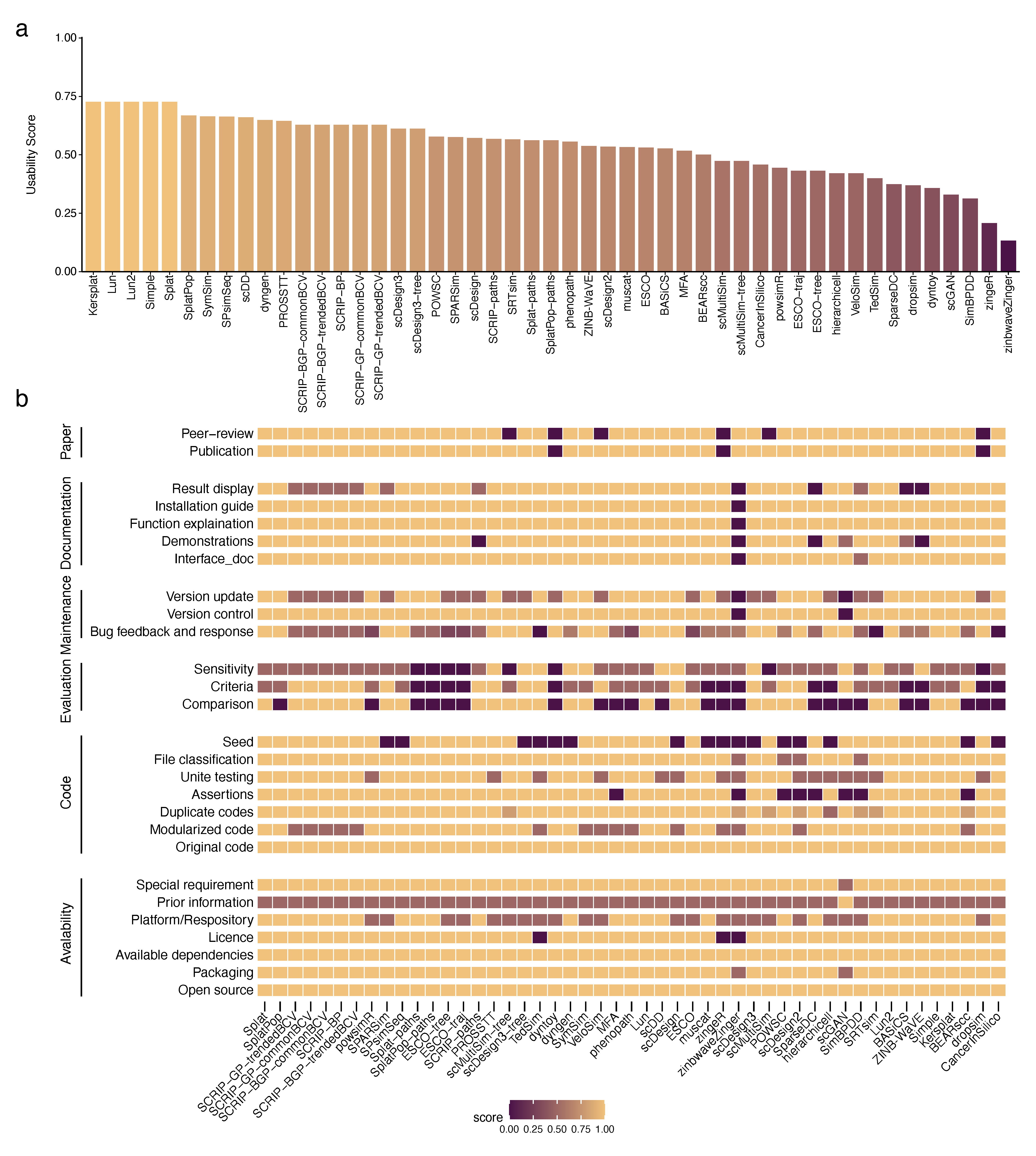


**Fig S24 The usability performance of methods.**

**a**, Usability scores of ranked methods. **b**, Heatmap of the detailed evaluation results of each usability item.


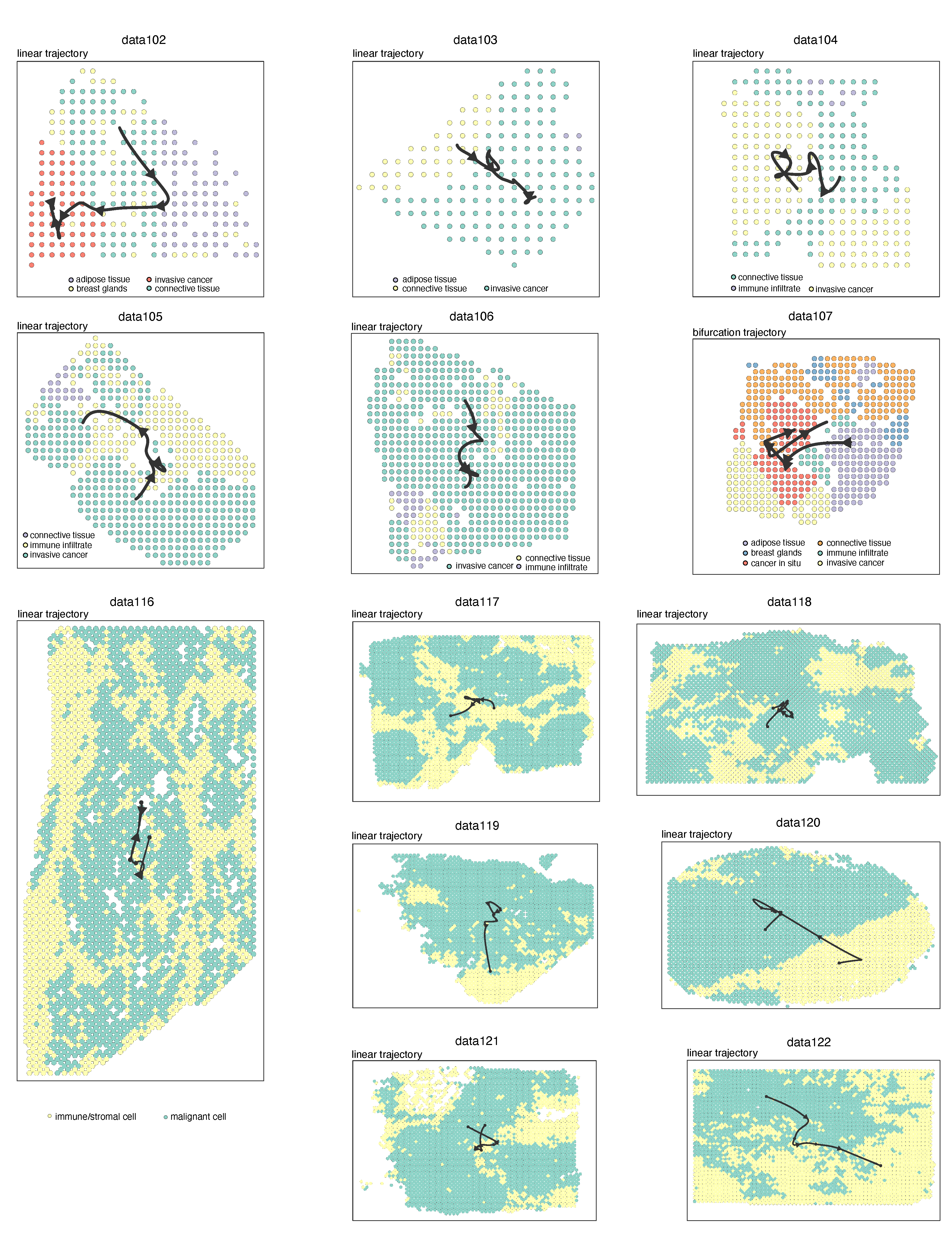


**Fig S25 Results of trajectory inference for thirteen SRT datasets.**

Trajectory inference was performed for these thirteen SRT datasets from six different cancer types. Except for the bifurcation structure inferred from data107, the other trajectories were all linear topologies. In these inference results, such as in data105, data119, data121 and data122, the malignant cells invaded and infected the regions containing healthy cells, which verified the conclusion that the process of cancer invasion and metastasis can be considered as a linear trajectory model. In addition, some results showed the transition process from healthy tissue to invasive cancer in data102, data104, data107 and data120.


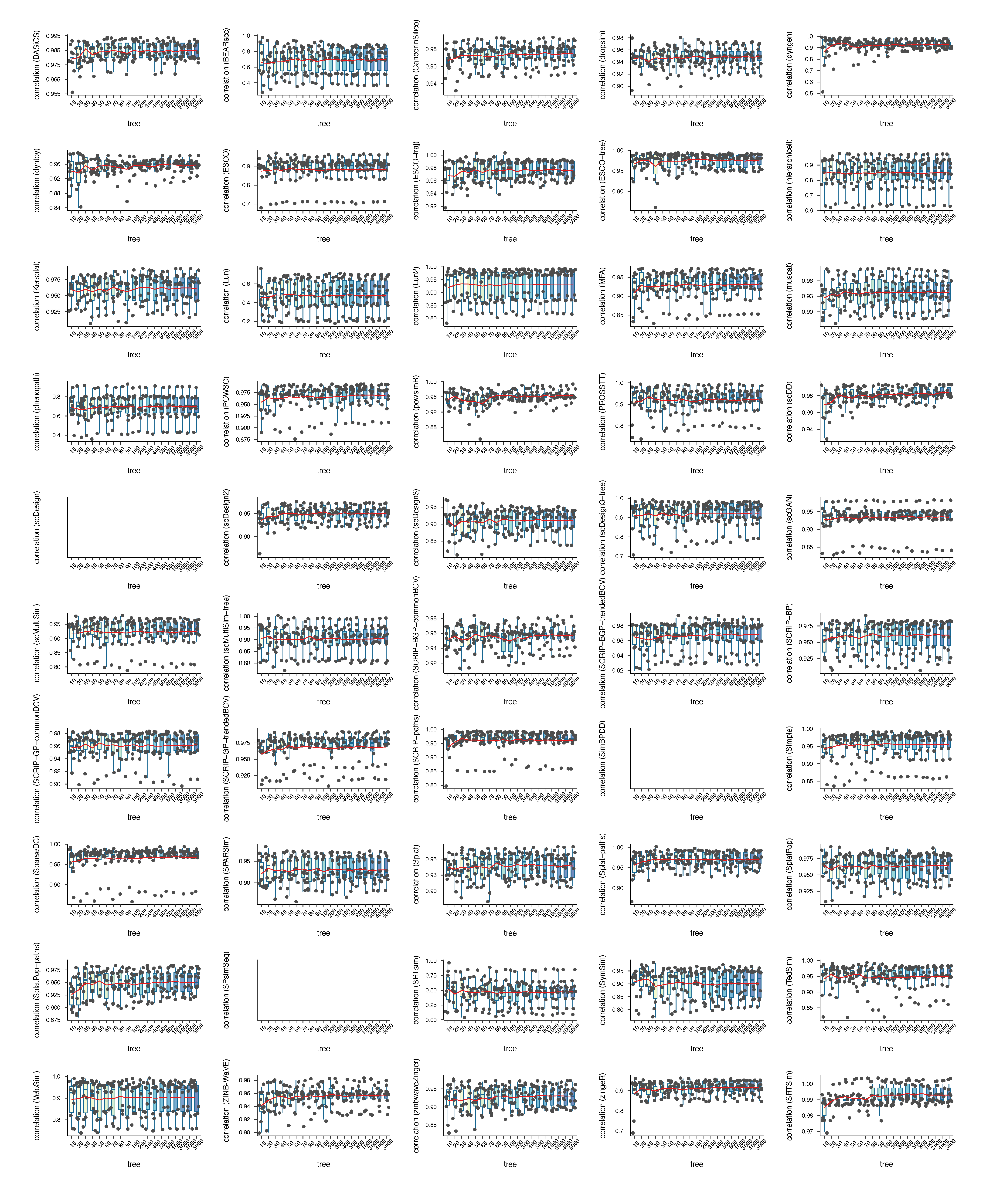


**Fig S26 Time prediction performance of RF with varying tree numbers in the parameter estimation step.**

**
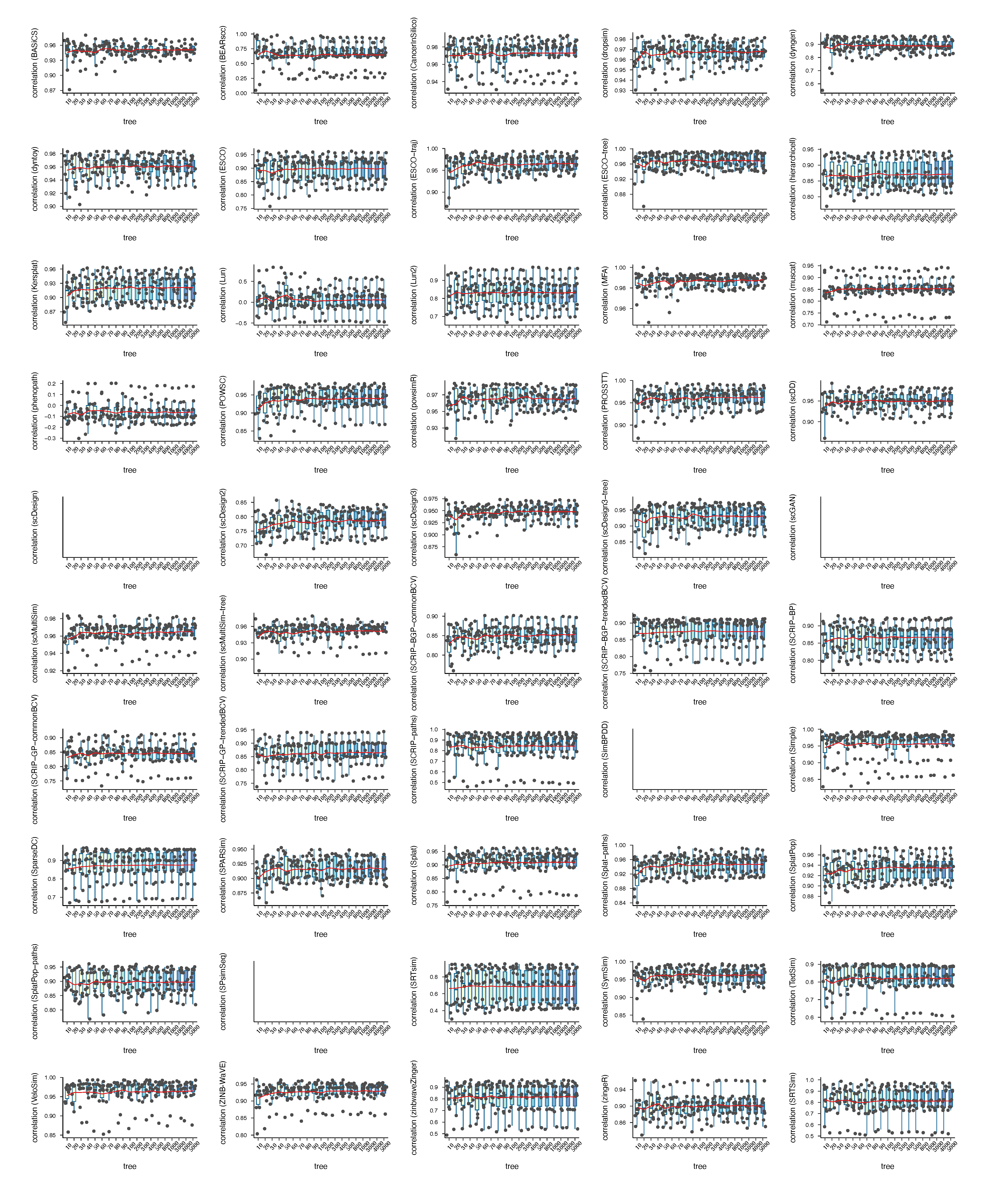
**

**Fig S27 Memory prediction performance of RF with varying tree numbers in the parameter estimation step.**

**
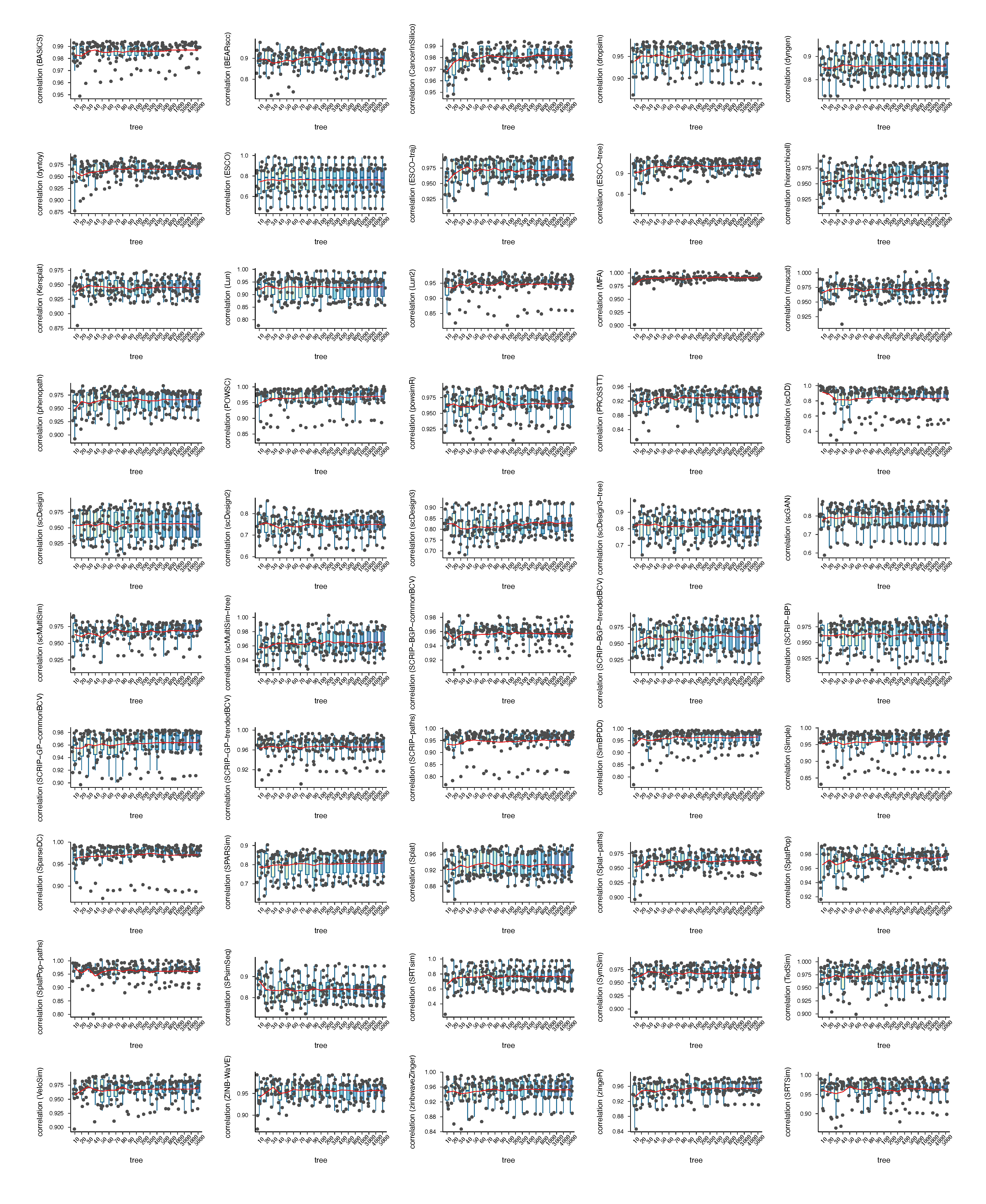
**

**Fig S28 Time prediction performance of RF with varying tree numbers in the data simulation step.**

**
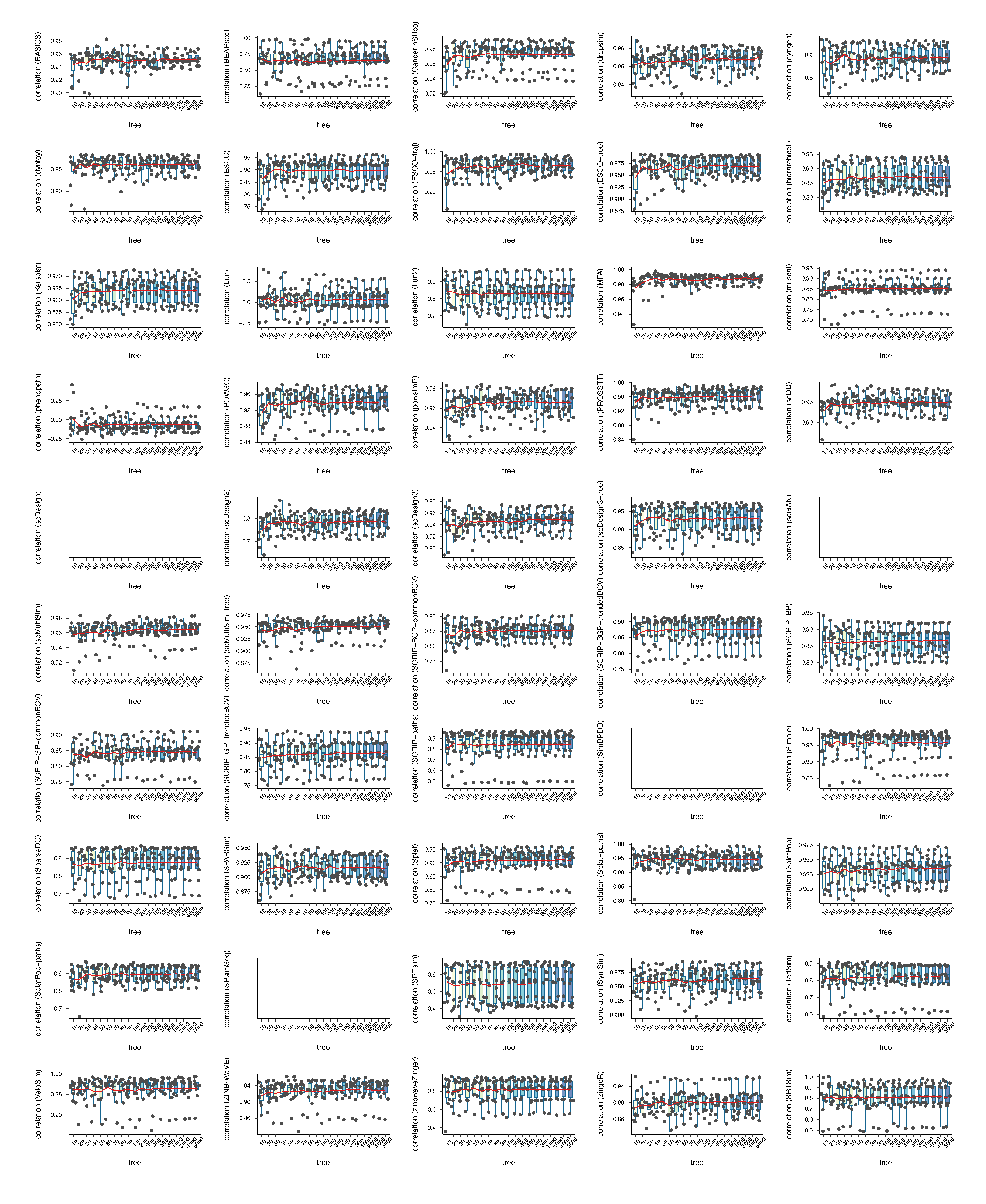
**

**Fig S29 Memory prediction performance of RF with varying tree numbers in the data simulation step.**

**
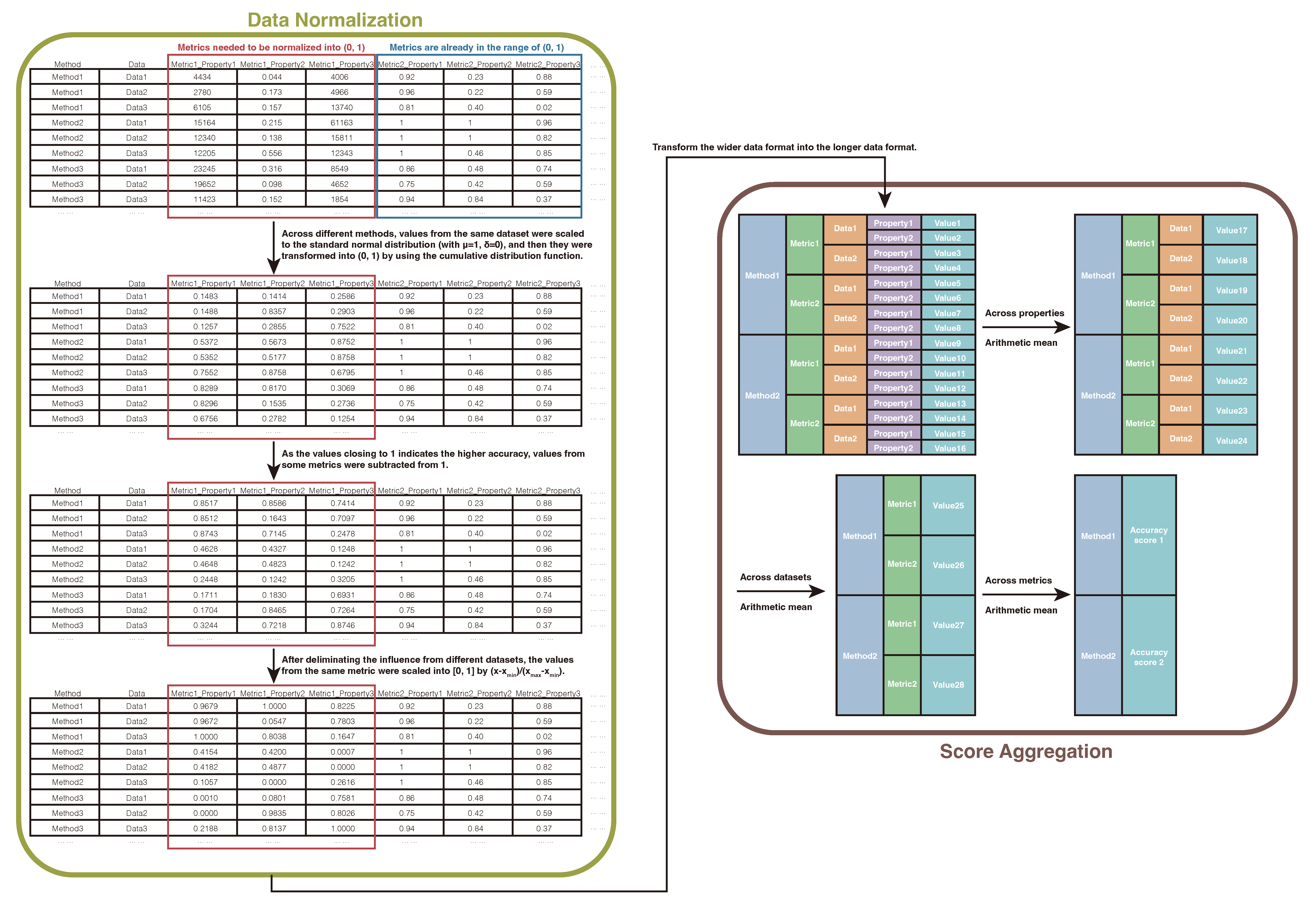
**

**Fig. S30 An example of data normalization and score aggregation process for accuracy criterion.**

# Supplementary Discussion

**Discussion S1 The negative slope of trend complexity of scalability.**

For some methods, such as ESCO, SimBPDD and TedSim, the trends of time or memory complexity showed negative slopes with increased cell or gene numbers. For instance, in Supplementary Fig. 14, Lun and phenopath have the fitted lines of execution time with negative slopes as the cell numbers increase in the parameter estimation step. Due to the automatic scaling of the value ranges, the trend variation on the y-axis is excessively amplified when all figures have the same height. Hence, we conclude that the time consumption or memory usage in the execution can not be affected by the increased cell or gene numbers for those methods. Additionally, uncontrollable factors, such as the data characteristics, may affect the running time or memory in the execution. Moreover, the results of trend complexity can not be considered as the scalability performance of methods since the trends only represent the time or memory changes in a limited range. The exact time or memory scalability performance of methods is quantified in this study and visualized in Fig. 2b and Fig. 3d.

**Discussion S2 Quadratic complexity of time with varying gene numbers for scGAN in the data simulation step.**

In Supplementary Fig. 19, we found that scGAN has quadratic complexity of execution time with gene numbers increase in the data simulation step, as the coefficient value of the superquadratic term is relatively higher than that of other terms in the generalized linear model (Methods). However, the fitted line seems to be horizontal and parallel to the x-axis. That is because the coefficient value is extremely small ($\text{1.899×}10^{\text{-28}}$), which makes the fitted line go upwards slowly.

# Supplementary Note

**1 Unifying parameter names for all methods**

Miscellaneous parameter names are not friendly to users. To popularize those data simulation methods, we unified key parameter names based on method functionalities and input data formats. The names and settings of key parameters of each method were listed in Supplementary Table 1,2. The unified parameters include those related to the dataset size, cell groups, DEGs, cell batches, and the External RNA Controls Consortium (ERCC) RNA spike-in.

*Parameters for data size*

Generally, parameters *nCells* and *nGenes* control the simulator to generate synthetic datasets with varying cell and gene numbers. However, for many methods (Splat, SplatPop, SCRIP, BASiCS, Splat-paths, SplatPop-paths and SCRIP-paths), the cell numbers in the simulated dataset were regulated by parameter *batchCells*. For example, *batchCells=5,000* indicates that the dataset contains 5,000 simulated cells. In some methods, the parameter *batchCells* also controls the generation of datasets with batch effects (see the section “*Parameters for setting cell batches*”). For method Lun, the parameter *groupCells* simultaneously controls the number of cells and cell groups. Here, *groupCells* = c(100, 200) denotes that the simulated data contains two cell groups with 100 and 200 cells, respectively. For method Lun2, the parameter with the same function is designated as *cell.plates*. Parameter settings for all methods were listed in Supplementary Table 2.

*Parameters for setting cell groupings and DEGs*

Parameters dealing with cell groups cover both the steps of parameter estimation and simulation: 1) For the parameter estimation step, *group.condition* controls the cell label information of the input data. 2) For data stimulation, optional input parameters include *nGroups* (which determines the number of cell groups), *prob.group* (which regulates the proportions of cells in different groups), *de.prob* (which governs the proportion of DEGs), and *fc.group* (which controls the fold change of DEGs). In this study, 15 methods (Splat, SplatPop, SCRIP (five modes), ESCO, scDesign2, Splat-paths, SplatPop-paths, SCRIP-paths, ESCO-traj, and ESCO-tree) utilize the *prob.group* parameter to determine the number of cell groups instead of *nGroups*. For instance, *prob.group*=c(0.3, 0.2, 0.5) represents that the simulated data contains three groups of cells and the proportions in these groups are 30%, 20% and 50%.

*Parameters for setting cell batches*

When estimating parameters, BASiCS, Lun2, scDesign3-tree and scDesign3 can use batch labels of cells by optionally setting the *batch.condition* parameter. To simulate datasets with batch effects, 12 methods (Splat, SplatPop, SCRIP-GP-trendedBCV, SCRIP-GP-commonBCV, SCRIP-BGP-commonBCV, SCRIP-BP, SCRIP-BGP-trendedBCV, powsimR, BASiCS, Splat-paths, SplatPop-paths and SCRIP-paths) require users to set either the *batchCells* or *prob.batch* parameter. For instance, setting *batchCells* = c(100,200) indicates that there are two batches of data with 100 and 200 cells, respectively. Similarly, SPARSim and SPsimSeq optionally require setting the *batch.condition* parameter to control the number of cell batches, where the length of the input vector denotes the cell number that needs to be simulated.

*Parameters for ERCC spike-in controls*

BEARscc, powsimR, SPARSim, and BASiCS allow users to optionally input the ERCC spike-in control data, including gene expression matrix, dilution factor of the ERCC spike-in control mix (regulated by *dilution.factor*), and the volume (regulated by *volume*). The number of spike-in control RNA molecules can be calculated using the following formula.

$$Y_{i}=c_{i}\cdot V\cdot D^{-1}\cdot{10}^{-18}\cdot N_{A} (i=1,2,\ldots92)$$

Where, $c_{i}$ represents the theoretical concentration of the $i$th ERCC spike-in molecule (attomoles/μL), $D$ denotes the dilution factor, $V$ stands for the volume (μL), and $N_{A}$ represents the Avogadro constant (6.02214076×10²³).

**2 Datasets with cell differentiation trajectory**

Fifteen methods (Splat-paths, SplatPop-paths, SCRIP-paths, ESCO-traj, ESCO-tree, TedSim, SymSim, VeloSim, PROSSTT, dyntoy, dyngen, MFA, phenopath, scDesign3-tree and scMultiSim-tree) were capable of simulating trajectory datasets. The detailed descriptions for data selection, processing, and the synthesis of pseudo-cells for TedSim method are provided in the subsequent sections.

*scRNA-seq datasets*

A total of 23 gold-standard scRNA-seq datasets with well-defined cell differentiation trajectories were selected from the Zenodo repository (id:1443566) [1]. Each dataset contains a gene expression matrix, cell annotation information, milestone network, and trajectory type. For more information of the standard trajectory format, please refer to dynverse (https://github.com/dynverse).

*Spatial transcriptome datasets*

The interpretation of the continuous trajectory inferred from spatial transcriptome data in space is generally more challenging compared to that from the scRNA-seq data in the low-dimensional latent coordinates. For scRNA-seq data, cells belonging to the same group tend to be clustered together. However, for spatial transcriptome data, cells with similar expression patterns may be spatially distant from each other, which can pose interference in the trajectory inference. Therefore, we preferred to construct the simplest trajectory models for SRT datasets, such as the linear topology. Furthermore, the process of cancer invasion and metastasis has been well known to follow a linear trajectory model [2, 3]. In this scenario, 13 spatial transcriptome datasets (6 datasets generated by SRT technology and 7 datasets generated by 10×Visium) from 6 cancer types were selected as the benchmarking trajectory datasets (Supplementary Table 3 and Supplementary Fig. 25).

Since seven 10×Visium datasets lack domain annotation, deconvolution analysis was performed using the SpaCET algorithm to estimate the proportion of cancer cells, immune cells and mesenchymal cells [4]. First, the analytical objects defined in SpaCET were constructed. Then, spots with less than 1 expressed gene were removed using the *SpaCET.quality.control* function, and they were then deconvolved via *SpaCET.deconvolution* function. Finally, the cell type with the highest proportion in each spot was considered as the annotation result.

*Trajectory datasets for TedSim method*

Since the TedSim method requires the input data containing $2^{x}$ cells ($x\in N$), we synthesized extra expression matrices with pseudo-cells.

1. **Calculation of the pseudo-cell number**. The pseudo-cell number was obtained by computing the value of $2^{x}$, which must be larger than the real cell number. Here, $n$ denotes the cell number in the dataset.

$$s=2^{\left\lceil log2(n) \right\rceil}-n$$

2. **Retrieval of cell group information**. If cell group information was not available, clustering was first performed using the *k*-means algorithm, and the clusters were then evaluated using the Dunn index. The evaluation helped us determine the optimal value for *k*, which represents the number of clusters in the dataset.
3. **Assignment of pseudo-cell numbers to each cell group**. We generated gene expression values for pseudo-cells according to the true gene expression profiling of each cell group. Based on cell group numbers (*G*), the desired numbers of pseudo-cells to be generated (*s*) were divided evenly and defined as $s_{1}, s_{2}, s_{3}, \ldots, s_{g}$ ($g\in G$).

**4)** **Coefficient matrix of pseudo-cells**. For each group of pseudo-cells, we defined a coefficient matrix $C_{s_{g}\times n}$. Here, $s_{g}$ represents the number of cells to be synthesized in the $g$th cell group and $n$ denotes the cell number sampled from the reference gene expression matrix. To generate this coefficient matrix, a series of coefficient values were sampled from the uniform distribution ranging from 0 to 1 and they were rescaled to ensure that the sum of coefficients in each row is 1.

5) **Sampling of real cells**. From a sub-dataset with a single group of cells, 50% of the real cells were randomly selected to form a new gene expression matrix which was defined as $R_{n\times m}$. Here, $m$ denotes the gene number.

**6) Merging two matrices**. The synthesized gene expression matrix ($E_{s_{g}\times m}$) containing pseudo-cells was obtained by multiplying the matrices of $C_{s_{g}\times n}$ and $R_{n\times m}$.

$$E_{s_{g}\times m}=C_{s_{g}\times n}R_{n\times m}$$

The gene expression values were rounded. Subsequently, the final matrix was generated by merging the synthesized and original gene expression matrices.

**3 Parameter estimation from real datasets**

**3.1 Principles of parameter estimation**

Each method requires different prior information during the parameter estimation process. To take full advantage of the diversity of the methods and datasets, and to ensure the feasibility and objectivity of our evaluation, we therefore kept the following principles:

1) Methods that do not require prior information were applied to all datasets. Otherwise, only the datasets with corresponding prior information were used;

2) Optional parameters were set as their default values to maintain consistency and avoid any bias in parameter settings;

3) Random seeds were used to ensure reproducibility and minimize any potential impact of seed initialization on the evaluation results;

4) Since three specific methods (SPsimSeq, scDesign and SimBPDD) lack an independent parameter estimation step, we did not consider their performances in parameter estimation within the scope of this study;

5) Six methods (scMultiSim-tree, SymSim, VeloSim, PROSSTT, dyntoy and dyngen) for simulating datasets with differentiation trajectories lacked independent parameter estimation functions. We thus designed procedures for handling gene expression matrices in order to obtain the standard input. This process was regarded as the parameter estimation step (see **Section 3.3**).

**3.2 Prior information required for parameter estimation**

Prior information within the datasets is crucial for parameter estimation. In this study, 26 methods are able to execute parameter estimation using prior information, including cell group labels, cell batch labels, and ERCC spike-in control (Supplementary Table 2). Parameter names have been unified in the **Section 1**. Here, we introduce the usage of prior information and considerations for some circumstances.

*Prior information of cell groups*

In this study, 22 methods require the labels of cell groups as prior information. Users can input a numeric vector containing cell group labels through the *group.condition* parameter. Notably, scDD only simulates the data containing two cell groups. Therefore, it can only accept the prior information of two cell groups.

*Prior information of batches*

Lun2, BASiCS, scDesign3-tree and scDesign3 can retrieve prior information with cell batch labels using the *batch.condition* parameter which represents a numeric vector containing cell batch labels. For instance, *batch.condition*=c(1,1,1,2,2,2) means that the dataset has two batches of cells and each batch contains 3 cells.

*Prior information of ERCC spike-in control*

The information of ERCC spike-in control is required for BEARscc, but optional for powsimR, SPARSim, and BASiCS. Detailed prior information of ERCC spike-in control has been described in **Section 1**. Notably, all 92 spike-in control RNAs should be correctly named in the gene expression matrix.

**3.3 Parameter estimation for the datasets with cell trajectory**

Parameter estimation based on reference datasets is a necessary step before data simulation. Except for SPsimSeq, scDesign, and SimBPDD (in which the steps of parameter estimation and simulation were merged), the remaining 46 methods require an independent parameter estimation step. Of these, 40 methods have independent functions for parameter estimation, while six methods (scMultiSim-tree, SymSim, VeloSim, PROSSTT, dyntoy and dyngen) for simulating datasets with cell differentiation trajectories do not have functions for parameter estimation.

To prepare the input data or files required for the simulation, raw reference datasets were processed as follows:

1) Reference datasets were used for building Seurat objects and natural-log transformation (*scale.factor=10,000*).

2) Top 2,000 highly variable genes were selected using variance stabilizing transformation (*vst*), and then the datasets were scaled.

3) In each cell group, gene expression values were averaged. If the information of cell groups was not available, the downstream analysis such as the unsupervised clustering was performed using the *FindClusters* function.

4) Hierarchical clustering was used to construct the relationships between cell groups (using *clust* function, *method= 'ward.D'*).

5) The results of hierarchical clustering were transformed into the required data format for each method. Besides, the processing steps for special methods are as follows:

PROSSTT can simulate scRNA-seq data in the light of information of cell differentiation trajectories. Simulation of datasets with complex topologies can be achieved if corresponding input information is provided. Based on the results of hierarchical clustering, the *hclust* format was converted into the Newick format using the *hc2Newick* function implemented in the *ctc* R package.

scMultiSim-tree, SymSim and VeloSim require a tree file with *phylo* format. We therefore converted the clustering results from Newick format to the *phylo* format using the *read.tree* function implemented in the *ape* R package.

Dyntoy and Dyngen require cell group labels as prior information. If they are not available in the reference dataset, the *k*-means algorithm was applied to cluster the cells. The optimal value of *k*, which represents the number of clusters, was determined according to the Dunn index. Subsequently, the trajectory inference was conducted using Slingshot [5]. The predicted topology model and milestone network of the trajectory were used as input for dyngen and dyntoy, respectively.

**4 Data simulation**

**4.1 Principles of the data simulation process**

In order to showcase the advantages of each method, we obeyed the following principles during the simulation process:

1) Prior to the simulation, we summarised the information of cell groups, DEGs and cell batches for input (see **Section 4.2**), so that the information of cells or genes within the real and corresponding simulated data was consistent;

2) Optional parameters were set as their default values;

3) Random seeds were set to ensure reproducibility and facilitate comparisons between different simulated datasets;

4) We allowed methods to simulate datasets by taking the corresponding prior information as input when it was available. It is not only beneficial to display the capabilities of the methods, but also to make full use of the prior information within the real data;

5) As ZingeR and zinbwaveZinger can not return the labels of cell groups in the simulated data, we did not evaluate their performances in simulating cell groups and DEGs.

**4.2 Preparing essential information for simulation input**

To improve the similarity between the simulated data and the real data, we conducted a thorough analysis of the information related to the cell groups, DEGs, and cell batches for the real data. This additional information was then used as input for the optional parameters in the data simulation step.

*Input information related to cell groups*

The parameters related to cell groups consist of *nGroups*, *prob.group* and *group.condition*. The usage of *group.condition* parameter has been illustrated in **Section 1**. The *nGroups* parameter determines the number of cell groups in the simulated data, which can be customed by the command in R: *length(unique(group.condition))* when scDesign and muscat were used. The *prob.group* parameter denotes a numeric vector of the cell proportions in different groups, which can be set by the command in R: *table*(*group.condition*)/*length*(*group.condition*). When using a method that incorporates the above parameters, *nGroups* or *prob.group* is introduced as an additional parameter for input.

*Input information related to differential expression genes (DEGs)*

The *de.prob* parameter governs the proportion of DEGs in the real data. First, we paired cell groups within the real data, and then performed differential expression analysis (DEA) for genes by employing the edgeRQLFDetRate (edgeR QLF model including the cellular detection rate) algorithm. Afterward, we united all the DEGs detected from different pairs of cell groups, which formed a set $U$. The DEG proportion of the real data was calculated using the formula:

$$\text{proportion of DEGs}=\frac{N}{G}$$

where $N$ is the element number in set $U$ and $G$ is the total gene number.

*Input information related to batches*

Parameters dealing with batch effects are *batch.condition* and *batchCells*. The *batch.condition* parameter was illustrated in **Section 1**. The *batchCells* parameter requires a numeric vector containing the cell number in each batch, which can be obtained by the command in R: *unname(table(batch.condition)).* The vector length represents the expected number of cell batches.

**4.3 Output of the simulated data**

In our simulation pipeline, users can easily define their preferred output formats, including *list*, *Seurat object*, *SingleCellExperiment*, and *h5ad*. Regardless of the selected formats, the simulation results incorporate the gene expression matrix, cell and gene information, as well as the detected execution time and memory usage throughout the simulation process.

1) In the gene expression matrices, genes are in rows and cells are in columns. They are reversed in *h5ad* files.

2) Cell annotation information mainly consists of the labels of cell groups or batches. If a particular method requires setting parameters for cell groups, the simulated results will return the corresponding group labels. However, it is noted that zingeR and zinbwaveZinger do not provide any cell group information due to their inherent limitations.

3) For the annotation information of genes, Splat, SplatPop, Lun, Lun2, ESCO and SCRIP are able to return DEGs with fold changes between pairwise cell groups. However, methods such as scDesign, muscat, and SPARSim can only return the DEG labels across all group pairs, instead of those between any of the two cell groups.

**5 Evaluation metrics**

**5.1 Metrics for accuracy criterion**

1. Median absolute deviation (MAD). MAD is defined as:

$$MAD=median(\left| P_{i}^{r}-P_{i}^{s} \right|)$$

Here, $P^{r}$ and $P^{s}$ denote the numeric vectors containing one-dimensional data property values of the real ($r$) and the simulated ($s$) gene expression data, respectively. $i$ is the $i$th value in the vector.

1. Kolmogorov-Smirnov distance (KS distance). The KS distance is defined as the maximum vertical distance between two cumulative distributions of data properties derived from the real and the simulated datasets.

$$D_{n,n}=\max_{x} \left| R\left( x \right)-S(x) \right| (n>1)$$

where $x$ is the data property value of the real or the simulated data. $R(x)$ and $S(x)$ respectively denote the empirical cumulative distribution function of the data properties derived from the real and the simulated data. $n$ represents the vector length of the data property.

1. Mean absolute error (MAE). MAE is the average of all absolute errors and is defined as:

$$MAE=\frac{1}{n}\sum_{i=1}^{n} \left| P_{i}^{r}-P_{i}^{s} \right|$$

where $P^{r}$ and $P^{s}$ present the numeric vectors of data properties summarized from the real and simulated data. $i$ is the $i$th value in the vector of data property and $n$ is the length of the numeric vector.

1. Root mean square error (RMSE). RMSE is the standard deviation of the residuals calculated by subtracting the data property value of the simulated data from that of the real data. $P^{r}$, $P^{s}$ and $i$ have been defined in the description of MAE.

$$RMSE=\sqrt{\frac{1}{n}\sum_{i=1}^{n} {(P_{i}^{r}-P_{i}^{s})}^{2}}$$

2. Overlapping index (OV) [6]. The distribution-free overlapping index was used to quantify the similarity between two empirical distributions of data properties. Let $r=\left( r_{1},\ldots,r_{i},\ldots,r_{n} \right)$ and $s=\left( s_{1},\ldots,s_{i},\ldots,s_{n} \right)$ be the data property sets of the real data and the simulated data, respetively. Then the density $f_{R}$ and $f_{S}$ were estimated using the kernel density estimator:

$$\hat{f}_{R}\left( x \right)=\frac{1}{n}\sum_{i=1}^{n} \mathcal{K}\left( \frac{r-r_{i}}{\beta} \right)$$

$$\hat{f}_{S}\left( x \right)=\frac{1}{n}\sum_{i=1}^{n} \mathcal{K}\left( \frac{s-s_{i}}{\beta} \right)$$

where $\mathcal{K}$ is the Gaussian Kernel function, $\beta$ is the bandwidth parameter. The overlapping index is defined as follows:

$$\hat{\eta}\left( R,S \right)=\int_{\mathbb{R}^{n}} \min\left[ \hat{f}_{R}(x),\hat{f}_{S}\left( x \right) \right]dx$$

$\hat{f}_{R}$ and $\hat{f}_{S}$ respectively denote the results of Gaussian Kernel density estimation of one-dimensional data property derived from the real and the simulated data. The overlapping index was computed using the freely-available R package *overlapping*.

1. Bhattacharyya distance (BH distance). BH distance is an effective metric to measure the overlapping degree between two probability distributions. For continuous probability distributions, the Bhattacharyya distance is defined as:

$$d(P_{r}, P_{s})=-\ln(\int_{\chi} \sqrt{p_{r}\left( x \right)p_{s}\left( x \right)}dx)$$

where $P$ represents the probability distributions on the one-dimensional space $\chi$; $p_{r}(x)$ and $p_{s}(x)$ are the probability density functions of data properties calculated from the real and the simulated data.

1. Multi-dimensional Kolmogorov-Smirnov test (multiKS). To assess the similarity between two-dimensional data properties, the multi-dimensional Kolmogorov-Smirnov test was performed as described by Fasano and Franceschini [7].

Let $R=\left( r_{1},\ldots,r_{i},\ldots,r_{n} \right)$, $S=\left( s_{1},\ldots,s_{i},\ldots,s_{n} \right)$, where $R$ and $S$ denote the data property sets of the real and the simulated data. For a given point $x\in\mathbb{R}^{d}$, the $i$th open orthant with origin $x$ is defined as:

$$\mathcal{O}_{i}(x)=\left\{ \left. x^{'}\in\mathbb{R}^{d} \right|e_{ij}\left( x_{j}-x_{j}^{'} \right)>0, j=1,\ldots,d \right\}$$

where $d$ denotes the $d$-dimensional vector, $e_{i}\in\left\{ -1,1 \right\}^{d}$ is a length $d$ combination of $\pm1$. There are $2^{d}$ combinations, indicating the $2^{d}$ orthants that divide $\mathbb{R}^{d}$. The indicator function is

$$I_{j}\left( x | y \right)=\left\{ \begin{aligned} 1,x\in\mathcal{O}_{j}(y) \\ 0,x\notin\mathcal{O}_{j}(y) \end{aligned} \right.$$

the distance $D(p)$ is defined as:

$$D\left( p \right)=\max_{1\leq j\leq2^{d}} \left| \frac{1}{n}\sum_{k=1}^{n} \left[ I_{j}\left( r_{k} | p \right)-I_{j}\left( s_{k} | p \right) \right] \right|$$

In this test, the distance $D$ is maximized separately for each sample and generates two statistics:

$$D_{1}=\max_{1\leq i\leq n} D(r_{i})$$

$$D_{2}=\max_{1\leq i\leq n} D(s_{i})$$

The final result was the arithmetic mean of $D_{1}$ and $D_{2}$. The two-sample multi-dimensional Kolmogorov-Smirnov test was computed using the *fasano.franceschini.test* R package [8].

1. Kernel density. The discrepancy of two-dimensional data property was measured using the kernel density function based on the global two-sample comparison test. The $T$ statistic is calculated by the integrated squared error (ISE):

$$T=\int\left[ f_{r}\left( x \right)-f_{s}\left( x \right) \right]^{2}dx$$

Where $f_{r}$ and $f_{s}$ denote the density function of two-dimensional data property derived from the real and the simulated data, respectively. Then, the $Z$ statistic was calculated based on the $T$ statistic to measure the dissimilarity between the two data properties.

**5.2 Metrics for assessment of simulated cell groups/spatial domains**

1. Clustering deviation index (CDI). CDI is a robust and accurate internal measure for evaluating scRNA-seq data clustering [9]. The top 500 genes based on working dispersion scores (WDS) were selected. Then, we calculated the penalized negative log-likelihood based on the cell label set. If the CDI score is small, the simulated label set is close to the true label set. The likelihood function is:

$$\hat{\mathcal{l}}\left( \mu_{gk}^{\left( b \right)},\phi_{gk}^{\left( b \right)}:g\in\left[ G \right],k\in\left[ K \right] \right)=\sum_{g\in\left[ G \right]} \sum_{c\in S_{b}} \log\left\{ F\left( X_{gc}|s_{c}\mu_{gL_{c}},\phi_{gL_{c}} \right) \right\}$$

where $F$ is the negative binomal (NB) probability mass function of $X_{gc}$ which denotes the counts of gene $g$ in cell $c$ (cell type $k$). In addition, $X_{gc}|(L_{0,c}=k)\sim NB(s_{c}\mu_{gk},\phi_{gk})$, where $s_{c}$ is a scalar factor to adjust cell library size. $\mu_{gk}$ and $\phi_{gk}$ are the mean and dispersion parameters of gene $g$ in cell type $k$, respectively. The penalized negative log-likelihood function is:

$$\tilde{\mathcal{l}}=-2\hat{\mathcal{l}}+c_{pen}\cdot d (d=\sum_{g\in\left[ G \right]}{\sum_{k\in\left[ K \right]}d}_{gk})$$

where $d$ is the degree of freedom of the model and $c_{pen}$ is the penalty scalar: $c_{pen}=2$ for AIC (Akaike Information Criterion) and $c_{pen}=\log(N)$ for BIC (Bayesian Information Criterion). $N$ is the length of the cell label set.

1. ROUGE. ROUGE is an entropy-based metric, enabling accurate, sensitive and robust assessment of cell cluster purity by quantifying the randomness of gene expression in cells [10]. A cell population with higher purity will receive a value close to 1 and reversely close to 0. Before calculating the metric, the low-abundance genes and low-quality cells were removed with the cutoff of *min.cell=10* and *min.genes=10*. ROUGE score was calculated individually for a single population and then all scores were aggregated into a final weighted score:

$$\text{weighted ROUGE}=\sum_{i=1}^{k} P_{i}\cdot S_{i}$$

where $k$ is the group number. $P_{i}$ denotes the proportion of cell numbers in the $i$ group to all cells, ranging from 0 to 1. $S_{i}$ is the ROUGE score of the $i$ group.

1. Average silhouette width (ASW). The silhouette width is a common internal metric to measure the extent of within-cluster distances of a cell and between-cluster distances of that cell to the closest cluster [11]. The average silhouette width ranges from -1 to 1. The ASW is defined as follows.

$$\text{ASW}=n^{-1}\sum_{i=1}^{n} \frac{b\left( i \right)-a\left( i \right)}{max\left( a\left( i \right),b\left( i \right) \right)}$$

where $a\left( i \right)$ is the average dissimilarity between $i$ and all other cells of the cluster to which $i$ belongs. If $i$ is the only observation in a cell cluster, the silhouette width of $i$ is 0. For all other clusters $C\neq A$, let $d(i,C)$ be the average dissimilarity of $i$ to all cells in $C$. There is a cluster $B$ whose dissimilarity $d(i,B)$ is minimal and defined as $b\left( i \right)=\min_{C} d(i,C)$. Lastly, the average silhouette width was calculated.

1. Dunn index. Dunn index is a clustering validity index calculating the ratio of the smallest distance between cells that are not in the same cluster to the largest distance of cells in the same cluster. The index is defined as:

$$D=\frac{\min_{1\leq i\leq j\leq n}d\left( i,j \right)}{\max_{1\leq k\leq n}d^{'}\left( k \right)}$$

where $d\left( i,j \right)$ denotes the distances between the two clusters $i$ and $j$. $d^{'}\left( k \right)$ denotes the intra-cluster distance of the $k$ cluster. Dunn Index ranges from 0 to infinite. The higher the index, the better the clustering.

1. Connectivity. The Connectivity metric is designed to measure the connectedness degree of the partitioning clusters, which can be also used to assess the clustering performance [12].

$$C=\sum_{i=1}^{N} \sum_{j=1}^{L} x_{i,nn_{i\left( j \right)}}$$

where $L$ is the parameter giving the number of nearest neighbors (Here $L$ was set as 10). $N$ is the total cell number in a dataset. $nn_{i\left( j \right)}$ is defined as the $j$th nearest neighbor of cell $i$. $x_{i,nn_{i\left( j \right)}}$ is zero if $i$ and $j$ are in the same cluster and it is $1/j$ otherwise. The connectivity score ranges from 0 to infinite. The lower score shows a better cell grouping.

1. Davies-Bouldin index (DB Index). DB index measures the similarity between intra-cluster dispersion and separation of inter-clusters. DBI is computed as follows:

$$DBI=N^{-1}\sum_{i=1}^{N} \max_{j\neq i} \frac{\bar{S}_{i}+\bar{S}_{j}}{\left\| \omega_{i}-\omega_{j} \right\|_{2}}$$

where $\bar{S}_{i}$ and $\bar{S}_{j}$ denote the average distance of cells to the centroid of the cluster $i$ and $j$. $\left\| \omega_{i}-\omega_{j} \right\|_{2}$ presents the Euclidean distance between the centroids of cluster $i$ and $j$. The smaller the DBI is, the better the clustering result.

**5.3 Metrics for assessment of simulated cell batches**

1. Cell-specific mixing score (CMS). CMS is an innovative measurement to determine whether the batch-wise distances in a KNN graph follow the same distribution using the Andersion-Darling test [13]. If the batch effect exists, the distance distributions from multiple batches of cells will show significant differences. Ideally, the method yields a lower CMS, which indicates it can simulate more reliable data with different batches.
2. Local inverse Simpson’s index (LISI). To address the problems of the metric sensitivity to the local distances between cells and the interpretation, LISI [14] builds Gaussian kernel-based distributions of neighborhoods and uses the inverse Simpson’s Index ($1/\sum_{b=1}^{B} P_{b}$). $P_{b}$ is the probability of a certain batch for each cell within the $k$-nearest neighborhoods, and $B$ is the total batch number. LISI scores represent the expected cell numbers to be sampled before two are drawn from the same batch. A lower LISI score indicates stronger batch effects. Otherwise, a LISI score close to the batch number suggests cells are well-mixed across different batches. We computed the arithmetic mean of all cells’ LISI scores as the final result.
3. Mixing metric. The mixing metric utilizes the median position of the $k$-th cell (k_pos_) in the KNN graphs from each batch to determine the degree of batch effects [15]. The lower the score, the better mixed the cells from different batches are. By default, we set *k_pos_=5* in the study.
4. Shannon entropy. Shannon entropy can quantify the randomness and complexity of scRNA-seq datasets containing cells from different sources of batches [16]. Shannon entropy is calculated using the relative abundance of batch $i$ as the probability in each KNN graph.

$$\text{entropy}=-\frac{1}{n}\sum_{i=1}^{n} p\left( x_{i} \right)\cdot\log\left( p\left( x_{i} \right) \right)$$

where $p\left( x_{i} \right)$ is the probability that cells come from batch $i$, and $n$ is the batch number in the data.

1. k-nearest neighbor batch effect test (kBET). kBET assesses the batch mixing by comparing the batch distribution within KNNs of a cell with the global one [17]. kBET uses a $\chi^{2}$-based test for random neighborhoods of a fixed size to determine whether they are well-mixed, and return an overall rejection rate. We used the top 50 PCs prior to the KNN search and set $k=\sqrt{n}$ (n is the cell number in the data) for the construction of KNN graphs [18]. The rejection rate of kBET close to 1 indicates that the cells from different batches are poorly mixed, exhibiting the powerful ability of the methods to simulate cell batches.
2. Average silhouette width for batch (ASW batch). Beyond being applied to evaluate clustering performance (see the section *Metrics for assessing cell group simulation*), ASW can also be used for batch evaluation, but it has different meanings. Here, the batch effect is more pronounced when the AWS closes to 1 or -1. The batch effect is not detectable when the ASW closes to 0.
3. Principal component regression score (PCR score). As the batch effects contribute to the variance of principal components (PCs), a certain batch covariate correlates significantly with some of the PCs. The PCR score represents the total contribution of the batch variable to the variance of a dataset [17]. It is determined by the sum of all weighted coefficients of $R^{2}$:

$$\text{PCR}=\sum_{i=1}^{N} \mathrm{var}\left( C|{PC}_{i} \right)\cdot R^{2}\left( {PC}_{i}|B \right)$$

where $R^{2}\left( {PC}_{i}|B \right)$ is calculated through a linear regression of the batch variable $B$ with each principal component ${PC}_{i}$. It is weighted by the $\mathrm{var}\left( C|{PC}_{i} \right)$ which is the variance explained by the $i$th principal component in the data matrix $C$.

**5.4 Metrics for assessment of simulated trajectories**

1. Hamming–Ipsen–Mikhailov (HIM) metric. The HIM metric is a distance function quantifying the similarity between two milestone networks, which linearly combines a local Hamming distance and a global Ipsen-Mikhailov distance. Although the Hamming distance allows the comparison of the matching edges in the adjacent matrices, the trajectory topology is ignored. Meanwhile, the Ipsen-Mikhailov distance can only show the similarity of degree distributions. HIM overcomes the drawbacks of local and global metrics, making it well to reflect the similarity of the structure and degree of the two trajectories.
2. F1 score for branches (F1_branches_) and F1 score for milestones (F1_milestones_). F1_branches_ score is used to measure the accuracy of branch assignment of cells, while F1_milestones_ is for comparing the arrangements of cells belonging to different milestones. To calculate F1_branches_ and F1_milestones_, cells were mapped to their nearest branches or milestones. The F1 score was calculated as follows:

Step1: the similarity of pairwise clusters was computed by Jaccard similarity:

$$Jaccard(c,c^{'})=\frac{\left| c\cap c^{'} \right|}{\left| c\cup c^{'} \right|}$$

Step 2: the Recovery and Relevance scores were defined as:

$$Recovery=\frac{1}{\left| C \right|}\sum_{c\in C} {max}_{c^{'}\in C^{'}}Jaccard(c,c^{'})$$

$$Relevance=\frac{1}{\left| C \right|}\sum_{c^{'}\in C^{'}} {max}_{c\in C}Jaccard(c,c^{'})$$

Step3: the F1 score was defined as the harmonic mean of Recovery and Relevance scores:

$$F1=\frac{2}{\frac{1}{\mathrm{Recovery}}+\frac{1}{\mathrm{Relevance}}}$$

1. Correlation between geodesic distances (Cor_dist_). If the position of a cell is the same in both the real and simulated trajectory, its relative distances to all other cells in the two trajectories should also be the same. Here, Cor_dist_ measures the correlation of the two relative distances derived from the real and simulated trajectory, reflecting the consistency of cells’ locations within the trajectories. However, the matching relationship was unclear and undefined if one cell is from the real data while another is from the simulated data. To solve this problem, the Hungarian algorithm implemented in *RcppHungarian* R package was adopted. Before matching cells, PCA was performed on the real and simulated data, and then the top 50 principal components were used for Harmony algorithm to eliminate the variations between datasets. Pearson correlation coefficients between the real and simulated cells were calculated. Subsequently, cell matching was performed using the Hungarian algorithm.

After ordering the simulated cells by the decreasingly sorted correlation values, we chose the top 5%-50% cells (step by 5%) as waypoints to calculate the Cor_dist_. Thus, 10 sets of waypoints generated 10 Cor_dist_ values. In the end, the arithmetic mean of 10 values was calculated.

**References**

1. Saelens W, Cannoodt R, Todorov H, Saeys Y. A comparison of single-cell trajectory inference methods. Nat Biotechnol. 2019;37**:**547-54.

2. Ren Y, Huang Z, Zhou L, Xiao P, Song J, He P, Xie C, Zhou R, Li M, Dong X, et al. Spatial transcriptomics reveals niche-specific enrichment and vulnerabilities of radial glial stem-like cells in malignant gliomas. Nat Commun. 2023;14**:**1028.

3. Shang L, Zhou X. Spatially aware dimension reduction for spatial transcriptomics. Nat Commun. 2022;13**:**7203.

4. Ru B, Huang J, Zhang Y, Aldape K, Jiang P. Estimation of cell lineages in tumors from spatial transcriptomics data. Nat Commun. 2023;14**:**568.

5. Street K, Risso D, Fletcher RB, Das D, Ngai J, Yosef N, Purdom E, Dudoit S. Slingshot: cell lineage and pseudotime inference for single-cell transcriptomics. BMC Genomics. 2018;19**:**477.

6. Pastore M, Calcagni A. Measuring distribution similarities between samples: a distribution-free overlapping index. Front Psychol. 2019;10**:**1089.

7. Fasano G, Franceschini A. A multidimensional version of the Kolmogorov–Smirnov test. Mon Not R Astron Soc. 1987;225**:**155-70.

8. Puritz C, Ness-Cohn E, Braun R. fasano. franceschini. test: an implementation of a multidimensional KS test in R. arXiv. 2021**:**2106.10539.

9. Fang J, Chan C, Owzar K, Wang L, Qin D, Li QJ, Xie J. Clustering Deviation Index (CDI): a robust and accurate internal measure for evaluating scRNA-seq data clustering. Genome Biol. 2022;23**:**269.

10. Liu B, Li C, Li Z, Wang D, Ren X, Zhang Z. An entropy-based metric for assessing the purity of single cell populations. Nat Commun. 2020;11**:**3155.

11. Rousseeuw PJ. Silhouettes: a graphical aid to the interpretation and validation of cluster analysis. J Comput Appl Math. 1987;20**:**53-65.

12. Brock G, Pihur V, Datta S, Datta S. clValid: an R package for cluster validation. J Stat Softw. 2008;25**:**1-22.

13. Lütge A, Zyprych-Walczak J, Kunzmann UB, Crowell HL, Calini D, Malhotra D, Soneson C, Robinson MD. CellMixS: quantifying and visualizing batch effects in single-cell RNA-seq data. Life Sci Alliance. 2021;4**:**e202001004.

14. Korsunsky I, Millard N, Fan J, Slowikowski K, Zhang F, Wei K, Baglaenko Y, Brenner M, Loh PR, Raychaudhuri S. Fast, sensitive and accurate integration of single-cell data with Harmony. Nat Methods. 2019;16**:**1289-96.

15. Stuart T, Butler A, Hoffman P, Hafemeister C, Papalexi E, Mauck WM, 3rd, Hao Y, Stoeckius M, Smibert P, Satija R. Comprehensive integration of single-cell data. Cell. 2019;177**:**1888-902.

16. Chazarra-Gil R, van Dongen S, Kiselev VY, Hemberg M. Flexible comparison of batch correction methods for single-cell RNA-seq using BatchBench. Nucleic Acids Res. 2021;49**:**e42.

17. Buttner M, Miao Z, Wolf FA, Teichmann SA, Theis FJ. A test metric for assessing single-cell RNA-seq batch correction. Nat Methods. 2019;16**:**43-49.

18. Sanchis-Segura C, Ibanez-Gual MV, Aguirre N, Cruz-Gomez AJ, Forn C. Effects of different intracranial volume correction methods on univariate sex differences in grey matter volume and multivariate sex prediction. Sci Rep. 2020;10**:**12953.
