# Supplementary material for: Systematic evaluation with practical guidelines for single-cell and spatially resolved transcriptomics data simulation under multiple scenarios
Source: Genome Biol. 2024 Jun 3;25:145. doi: 10.1186/s13059-024-03290-y (PMC11149245; doi:10.1186/s13059-024-03290-y)
Supplement: Supplementary file 3 — Additional file 3. Review history. [file 13059_2024_3290_MOESM3_ESM.docx]

**1^st^ round**

**Reviewer 1**

- Are the methods appropriate to the aims of the study, are they well described, and are necessary controls included? If not, please specify what is required.
The authors include 49 simulation methods for single-cell and spatial transcriptomics data simulation data generation in terms of accuracy, functionality, scalability and usability. These methods, including the evaluation metrics and protocoal are well stated.

- Are the conclusions adequately supported by the data shown? If not, please explain
Most the conclusions are supported by the experiments, but the technical novelty or the indepth analysis are too superfacial and not so logical organized.

- Are sufficient details provided to allow replication and comparison with related analyses that may have been performed? If not, please specify what is required.
The authors shared their code and online tools for reproduction the experiments.
- Does the work represent a significant advance over previously published studies?
Not so solid. In fact, there are many related methods also do benchmark data imputation/ simulation of scRNA-seq and spatial data, the authors do not credit them in the right place and just state their work do not testify on so large datasets or a larger landscape.
I would like the authors to consider the more distinctive points of this benchmarking work and well organize the recent works on the similar targets.

- Is the paper of broad interest to others in the field, or of outstanding interest to a broad audience of biologists?
Yes, the authors indeed do a long team work to benchmark existing scRNA-seq data simulation methods, and used a largest number of datasets. The authors also introduced some evaluation metrics to compare existing methods for better comprehensiveness to choose the suitable tools.

Beside the answers to the above formal questions, I have some comments for the authors:
1. First of all, I credit that it it labor-intensive to do such a benchmark work with so many methods and datasets. There are also some similar benchmark works (i.e. [29] Cao, Yang, Yang. Nature Communication 2021. The more advanced for multi-omics data [21] scDesign3, Nature Biotechnology, 2023.), the authors are encouraged to clarify the difference and necessity of this work.
2. My main concern is the result discussion section, the authors mainly state out which method performs the best, which is the worst. But the authors do not discuss from the methodology and data characteristics. This overlook significantly blocks readers to catch the underlying reason and choose the suitable method in a rational or self-knowledgable way.
3. The method section is too long and not concise the present the key idea. How about more logically organizing them in a category manner, not just state out without solid background logic.
4. As to the evaluation metrics, they are so many and so tedious, which often confuse the readers. The organzation of them is also quite welcomed.
5. Page 14, Line 7, Methods such as SRTsim and scDesign2 addresses.... In fact, the deep models are mostly data distribution free can be adapted to different statistical models.
6. The author classified the data simulation methods into 5 categories, but it is hard to understand the underlying rule.
7. There are also many unclear or conflicting statements.
(1) Page 4 Line 9, machine learnign [28] have been proposed. In fact, it is quite controversial to say so. As many machine learning methods mostly define the statistics of the data.
(2) Page 4 Line 12, All these suggest that there are continous innovations along with advances in biological theories and machine learning. This problem statement or conclusion are quite simple and not so informative. Given there are already some simulation data benckmarking studies, I would like the authors to more throughtly organize and discuss them, and thus place their own work in a more suitable way.
(3) Page 16, Line 38 'failed estimation for genes' is not defined before.
(4) Page 16, Line 47, 'Our finding foffered crucial insights for developers on modelling gene expression data and understanding its characteristics and nature'. This statement is too strong and exaggerated.

**Reviewer 2**

In the present manuscript, the authors systematically evaluated 49 simulation methods for single-cell and spatial transcriptome data in terms of accuracy, functionality, scalability, and usability
using 152 reference datasets derived from 24 platforms. The benchmarking is rather comprehensive and clearly displayed. It's an important study and deserves to be considered for publication. I have the following concerns, mainly regarding the evaluation metrics.
Major comments:
1. Figure2: the difference of overall scores between different methods are not significant. It seems that this phenomenon was due to that the usability scores for most methods are similar. Is it possible to re-define and calculate the usability scores to account for their variations? The same issue may be also considered for the scalability score. In this way, the resulting overall scores may be clearly distinguished for most methods, which would benefit the ranking of the methods.
2. Figure 4: for most methods, the accuracy scores on the datasets generated by scRNA-seq and ST technology are not significantly distinguished. However, it may be due to the fact that the authors used the same metrics for evaluating the simulations of scRNA-seq data and ST data. In the current evaluations, the spatial information in the ST data was not considered in the metrics. So from my point of view, it is not surprise that "some methods tailored for single-cell transcriptome data have compatibility for simulating spatial data" as mentioned by the authors in the Abstract. Could the authors consider to employ additional metrics that account for the "spatial level", in addition to the "gene level", and "cell level" to re-evaluate the ST simulation?
3. The inclusion of evaluation of ST simulation methods is an advantage of this benchmarking study. So, could the authors include spatial specific functionalities (e.g., spatial variable genes, spatial domains or spatial clustering), for assessment in the "Functionality evaluation"? In addition, in Figure 2a, class of methods that can simulate ST data (e.g., scMultiSim, SRTsim, scDesign3, and more others) should be clearly labeled.
4. For the trajectory functionality evaluation, 13 spatial transcriptome datasets from 6 cancer types were involved, and the authors mentioned that the process of cancer invasion and metastasis can be regarded as a linear trajectory model. However, no results of the trajectories in space are displayed. What are the trajectories of these ST data in space? Do these trajectories of cancer invasion and metastasis follow the linear trajectory model that you have mentioned in the 'Spatial transcriptome datasets' section (Additional file 1; page 30)?
Minor comments:
1. Data simulation algorithms use different distribution models to fit distinct data properties and sample gene expression values from different distributions. For example, Splat uses the hierarchical model to fit different data properties (e.g. mean values of gene expression and library size of cells). Splat samples the count values from a gamma distribution while the negative binomial (NB) distribution is used in muscat. In the section of "Accuracy performance of methods with different models" in page 7 of MS, which part of the algorithms do the 'models' mentioned in the text have been summarized from? Also, do they represent the statistical distributions that were used for sampling gene expression values or fit certain data properties to estimate the parameters?
2. Lines 3-6 of page 6: I did not understand the exact meaning and what you did from your description of the 'usability assessment' and it should be illustrated elaborately in the main text.
3. Line 52 of page 6: 'e.g.' is required before the examples of metric scores ('MAD: 0.59±0.06 and OV: 0.64±0.12') as other metric results can also prove the conclusion.
4. Lines 9, 15 and 29 of page 7,: These abbreviations (e.g. CCC) were mentioned without full names which is hard to understand for readers. Even if they were illustrated in the Method section, I suggested that the full names are needed in the main text.
5. Lines 23-24 of page 7: the sentence of 'the gene-level properties are commonly used for modeling'. The word 'modeling' can not explicitly illustrate the meaning of the 'gene-level properties' and readers may get confused by the inaccurate description. Does it mean that the gene-level properties such as mean expression values of genes are usually used to fit statistical models in parameter estimation before data simulation?
6. Lines 12-21 of page 11: The models constructed by RF and SCAM can accurately predict the execution time and memory usage when the cell and gene numbers are given for every method. I want to know if these models are available for users to predict the potential occupied computational resources before they attempt to use some simulation methods.
7. The subsection of 'Real reference data' of Methods section (in page 17): Which form of the gene expression datasets did you include? Raw count or other normalized forms (such as CPM or TPM)? This should be noted in the 'Real reference data' section.
8. Line 33 of page 19 (in Methods section): To fully use the prior information associated with real reference data, authors extract and prepare the input information such as proportions of cell groups to custom the corresponding parameters in simulation methods. However, I was a bit unclear about what you mentioned the 'additional input information' in the sentence. Is it the input information that you have summarized from real data before data simulation, and you clearly illustrated in Supplementary Note Section 4.2?
9. The subsection of 'Functionality evaluation' in Methods section (in lines 26-44 of page 24): The authors described the research content and extra notes of the functionality evaluation, but some sentences were repeated when compared to those in the 'Parameter estimation and data simulation' section. I would suggest that the sentence from 'Before the simulation…' to 'are illustrated in Additional file 1: Supplementary Note Section 4.2' should be revised and moved to the 'Parameter estimation and data simulation' section in Methods.
10. It is wonderful that authors created and provided an online tool for method selection and data simulation for users. However, after testing I found that this web-based tool would report errors/bugs ("Error : cannot allocate vector of size 18.6 Gb") when dealing with large dataset simulation task (such as scRNA-seq datasets with 50000 cells by 50000 genes). So, can you provide the local version of this tool and make the code public for users? It might be good for users to run simulations on their computers and find out the bugs when they encounter errors.

**Authors’ response**

Reviewer 1

**[Question 1]** Are the methods appropriate to the aims of the study, are they well described, and are necessary controls included? If not, please specify what is required.

**[Reviewer]** The authors include 49 simulation methods for single-cell and spatial transcriptomics data simulation data generation in terms of accuracy, functionality, scalability and usability. These methods, including the evaluation metrics and protocoal are well stated.

**[Authors’ Response]** Thanks for your positive feedback.

**[Question 2]** Are the conclusions adequately supported by the data shown? If not, please explain

**[Reviewer]** Most the conclusions are supported by the experiments, but the technical novelty or the indepth analysis are too superfacial and not so logical organized.

**[Authors’ Response]** Thanks for your valuable suggestions. In conjunction with the discussion and review of previous studies (Cao et al. [1] and Crowell H L et al. [2]), we outline the importance and necessity of our work (**lines 72-105** in the revised manuscript**)**. Meanwhile, we extensively explore the key findings of our study and provide a detailed discussion of the methods’ accuracy performance and mathematical models behind them from the perspective of the methodology and characteristics of scRNA-seq and SRT data. According to the reviewers’ comments, we have reorganized the content of the *Discussion* section of the revised manuscript **(lines 325-442)**.

**[Question 3]** Are sufficient details provided to allow replication and comparison with related analyses that may have been performed? If not, please specify what is required.

**[Reviewer]** The authors shared their code and online tools for reproduction the experiments.

**[Authors’ Response]** Thanks for your positive feedback.

**[Question 4]** Does the work represent a significant advance over previously published studies?

**[Reviewer]** Not so solid. In fact, there are many related methods also do benchmark data imputation/ simulation of scRNA-seq and spatial data, the authors do not credit them in the right place and just state their work do not testify on so large datasets or a larger landscape. I would like the authors to consider the more distinctive points of this benchmarking work and well organize the recent works on the similar targets.

**[Authors’ Response]** Thanks for your comment and suggestion. We compared the two similar benchmark studies by Cao Y et al. (Nature Communications, 2021) [1] and Crowell H L et al. (Genome Biology, 2023) [2] with our study and illustrated the limitations of their studies that need to be addressed in the revised manuscript.

Briefly, the distinctive points of our benchmark work are as follows:

1. We summarized four essential method functionalities and fully assessed the performance of methods under multiple simulation scenarios.

2. From different perspectives, we adopted various metrics and proposed some novel approaches to elaborately quantify the reliability and fidelity of simulated gene expression data and different types of ground truth.

3. We systematically evaluated the methods developed for simulating SRT data in terms of accuracy, functionality, scalability and usability, which has not been conducted by other studies focusing on method benchmarks.

4. Interestingly, by using ‘spatial-level’ metrics, we found the well-performed simulation methods developed for scRNA-seq data can also generate reliable SRT datasets. We also discussed the underlying reasons for this phenomenon from the characteristics of scRNA-seq and SRT data.

5. Based on the evaluation results, we provided an explicit guideline for users, which gives context-specific recommendations for method selection. Additionally, we also established a standard pipeline of data simulation and result assessment to streamline the process within an R package and Docker image, making it more convenient for users in practical applications.

6. We carefully summarized ten types of execution errors during the work and provided practical suggestions on each of them, which will help developers avoid these common problems and improve the usability of methods.

Other points of our benchmark work are as follows:

1. We constructed the models to predict the execution time and memory usage for each method, which is useful for users to better understand the resource consumption when they are choosing simulation methods.

2. Lastly, we performed a comprehensive and systematic evaluation of 49 simulation methods by including a large number of real-world datasets, which can support users to gain a deep and well-rounded understanding of the method performance, and guide developers toward proposing more scalable and efficient methods.

**[Question 5]** Is the paper of broad interest to others in the field, or of outstanding interest to a broad audience of biologists?

**[Reviewer]** Yes, the authors indeed do a long team work to benchmark existing scRNA-seq data simulation methods, and used a largest number of datasets. The authors also introduced some evaluation metrics to compare existing methods for better comprehensiveness to choose the suitable tools.

**[Authors’ Response]** Thanks for your positive feedback.

**[Comment 1]** First of all, I credit that it it labor-intensive to do such a benchmark work with so many methods and datasets. There are also some similar benchmark works (i.e. [29] Cao, Yang, Yang. Nature Communication 2021. The more advanced for multi-omics data [21] scDesign3, Nature Biotechnology, 2023.), the authors are encouraged to clarify the difference and necessity of this work.

**[Response to Comment 1]** Thanks for your valuable and constructive suggestion. We carefully reviewed the differences between our work and the other two similar studies conducted by Cao Y et al. (Nature Communications, 2021) [1] and Crowell H L et al. (Genome Biology, 2023) [2], and we also summarized the main differences in the table below.

| Aspect | Item/Description | Cao, et al.  (2021, NC) | Crowell, et al.  (2023, GB) | Our study |
| --- | --- | --- | --- | --- |
| **Functionality evaluation** | Assessment of simulated cell groups | 🗶 | ✔ | ✔ |
|  | Assessment of simulated DEGs | ✔ | 🗶 | ✔ |
|  | Assessment of simulated cell batches | 🗶 | ✔ | ✔ |
|  | Assessment of simulated trajectories | 🗶 | 🗶 | ✔ |
| **Evaluation of SRT data simulators** | Evaluation of simulation methods for SRT data | 🗶 | 🗶 | ✔ |
|  | Assessment of simulated spatial domains | 🗶 | 🗶 | ✔ |
| **Practical application** | Pipeline for data simulation and result assessment | 🗶 | 🗶 | ✔ |
|  | Guideline for method selection | 🗶 | 🗶 | ✔ |
| **Usability evaluation** | Summary and analysis of execution errors | 🗶 | 🗶 | ✔ |
| Scalability evaluation | Exploration of time and memory complexity with respect to cell or gene numbers | 🗶 | 🗶 | ✔ |
|  | Useful models for predicting execution time and memory usage | 🗶 | 🗶 | ✔ |
| Research scale | Simulation methods | 12 | 16 | **49** |
|  | Reference datasets | 35 | 12 | **152** |
|  | Involved technical platforms | 12 | 16 | **23** |

**1. *Functionality evaluation.*** The other two benchmarks only considered partial simulation scenarios where methods could be applied. For example, the work conducted by Cao et al. only assessed the methods’ ability to simulate genes with different expression patterns. The study by Crowell et al. evaluated the reliability of simulated datasets with cell groups and batches. However, many current simulation methods, simultaneously possess the three functionalities, which have not been fully compared and evaluated. In addition, the methods developed for simulating the pre-defined trajectory were not included in these benchmark studies and their performance was still unclear. In this work, we fully evaluated the performance of methods under four simulation scenarios, including the generation of simulated cell groups (or spatial domains), DEGs (or spatially variable genes), cell batches and trajectories, which comprehensively quantified and demonstrated the applicable capability of simulators for dealing with different tasks.

Furthermore, the two benchmarks only adopted monotonous metrics for evaluating methods in each functionality. Taking the assessment of simulated DEGs for example, Cao et al. calculated the proportion difference of genes with distinct expression patterns between simulated and real data, to determine whether simulation methods can preserve the biological signals presented in the real data. However, this approach suffers from two limitations. Firstly, Cao et al. used the threshold of the gene fold change between cell groups to determine the type of expression pattern, which can be influenced by the default parameter settings of the fold change. As we know, the default fold changes of DEGs are quite different among most simulation methods. Secondly, Cao et al. did not verify the reliability of the simulated genes with different expression patterns. For example, whether the simulated DEGs between cell groups can improve the prediction ability of classification models, is of vital importance for the developers and users to believe the faithfulness of simulated DEGs in practical applications. In this benchmark, we assessed the reliability of simulated DEGs by additional two approaches: 1) determine whether the *P* values derived from the simulated data without DEGs agree with the assumption of uniform distribution, which reflects the reliability of simulated DEGs returned by simulators; 2) model the simulated expression data with only generated DEGs using Random Forest to quantify whether the simulated DEGs can be regarded as powerful features for training predictable models. In summary, we adopted many kinds of metrics to measure how faithful and reliable the simulated ground truth was from distinct perspectives.

**2. *Evaluation of SRT data simulators.*** The studies by Cao et al. and Crowell et al. did not involve methods for simulating spatial transcriptome data and did not evaluate their ability to generate reliable ground truth. In this study, we collected three newly developed methods (SRTsim [3], scDesign3 [4] and scMultiSim [5]) that can simulate SRT data and thoroughly assessed their functionality in simulating spatial domains and trajectories. Specifically, by employing simulators tailored to scRNA-seq data for simulating SRT data, we found that well-performed simulation methods for scRNA-seq data can also be compatible with generating reliable SRT data. That’s because the gene expression of SRT data shared similar features with that of scRNA-seq, such as over-dispersion and zero inflation [6] (see *Discussion* for detailed descriptions).

**3. *Practical application.*** We constructed an elaborate reference guideline for method selection, which gives context-specific recommendations for users. Meanwhile, we established a standard framework and pipeline for the process of data simulation and result assessment, which can be conveniently used to simulate datasets and evaluate newly developed methods. Nevertheless, the two similar studies did not pay much attention to these practical applications of data simulation for users, so it is not beneficial for them to choose and utilize suitable methods in a user-friendly manner.

**4. *Usability evaluation.*** The process of parameter estimation and data simulation often encounters execution errors for some methods, which may hinder their wide adoption in practical applications. Unfortunately, a thorough analysis of these execution errors has not been conducted by previous studies, and the underlying reasons for the execution failures are still unclear. In our study, we collected nearly 600 error items and summarized them into 10 categories. Approximate 61% of methods encountered errors, and the proportion of errors varied greatly across methods, ranging from 0.7% (SCRIP-BGP-trendedBCV and ZINB-WaVE) to 88.9% (BASiCS) (Fig. 3a,e). Importantly, we found there are two main sources of errors. First, the appearance of missing or infinite values in vectors or matrices can lead to errors when they are used in conditional statements or mathematical calculations. Second, in some methods, the gene expression values or gene-level properties cannot be successfully fitted, which is called the ‘Failed estimation for genes’ in the study. For example, ESCO cannot successfully model the relationship between log2 mean gene expression and the proportion of zeros. Based on the analysis of error sources, we provide developers with practical suggestions and refine the guidelines to prevent users from selecting methods with high error rates.

5. *Scalability evaluation.* In addition to monitoring execution time and memory usage, we explored the time and memory complexity with respect to cell or gene numbers for each method, which revealed that some methods (e.g., dyngen, dyntoy and scDesign3) are not suitable for performing parameter estimation and data simulation on large datasets. Furthermore, we also constructed models by random forest and a shape constrained additive model using scalability datasets, so that users can easily understand the estimated execution time and memory usage when they are choosing simulation methods.

6. *Research scale.* As the amount of scRNA-seq and SRT data grows rapidly, in this benchmark work, we collected 152 datasets (101 for scRNA-seq data and 51 for SRT data) generated by 24 technical platforms to avoid any potential bias introduced by the inadequate involved reference data. Additionally, we included 31 methods that were newly developed or have not been evaluated by Cao et al. and Crowell et al., because some methods may have advantages over others in terms of accuracy and functionality. Briefly, compared to previous works, we conducted a large-scale benchmark study of simulation methods for scRNA-seq and SRT data.

scDesign3 (Song et al., Nature Biotechnology, 2023) [4] is a multi-functional, flexible and interpretable statistical simulator to generate realistic single-cell and spatial omics data. This is a wonderful work as it provides a comprehensive solution for omics data simulation, accurately captures the data characteristics and allows convenient and transparent parameter tuning. However, this research mainly focused on the method development rather than benchmarks, so the evaluation work accounted for a small part of the research. As a result, Song et al. only included several related simulation methods (scGAN, muscat, SPARSim, SRTsim and ZINB-WaVE) and metrics (mLISI, Pearson correlation, KS distance and visualization approaches) to assess their accuracy and functionality performance.

In the revised manuscript **(lines 72-105)**, we elucidated the limitations of previous studies and then stressed the necessity of our work.

Given the diversities of underlying models implemented in the simulation methods and various applicable scenarios, it is necessary to comprehensively evaluate their performance. Efforts have been made toward the benchmarking of simulation methods for scRNA-seq data [31, 32], but they solely focused on partial simulation scenarios where methods could be applied, without fully assessing the fidelity and reliability of the simulated ground truth by multiple metrics from different viewpoints. Thus, this incomplete evaluation limits the broad adoption of methods with varied functionalities. For example, Cao et al. only assessed the ability of methods to simulate genes with different expression patterns, and the study by Crowell et al. merely considered the method functionalities of generating different cell batches and clusters. In particular, the simulation methods for SRT data have not been systematically evaluated by previous benchmark studies, so their abilities to generate realistic data and their performances under specific simulation scenarios (e.g., the simulation of spatial domains) are still unclear. Additionally, it is not beneficial for users to choose and use appropriate methods in a user-friendly way, without offering an explicit guideline for method selection and a standard pipeline from data simulation to result assessment. For some simulation methods, execution errors frequently arise during the process of parameter estimation and data simulation but the underlying reasons have not been summarized and analyzed. As a result, it is not conducive to improving current methods and advising users to adopt the methods with low error rates. In conclusion, addressing these limitations is imperative, as gaining a comprehensive understanding of the pros and cons of existing methods will empower users to make informed decisions. Moreover, it will assist developers in refining algorithms and provide new insights into the characteristics of scRNA-seq and SRT data

Here, we conducted a comprehensive assessment of 49 simulation methods for scRNA-seq and/or SRT data using 152 real reference datasets to evaluate their accuracy, functionality, scalability, and usability. Different from previous studies, we adopted various metrics and proposed several novel approaches to fully quantify the reliability of the simulated datasets and the functionality performance of methods under four simulation scenarios. Besides measuring the required time and occupied computational resources, we explored the time and memory complexity with respect to the cell or gene numbers for each simulation method. Additionally, we gathered up the error messages during the study and analyzed the underlying reasons for those execution failures. By elaborately summarizing the evaluation results, we provided an explicit guideline of method selection for users and a standard pipeline called Simpipe to achieve the process from data simulation to result assessment. Moreover, users can also access our interactive website (https://www.ciblab.net/software/simshiny/) to select suitable methods and simulate datasets. Our evaluation provides valuable insights for the development of new simulation algorithms and highlights the promising future of data simulation strategies for other prominent research fields, such as multi-omics and multi-modality.

**[Comment 2]** My main concern is the result discussion section, the authors mainly state out which method performs the best, which is the worst. But the authors do not discuss from the methodology and data characteristics. This overlook significantly blocks readers to catch the underlying reason and choose the suitable method in a rational or self-knowledgable way.

**[Response to Comment 2]** Your constructive suggestion is valuable for improving our manuscript. From the methodology and data characteristics you mentioned, in the reorganized *Discussion* section (**lines 325-442** in the revised manuscript) we deeply discussed the accuracy and functionality performance of methods. This thorough discussion aims to help readers understand the underlying rationale and make reasonable decisions.

In the discussion about the accuracy performance (**lines 325-422** in the revised manuscript):

a) We summarized three key factors that may influence the modeling of gene expression data and the effectiveness of data simulation for most methods: 1) the choice of prior distributional assumption, 2) the diversity of gene expression patterns, and 3) data variability.

b) Based on the three factors mentioned above, we discussed the accuracy performance of different models from their methodologies and the data characteristics. For example, SRTsim and scDesign2 are two methods based on the optimal-chosen model, which determines the most appropriate distribution for each gene from four candidate distributions (Poisson, ZIP, NB and ZINB). This strategy avoids the limitation of modeling gene expression data solely using a single parametric distribution, which can effectively characterize diverse gene expression patterns and can be applied across a broad spectrum of data. Especially, scDesign3, a multi-omics data simulator based on GAMLSS, has two outstanding advantages in characterizing data properties and gene expression heterogeneity from real data. scDesign3 allows the introduction of smooth functions and various covariates (e.g., experimental conditions, cell types, pseudotime and spatial coordinates) to model mean gene expression, dispersion and the proportion of zeros. It also models the joint distribution of multiple genes to preserve the correlation structure of genes, which can precisely delineate intricate relationships across different levels of gene expression in real datasets.

c) From the sparsity and variability nature of scRNA-seq, we explained in depth why some methods performed well and others poorly. For instance, ZINB-WaVE models the probability of dropouts in terms of cell- and gene-level covariates, which allows for the accurate characterization of the sparsity in scRNA-seq data. It also included a set of unobserved covariates to account for the unwanted variation (e.g., batch effect) and relevant biological effect of interest (e.g., cell type). In contrast, hierarchicell does not take into account the sparsity of scRNA-seq data as it removes genes with all zero values during data preprocessing. Although BASiCS explicitly characterizes unexplained technical variation or noise by modeling the observed values of 92 extrinsic molecules derived by the External RNA Controls Consortium (ERCC), it does not introduce parameters related to the zero values to account for data sparsity.

d) From the characteristics of SRT data, we discussed why some simulation methods developed for scRNA-seq data have powerful compatibility to simulate SRT data. Firstly, most SRT and scRNA-seq data share common features in terms of gene expression and data characteristics, such as excessive zeros and over-dispersion [6]. Secondly, gene expression values in most SRT data also follows the NB or Poisson distribution [6], which is similar to that of scRNA-seq data. In particular, over-dispersion and zero inflation in SRT data can be regarded as biologically important information [6], which is consistent with the interpretation and understanding of scRNA-seq data [7, 8]. Taken together, in some cases, developers can generally transfer the modeling strategy from scRNA-seq data to SRT data.

In the discussion about functionality performance (**lines 423-442** in the revised manuscript):

a) We elucidated why some methods (e.g., Splat, SCRIP, Lun and scMultiSim) can distort the simulated data and lead to over-simulation. That’s because they are excessively dependent on the customized parameters to simulate ground truth, without capturing heterogeneity or variance effects in real data by utilizing prior information (e.g., real labels of cell types and batches).

b) We explain why the performance of one functionality may affect that of another functionality for a specific simulation method. For instance, the gene expression levels of DEGs between cell groups may influence the reliability of the simulated cell groups, because whether cell groups can be clearly distinguished largely depends on the extent of differences in the expression levels of DEGs.

**[Comment 3]** The method section is too long and not concise the present the key idea. How about more logically organizing them in a category manner, not just state out without solid background logic.

**[Response to Comment 3]** Thank you for the valuable suggestion. As shown in the following outline, we reorganized this part in the *Methods* section **(lines 463-724)** of the revised manuscript to make it clear and logical.

**Methods**

Simulation methods

Real reference datasets

Parameter estimation and data simulation

Summary of data properties

Accuracy evaluation

Functionality evaluation

*Assessment of simulated cell groups/spatial domains*

*Assessment of simulated cell batches*

*Assessment of simulated DEGs*

*Assessment of simulated SVGs*

*Assessment of simulated trajectories*

Scalability evaluation

Usability evaluation

Score normalization and aggregation

Web-based vignettes, guidelines and data simulation tool

In addition, we deleted tedious descriptions and moved most illustrations of how the metrics were calculated to the Additional file 1. Taking the section of ‘*Assessment of simulated cell batches*’ as example (**lines 579-589** in the revised manuscript), the revised sentences are attached below:

***Assessment of simulated cell batches***

Seven metrics were used to measure the degree of batch effects in the simulated data, including cell-specific mixing score (CMS) [63], local inverse Simpson’s index (LISI) [64], mixing metric (MM) [65], Shannon entropy [66], kBET [67], average silhouette width for batch (ASW batch) and principal component regression score (PCR score) [67]. The five metrics, CMS, LISI, MM, Shannon entropy and kBET, determined the degree of batch-wise diversity within the 𝑘-nearest neighborhood (KNN) graphs, while the remaining two metrics reflected the reliability of batch populations and contribution of batch effects in the simulated data. The detailed information about these batch-specific metrics was illustrated in Additional file 1: Supplementary Note Section 5.3. It should be noted that the methods were expected to demonstrate powerful performance under this simulation scenario when the prominent batch effects were detected in the simulated data.

**[Comment 4]** As to the evaluation metrics, they are so many and so tedious, which often confuse the readers. The organzation of them is also quite welcomed.

**[Response to Comment 4]** Following your suggestion, we have moved most illustrations of the metric calculation process to Additional File 1. Descriptions of adopted metrics for accuracy and functionality assessments were also reorganized to improve readability (**lines 551-641** in the revised manuscript).

**[Comment 5]** Page 14, Line 7, Methods such as SRTsim and scDesign2 addresses.... In fact, the deep models are mostly data distribution free can be adapted to different statistical models.

**[Response to Comment 5]** We agree with your point that deep learning models are mostly data distribution-free. Thanks for the valuable suggestion. scGAN, a deep learning-based method without any distributional assumption, can be used for training various gene expression data. In this study, scGAN had mediocre performance on the accuracy and functionality of simulating cell groups. The reasonable explanation is that training scGAN on the library-size normalized scRNA-seq data can enhance its ability to generate high-quality simulated cells which was mentioned in the original paper [9]. However, the gene expression datasets used in our study only contained raw gene counts which were not normalized in the preprocessing step. We therefore thought that the usage of datasets with raw counts may affect the simulation performance of scGAN.

We rephrased the sentences about the performance of scGAN in the *Discussion* section (**lines 365-368** in the revised manuscript). For your convenience, we attached the sentences below:

While scGAN, a deep learning-based method, can discern different gene expression patterns, its performance on accuracy and functionality was found to be mediocre (Fig. 2,4b). This may stem from its sensitivity to the variance effects because generating high quality cells usually requires training scGAN on the library-size normalized data, as mentioned in the original paper [28].

**[Comment 6]** The author classified the data simulation methods into 5 categories, but it is hard to understand the underlying rule.

**[Response to Comment 6]** We are sorry for the unclear descriptions in the article of how the methods were categorized. Generally, the 49 simulation methods were classified into 5 categories through two steps. 1) First, we categorized the methods that can simulate trajectory data into a separate category (Class 1). This is because the requirement of simulating cell trajectories should be initially considered when users intend to apply the method functionality in the practical guideline (Fig. 7). 2) Next, the remaining methods were classified into 4 classes, based on the number of application scenarios where they can be applied. For example, Class5 methods can only simulate gene expression data, but cannot generate other types of ground truth (e.g., cell groups). Whereas Class2 methods can be used in three simulation scenarios, and generate datasets containing cell groups, DEGs and cell batches.

In conclusion, this classification allows readers to quickly know and understand which simulation scenarios each class of methods can be applied to and what functional characteristics they have. Especially when users need more than one functionality to get multiple types of ground truth, they can easily understand and select the appropriate method. As a last point, our evaluation results can be visualized in a pleasant and organized way (Fig. 2 and Fig. 3).

In the *Results* **(lines 112-116)** and *Methods* **(lines 466-478)** sections of the revised manuscript, we rephrased the corresponding parts. We attached them below for your convenience：

**Results**

**The overall benchmark framework**

A systematic and comprehensive evaluation of 49 simulation methods was performed to assess their accuracy, functionality, scalability, and usability, by using 101 single-cell and 51 spatial transcriptome datasets generated by 24 platforms, such as Smart-seq2 and Stereo-seq (Fig. 1a, Additional file 1: Fig. S1 and Additional file 2: Table S1-3). To clarify the simulation scenarios that current methods can be applied to, we summarized four essential aspects of functionality, including the abilities to simulate cell groups (or spatial domains), DEGs, cell batches and cell differentiation trajectories (Fig. 1b). All methods were categorized into 5 classes, according to the type and number of simulation scenarios they can be employed (Fig. 2a, Additional file 2: Table S1 and Methods). Moreover, we unified the parameter names across different functionalities and methods to simplify user-defined settings (Additional file 1: Supplementary Notes, Additional file 2: Table S2).

… …

**Methods**

**Simulation methods**

We collected 49 simulation methods designed for scRNA-seq and SRT data under the survey of literature (Additional file 2: Table S1). Among them, SRTsim, scDesign3, scDesign3-tree, scMultiSim and scMultiSim-tree were specifically developed for simulating SRT data. We classified the methods into 5 categories through two steps. First, the methods that were able to simulate trajectory data were gathered into a separate category, because the requirement of simulating cell trajectories should be initially considered when users intend to apply the method functionality based on the practical guideline (Fig. 7). Next, we classified the remaining methods into 4 classes, by the number of application scenarios where they can be applied. The classification of methods is shown below:

- Class1 methods can simulate data with cell differentiation trajectory;
- Class2 methods can be simultaneously applied to three simulation scenarios: simulation of cell groups (or spatial domains), DEGs and cell batches;
- Class3 methods are limited to concurrent use in only two simulation scenarios;
- Class4 methods have only one useful simulation functionality;
- Class5 methods can only simulate gene expression data without having other functionalities.

**[Comment 7]** There are also many unclear or conflicting statements.

Page 4 Line 9, machine learnign [28] have been proposed. In fact, it is quite controversial to say so. As many machine learning methods mostly define the statistics of the data.

**[Response to Comment 7]** Thanks for pointing out the inappropriate statement. We rewrote this sentence in the revised manuscript **(lines 60-66)**, which is also shown below:

**Background**

In recent years, a plethora of tools have been developed to simulate scRNA-seq data [6] and methods tailored to simulate SRT data have emerged in 2023 [20-22]. Most simulation methods were developed mainly based on statistical distributions, in which a reference dataset was used to estimate important parameters. The new gene expression values were then drawn from the sample space using those estimated parameters [23-26]. Those parametric methods with distributional assumptions are flexible and enable to handle simulation tasks under various scenarios, but the assumptions may not always hold true due to the biological and technical variability in the data [27]. Alternatively, a deep learning-based method was proposed to generate in silico scRNA-seq data [28], utilizing generative adversarial networks (GAN) to avoid pre-defined assumptions. To enhance the interpretability of these methods, researchers have shifted their focus towards simulating scRNA-seq data based on the principle of gene expression regulation [29] and RNA velocity theory [30]. Apart from the gene expression matrices, numerous methods can also be applied to four simulation scenarios for generating different types of ground truth, including disparate cell groups (or spatial domains), differentially expressed genes (DEGs), cell batches and trajectories. This versatility enables researchers to assess the performance of analytical methods across a spectrum of simulated biological contexts and enhance the robustness of experimental designs.

Simultaneously, we reviewed the entire text from start to finish and rectified all inappropriate descriptions. All changed texts have been marked in blue in the revised manuscript.

**[Comment 8]** Page 4 Line 12, All these suggest that there are continous innovations along with advances in biological theories and machine learning. This problem statement or conclusion are quite simple and not so informative. Given there are already some simulation data benckmarking studies, I would like the authors to more throughtly organize and discuss them, and thus place their own work in a more suitable way.

**[Response to Comment 8]** Thanks for your constructive comments and suggestions. In the revised manuscript, we have deleted the inappropriate statement ‘All these suggest that there are continuous innovations along with advances in biological theories and machine learning’.

According to the comment of **#[Comment 1]**, we fully discussed the limitations of two similar studies and clarified the necessity of our work in the *Background* section (**lines 72-105** in the revised manuscript).

**[Comment 9]** Page 16, Line 38 'failed estimation for genes' is not defined before.

**[Response to Comment 9]** As you said, we did leave out the definition of 'failed estimation for genes'. In the revised manuscript, we defined it in the section of ‘*Usability and execution errors*’ **(lines 307-311)**. It is also attached below:

**Results**

**Usability and execution errors**

We further summarized 10 categories of execution errors (Fig. 3e, Additional file 2: Table S5). Approximate 61% of methods encountered errors, and the error proportions varied greatly across methods, ranging from 0.7% (SCRIP-BGP-trendedBCV and ZINB-WaVE) to 88.9% (BASiCS) (Fig. 3a,e). The error type called “appearance of missing (infinite) values” accounted for a substantial part in many methods. This can be attributed to unexpected missing values in vectors or matrices, resulting in errors when they are utilized in conditional statements or mathematical calculations. Certain methods, such as BASiCS, hierarchicell and ESCO, frequently encountered failures in parameter estimation. It was the error termed “failed estimation for genes” that generally occurred in the situation where the expression values of some genes may not conform with the distribution or the gene-level properties cannot be successfully fitted by the model implemented in these methods. Drawing upon the main causes, we provided developers with practical recommendations to avoid common errors (Additional file 2: Table S6).

**[Comment 10]** Page 16, Line 47, 'Our finding foffered crucial insights for developers on modelling gene expression data and understanding its characteristics and nature'. This statement is too strong and exaggerated.

**[Response to Comment 10]** Thanks for pointing out the inappropriate statement. We rephrased this sentence (**lines 459-461** in the revised manuscript), which is shown below:

Our study will help users to navigate the space of prevalent simulation methods, guide developers toward proposing more scalable and efficient methods and promote their understanding of the characteristics of gene expression data.

Reviewer 2:

**[Overall comment]** In the present manuscript, the authors systematically evaluated 49 simulation methods for single-cell and spatial transcriptome data in terms of accuracy, functionality, scalability, and usability using 152 reference datasets derived from 24 platforms. The benchmarking is rather comprehensive and clearly displayed. It's an important study and deserves to be considered for publication. I have the following concerns, mainly regarding the evaluation metrics.

**[Response]** We really appreciate your positive feedback.

**[Comment 1]** Figure2: the difference of overall scores between different methods are not significant. It seems that this phenomenon was due to that the usability scores for most methods are similar. Is it possible to re-define and calculate the usability scores to account for their variations? The same issue may be also considered for the scalability score. In this way, the resulting overall scores may be clearly distinguished for most methods, which would benefit the ranking of the methods.

**[Response to Comment 1]** Following your valuable suggestions, we changed the approach for calculating usability and scalability scores. For scalability, the raw measurements were initially log2-transformed before being scaled into the standard normal distribution and then transformed into (0,1) by the cumulative distribution function (CDF). For usability, we multiplied the manual scores by the corresponding weights and then summed them up by each aspect of usability (i.e., method availability, code quality, self-evaluation, maintenance, documentation, and publication). Next, the values from the same aspect of usability were scaled to the standard normal distribution and were subsequently transformed into (0,1) by the CDF, which resulted in the scores of different aspects. The final usability score for each method was obtained (Fig. 2b) by calculating the arithmetic mean of values across the six aspects.

As a result, the differences of scalability, usability and overall scores among methods can be clearly distinguished (Fig. 2). The corresponding descriptions were also revised in *Methods* section (**lines 687-699** in the revised manuscript).

**Methods**

**Usability evaluation**

To obtain usability scores, firstly we allocated weights for the scoring items depending on the frequency they were mentioned in the nine references. We multiplied the manual scores by the corresponding weights to get the values and they were then summed up by each aspect of usability. Next, the values from the same aspect of usability were scaled to the standard normal distribution (μ=0, σ=1) and were subsequently transformed into (0,1) by the cumulative distribution function (CDF). The values of six aspects were visualized in Fig. 3e. The final usability score for each method was obtained (Fig. 2b) by calculating the arithmetic mean of values from the six aspects of usability.

**Score normalization and aggregation**

We summarized the final scores of accuracy, functionality and scalability by two stages of data processing. In the first stage, we normalized the scores on three levels: datasets, metrics and data properties, to transform them into the range from 0 to 1. In particular, the raw measurements of scalability were initially log2-transformed before being processed in downstream steps. As an example of calculating the accuracy scores (Additional file 1: Fig. S30), we first scaled the scores from the same dataset across different methods to the standard normal distribution (μ=0, σ=1). They were then transformed into (0,1) by the CDF. As the values closing to 1 usually indicate higher accuracy, the values of some metrics therefore need to be subtracted from 1 to keep them harmonized. Subsequently, across different methods and datasets, we scaled the scores from the same data property and metric into [0,1] by the following formula.

Fig. 2 is attached below:

**[Comment 2]** Figure 4: for most methods, the accuracy scores on the datasets generated by scRNA-seq and ST technology are not significantly distinguished. However, it may be due to the fact that the authors used the same metrics for evaluating the simulations of scRNA-seq data and ST data. In the current evaluations, the spatial information in the ST data was not considered in the metrics. So from my point of view, it is not surprise that "some methods tailored for single-cell transcriptome data have compatibility for simulating spatial data" as mentioned by the authors in the Abstract. Could the authors consider to employ additional metrics that account for the "spatial level", in addition to the "gene level", and "cell level" to re-evaluate the ST simulation?

**[Response to Comment** **2]** Thanks for your constructive suggestion. We adopted additional two metrics, ‘spatial-multiKS’ and ‘spatial-KDE’, to re-evaluate the capability of methods for simulating SRT datasets by considering the spatial coordinates of spots (X and Y) into account. In this way, we defined the spots with three-dimensional features, where two of them represent the spatial coordinates and another feature represents the data property summarized from the real and simulated SRT data. Since the improved Kolmogorov-Smirnov test and kernel density-based global comparison test can be applied to the high-dimensional data, we can test whether the high-dimensional features of the real and simulated spots come from the same underlying distribution. However, we encountered an inevitable challenge that, except for SRTsim and scDesign3, other methods do not provide spatial positions for the simulated SRT data. To solve this problem, we used the Hungarian algorithm to match the simulated spatial spots with those spots in the real SRT data. Thus, the spatial coordinates of spots in the real data can be transferred to the matched simulated spots.

According to the newly calculated metric scores, we found that the consistency still exists between the accuracy performances of methods on scRNA-seq and SRT datasets (Pearson’s *r*=0.83, Fig. 4c) (**lines 174-183** in the revised manuscript).

Additionally, we talked about the performance consistency from the characteristics of SRT data in the *Discussion* (**lines 409-422** in the revised manuscript). A study by Zhao et al. has proven that expression values of most genes in SRT data followed the negative binomial or Poisson distribution [6], which has been accepted by researchers for modeling scRNA-seq data. Therefore, this phenomenon can be interpreted and verified by the commonly shared features of scRNA-seq and SRT data, and we can conclude that some methods tailored for scRNA-seq data have the compatibility for simulating reliable SRT data.

The corresponding descriptions in *Methods* section were also revised (**lines 561-569** in the revised manuscript).

**Methods**

**Accuracy evaluation**

Specifically, we employed the variant versions of multiKS and KDE, termed ‘spatial-multiKS’ and ‘spatial-KDE’, to account for the ‘spatial level’ of accuracy for the simulated SRT data. They can quantify the similarity between the simulated and real SRT data by considering the spatial positions or coordinates (axes of X and Y) as two-dimensional features defined in both the real and simulated data. Unfortunately, except for SRTsim and scDesign3, other methods do not provide spatial positions for the simulated SRT data, which raised an inevitable challenge in calculating the metrics. To solve this problem, we adopted the Hungarian algorithm to match the simulated spatial spots with those spots in the real SRT data, so that the spatial coordinates of spots in real data can be transferred to the matched simulated spots.

**[Comment 3]** The inclusion of evaluation of ST simulation methods is an advantage of this benchmarking study. So, could the authors include spatial specific functionalities (e.g., spatial variable genes, spatial domains or spatial clustering), for assessment in the "Functionality evaluation"? In addition, in Figure 2a, class of methods that can simulate ST data (e.g., scMultiSim, SRTsim, scDesign3, and more others) should be clearly labeled.

**[Response to Comment 3]** Thanks for the valuable suggestion. We have revised the descriptions of the spatial functionality assessment. Furthermore, we adopted Moran’s I statistics to quantify the reliability of simulated SVGs.

First, we added the subsection about the spatial specific functionalities and terminologies tailored to SRT data in the section of ‘*Functionality evaluation*’. For the assessment of simulated spatial domains (**lines 572-577** in the revised manuscript), we adopted the six metrics, such as average silhouette width (ASW) and Davies-Bouldin index (DB index). Moreover, we inserted a subsection for describing the assessment of the simulated spatial variable genes (SVGs) (**lines 623-630** in the revised manuscript). The corresponding corrections are as follows:

***Assessment of simulated cell groups/spatial domains***

To quantity the reliability of simulated cell groups in scRNA-seq data or spatial domains in SRT data, we adopted six metrics that are commonly used to evaluate different aspects of clustering performance [58], including average silhouette width (ASW) [59], Dunn index, Connectivity [60], Davies-Bouldin index (DB index), clustering deviation index (CDI) [61] and ROUGE [62]. The detailed information of the metrics was described in Additional file 1: Supplementary Note Section 5.2.

… …

***Assessment of simulated SVGs***

For the simulated SRT data, the reliability of spatially variable genes (SVGs) was also assessed by the approaches used for the DEGs in simulated scRNA-seq data. Furthermore, we evaluated the simulated SVGs using Moran’s I statistics which are commonly used to quantify the degree of autocorrelation of gene expression in space [69-71]. The expression patterns of SVGs are supposed to exhibit high spatial autocorrelation because the expression values of spots have a strong relationship with those spots near them. The Moran’s I statistics range from -1 to 1, where a value close to 1 indicates a clear spatial pattern, close to 0 indicates the random gene expression and close to -1 indicates a chessboard-like pattern.

Second, we added another metric, Moran’s I statistics, to quantify the degree of gene expression autocorrelation of simulated SVGs. According to the results, we found that Moran’s I statistics were close to 0 (Additional file 1: Fig. S10) for all simulation methods, which indicated that there is no relationship between the gene expression levels within adjacent spots. Thus, simulation methods tailored to scRNA-seq data cannot generate genes with clear expression patterns in space.

The corresponding changes in the *Results* section (**lines 238-244** in the revised manuscript) are as follows:

**Method performance on different functionalities**

Specifically, we adopted Moran’s I statistics to quantify the degree of autocorrelation for the simulated spatially variable genes (SVGs) in mimicked SRT data. The results showed that Moran’s I statistics deviated far from 1 (Additional file 1: Fig. S10), indicating that there is no relationship between the gene expression levels within adjacent spots. Consequently, the simulation methods can hardly generate SRT data with clear expression patterns of SVGs in space. This limitation may arise from the incapacity of scRNA-seq data simulators to additionally integrate spatial coordinates and effectively capture the gene expression patterns across spatial domains from reference SRT data.

Lastly, to highlight the methods enabling the simulation of SRT data, we added another column of characteristic information showing whether the methods can be employed to generate SRT or omics data in **Figure 2a** (as shown in **[Response to Comment 1] for Reviewer #2**).

**[Comment 4]** For the trajectory functionality evaluation, 13 spatial transcriptome datasets from 6 cancer types were involved, and the authors mentioned that the process of cancer invasion and metastasis can be regarded as a linear trajectory model. However, no results of the trajectories in space are displayed. What are the trajectories of these ST data in space? Do these trajectories of cancer invasion and metastasis follow the linear trajectory model that you have mentioned in the 'Spatial transcriptome datasets' section (Additional file 1; page 30)?

**[Response to Comment 4]** We employed Slingshot [10] to perform trajectory inference analysis for 13 spatial transcriptome data (Additional file 1: Supplementary Note ‘*Datasets with cell differentiation trajectory*’) and then visualized the results (Additional file 1: Fig. S25). Except for the bifurcation structure inferred from data107, the other trajectories were all linear topologies. The result of bifurcation topology may be due to the more complex tumor microenvironment presented in the data. In these inference results, such as in data105, data119, data121 and data122, we observed the malignant cells invaded and infected the regions containing healthy cells, which verified the conclusion that the process of cancer invasion and metastasis can be considered as a linear trajectory model [11, 12]. In addition, we found that some results showed the transition process from healthy cells to invasive cancer cells in data102, data104, data107 and data120 and they also exhibited the linear trajectory in space.

**[Comment 5]** Data simulation algorithms use different distribution models to fit distinct data properties and sample gene expression values from different distributions. For example, Splat uses the hierarchical model to fit different data properties (e.g. mean values of gene expression and library size of cells). Splat samples the count values from a gamma distribution while the negative binomial (NB) distribution is used in muscat. In the section of "Accuracy performance of methods with different models" in page 7 of MS, which part of the algorithms do the 'models' mentioned in the text have been summarized from? Also, do they represent the statistical distributions that were used for sampling gene expression values or fit certain data properties to estimate the parameters?

**[Response to Comment 5]** The distributional models of most simulation methods are used for characterizing gene expression data by fitting necessary data properties to estimate the parameters. For instance, muscat uses a negative binomial (NB) distribution to estimate the parameters from a reference dataset and generates new gene counts from the established model. In addition, the core models of some methods may also represent the statistical distribution where the gene counts are sampled. For example, Splat adopts the gamma-poisson hierarchical model, which was mentioned in the paper. In this model, mean gene expression was fitted by a gamma distribution, and gene counts were sampled from a Poisson distribution with modifications to account for the outliers and mean-variance trend.

**[Comment 6]** Lines 3-6 of page 6: I did not understand the exact meaning and what you did from your description of the 'usability assessment' and it should be illustrated elaborately in the main text.

**[Response to Comment 6]** Thanks for pointing out this ambiguous description. In the revised manuscript **(lines 125-128)**, we have clearly described how and what we did in the usability assessment. For your convenience, the updated sentences are attached below:

Except for the methods lacking functionality (assessed by three criteria), the remains were assessed using four criteria (Fig 1b, Methods): (1) Accuracy was assessed by 8 metrics based on 15 data properties on the cell or gene level, reflecting the ability to generate ‘realistic’ simulated data. (2) Functionality evaluation was an unbiased quantification of method performance in four simulation scenarios with different metrics. (3) Scalability evaluation was performed to monitor the occupied computational resources and unveil the relationship between execution time or memory usage and the number of cells or genes. (4) Usability assessment was conducted by manually scoring the terms of the checklist which was summarized from nine authoritative references [33-41]. Usability covered six aspects: method accessibility, code quality, self-evaluation, maintenance, documentation and publication (Additional file 2: Table S4).

**[Comment 7]** Line 52 of page 6: 'e.g.' is required before the examples of metric scores ('MAD: 0.59±0.06 and OV: 0.64±0.12') as other metric results can also prove the conclusion.

**[Response to Comment 7]** Thanks for pointing out this mistake. The corrected statement (**lines 145-146** in the revised manuscript) is shown below:

SRTsim, ZINB-WaVE, scDesign3-tree, scDesign3, SPARSim, muscat and scDesign2 showed superior performance on all accuracy metrics (Fig. 3a,b), while some methods such as phenopath, Lun2 and hierarchicell typically yielded low scores. Additionally, for Class2 methods, the scores of most metrics (e.g., KS: 0.680.11, MAD: 0.590.06 and OV: 0.640.12) were higher and more robust than those of other classes (Additional file 1: Fig. S2a), suggesting that they possess the powerful ability to simulate data which resembled the real data.

**[Comment 8]** Lines 9, 15 and 29 of page 7,: These abbreviations (e.g. CCC) were mentioned without full names which is hard to understand for readers. Even if they were illustrated in the Method section, I suggested that the full names are needed in the main text.

**[Response to Comment** **8]** Thanks for the valuable suggestion. We added the full names of the data properties mentioned in the revised manuscript with their abbreviations **(lines 149-164)**. The revised paragraph is attached below:

Regarding the scores given to each data property, some methods constantly achieved good performance on the characterization of different properties from real data (e.g., SRTsim, scDesign3 and ZINB-WaVE), while some of the others (e.g. hierarchicell, MFA, scMultiSim and CancerInSilico) demonstrated poorly to simulate data that resembled the real one (Additional file 1: Fig. S3). We also found that some data properties, such as the cell-cell correlation (CCC) and fraction of cell outliers (FCO), can only be captured well by certain methods. For instance, the FCO property was well characterized by SPARsim (score=0.89) and SRTsim (score=0.86). SRTsim also showed an obviously good performance in CCC (score=0.90). Remarkably, the methods showing significant differences in accuracy scores between gene-level and cell-level properties performed excellently in the former but faltered in the latter (Additional file 1: Fig. S4). That’s probably because current methods mainly focus on the modeling of various gene-level properties, such as mean and standard deviation of gene expression, gene-wise dropout and gene-gene correlations, but only a few of cell-level properties (e.g., cell library size and cell-wise dropout) were characterized from real data. For the Class2 methods, they still showed appreciable performance across many data properties (Additional file 1: Fig. S2b), such as library size per cell (LS: 0.70±0.11), effective library size per cell (ELS: 0.69±0.11), and fraction of zeros per cell (FZC: 0.60±0.10), as their scores were more robust and higher than those of other classes.

**[Comment 9]** Lines 23-24 of page 7: the sentence of 'the gene-level properties are commonly used for modeling'. The word 'modeling' can not explicitly illustrate the meaning of the 'gene-level properties' and readers may get confused by the inaccurate description. Does it mean that the gene-level properties such as mean expression values of genes are usually used to fit statistical models in parameter estimation before data simulation?

**[Response to Comment 9]** We are sorry for the ambiguous statement. Actually, besides gene-level properties such as the mean and standard deviation of gene expression values, cell-level properties (e.g. cell library size) are also essential for methods to capture the data characteristics. However, current methods mainly focus on the modeling of various gene-level properties, such as mean and standard deviation of gene expression, gene-wise dropout and gene-gene correlation, but only characterize a few of cell-level properties (e.g. cell library size). In this context, many gene-level properties can be explicitly captured from real data, which may lead to higher similarity of data properties on the gene level than the cell level. We have revised this imprecise sentence in the revised manuscript **(lines 156-161)** which is attached below for your convenience:

Remarkably, the methods showing significant differences in accuracy scores between gene-level and cell-level properties performed excellently in the former but faltered in the latter (Additional file 1: Fig. S4). That’s probably because current methods mainly focus on the modeling of various gene-level properties, such as mean and standard deviation of gene expression, gene-wise dropout and gene-gene correlations, but only a few of cell-level properties (e.g., cell library size and cell-wise dropout) were characterized from real data.

**[Comment 10]** Lines 12-21 of page 11: The models constructed by RF and SCAM can accurately predict the execution time and memory usage when the cell and gene numbers are given for every method. I want to know if these models are available for users to predict the potential occupied computational resources before they attempt to use some simulation methods.

**[Response to Comment 10]** The well-established models are available to the public and can be accessed in the *data* folder of the *simshiny* R package (https://github.com/duohongrui/simshiny). If users do not care about the exact result of the predicted execution time and memory usage, they can follow the online guidance on method selection to choose suitable simulators based on their requirements via https://www.ciblab.net/software/simshiny/.

**[Comment 11]** The subsection of 'Real reference data' of Methods section (in page 17): Which form of the gene expression datasets did you include? Raw count or other normalized forms (such as CPM or TPM)? This should be noted in the 'Real reference data' section.

**[Response to Comment 11]** Thanks for the valuable suggestion. In the research, we only included gene expression datasets with raw counts and did not normalize them into CPM or TPM. In the revised manuscript **(lines 487-488)**, we corrected the descriptions of the data form we used. For your convenience, the updated sentences are attached below:

In total, 152 real reference datasets (101 scRNA-seq and 51 SRT datasets) with raw counts were collected from 13 scRNA-seq platforms and 11 technologies for spatial transcriptomics.

**[Comment 12]** Line 33 of page 19 (in Methods section): To fully use the prior information associated with real reference data, authors extract and prepare the input information such as proportions of cell groups to custom the corresponding parameters in simulation methods. However, I was a bit unclear about what you mentioned the 'additional input information' in the sentence. Is it the input information that you have summarized from real data before data simulation, and you clearly illustrated in Supplementary Note Section 4.2?

**[Response to Comment 12]** We are sorry for the imprecise statement. Indeed, the ‘additional input information’ refers to the input information (e.g., the number of cell groups and the proportion of DEGs) that we have summarized from real data before data simulation. In the revised manuscript **(lines 511-520)**, we corrected this sentence for clarity which is shown below:

In the data simulation step, an important problem is how to fully use the information of the reference datasets (such as the number of cell groups and the proportion of DEGs) to conduct an objective and unbiased evaluation. Before the simulation, we extracted and prepared the input information related to cell groups, DEGs and batches from the reference datasets, to ensure the consistency of some cell- or gene-level information between the simulated and real data in this study. For example, if the real data contains two cell batches and three cell groups, we will simulate datasets with the same number of cell batches and groups. Similarly, data consistency is ensured by setting the proportion of DEGs between cell groups. Specifically, the input information is optionally used depending on the parameters that the methods contain. Detailed descriptions of extracting information and setting parameters before the simulation are illustrated in Additional file 1: Supplementary Note Section 4.2.

**[Comment 13]** The subsection of 'Functionality evaluation' in Methods section (in lines 26-44 of page 24): The authors described the research content and extra notes of the functionality evaluation, but some sentences were repeated when compared to those in the 'Parameter estimation and data simulation' section. I would suggest that the sentence from 'Before the simulation…' to 'are illustrated in Additional file 1: Supplementary Note Section 4.2' should be revised and moved to the 'Parameter estimation and data simulation' section in Methods.

**[Response to Comment 13]** Thank you for the valuable suggestion. In the revised manuscript, we moved the sentences (from ‘Before the simulation…’ to ‘are illustrated in Additional file 1: Supplementary Note Section 4.2’) to the ‘*Parameter estimation and data simulation*’ section in *Methods* (**lines 511-520** in the revised manuscript). We rewrote this paragraph for precise description and logical coherence:

In the data simulation step, an important problem is how to fully use the information of the reference datasets (such as the number of cell groups and the proportion of DEGs) to conduct an objective and unbiased evaluation. Before the simulation, we extracted and prepared the input information related to cell groups, DEGs and batches from the reference datasets, to ensure the consistency of some cell- or gene-level information between the simulated and real data in this study. For example, if the real data contains two cell batches and three cell groups, we will simulate datasets with the same number of cell batches and groups. Similarly, data consistency is ensured by setting the proportion of DEGs between cell groups. Specifically, the input information is optionally used depending on the parameters that the methods contain. Detailed descriptions of extracting information and setting parameters before the simulation are illustrated in Additional file 1: Supplementary Note Section 4.2.

**[Comment 14]** It is wonderful that authors created and provided an online tool for method selection and data simulation for users. However, after testing I found that this web-based tool would report errors/bugs ("Error : cannot allocate vector of size 18.6 Gb") when dealing with large dataset simulation task (such as scRNA-seq datasets with 50000 cells by 50000 genes). So, can you provide the local version of this tool and make the code public for users? It might be good for users to run simulations on their computers and find out the bugs when they encounter errors.

**[Response to Comment 14]** Thank you for the positive feedback. We provided the free-available *simshiny* R package in our Github repository (https://github.com/duohongrui/simshiny) which can be downloaded and installed as the local version of the online tool for users. The corresponding sentence was updated in the section of ‘*Web-based vignettes, guidelines and data simulation tool*’ (**lines 723-724** in the revised manuscript).

We established a website (https://www.ciblab.net/software/Simsite/) to illustrate the usage of our simulation pipeline (https://github.com/duohongrui/simpipe) by using rmarkdown (v2.23) and blogdown (v1.18) R packages. The online guideline of method selection and data simulation tool was built using golem (v0.4.1), shiny (v1.7.4.1) and shinythemes (v1.2.0) R packages, which can be accessed at https://www.ciblab.net/software/simshiny/. The local version of the online tool can also be installed in R from Github (https://github.com/duohongrui/simshiny).

We sincerely appreciate the time and effort invested by the editors and reviewers in evaluating our manuscript. We believe that these revisions will significantly strengthen our manuscript and provide a more robust foundation for our claims.

**References**

1.	Cao Y, Yang P, Yang JYH. A benchmark study of simulation methods for single-cell RNA sequencing data**.** Nat Commun. 2021;12**:**6911.

2.	Crowell HL, Morillo Leonardo SX, Soneson C, Robinson MD. The shaky foundations of simulating single-cell RNA sequencing data**.** Genome Biol. 2023;24**:**62.

3.	Zhu J, Shang L, Zhou X. SRTsim: spatial pattern preserving simulations for spatially resolved transcriptomics**.** Genome Biol. 2023;24**:**39.

4.	Song D, Wang Q, Yan G, Liu T, Sun T, Li JJ. scDesign3 generates realistic in silico data for multimodal single-cell and spatial omics**.** Nat Biotechnol. 2024;42**:**247-52.

5.	Li H, Zhang Z, Squires M, Chen X, Zhang X. scMultiSim: simulation of single cell multi-omics and spatial data guided by gene regulatory networks and cell-cell interactions**.** Res Sq. 2023.

6.	Zhao P, Zhu J, Ma Y, Zhou X. Modeling zero inflation is not necessary for spatial transcriptomics**.** Genome Biol. 2022;23**:**118.

7.	Svensson V. Droplet scRNA-seq is not zero-inflated**.** Nat Biotechnol. 2020;38**:**147-50.

8.	Choi K, Chen Y, Skelly DA, Churchill GA. Bayesian model selection reveals biological origins of zero inflation in single-cell transcriptomics**.** Genome Biol. 2020;21**:**183.

9.	Marouf M, Machart P, Bansal V, Kilian C, Magruder DS, Krebs CF, Bonn S. Realistic in silico generation and augmentation of single-cell RNA-seq data using generative adversarial networks**.** Nat Commun. 2020;11**:**166.

10.	Street K, Risso D, Fletcher RB, Das D, Ngai J, Yosef N, Purdom E, Dudoit S. Slingshot: cell lineage and pseudotime inference for single-cell transcriptomics**.** BMC Genomics. 2018;19**:**477.

11.	Ren Y, Huang Z, Zhou L, Xiao P, Song J, He P, Xie C, Zhou R, Li M, Dong X, et al. Spatial transcriptomics reveals niche-specific enrichment and vulnerabilities of radial glial stem-like cells in malignant gliomas**.** Nat Commun. 2023;14**:**1028.

12.	Shang L, Zhou X. Spatially aware dimension reduction for spatial transcriptomics**.** Nat Commun. 2022;13**:**7203.

**2^nd^ round**

**Reviewer 1**

This paper gives a good benchmark and pipeline for for single-cell and spatially resolved transcriptomics data simulation. The authors clearly addressed my comments and greatly expanded the insights for choosing the suitable methods for specific usages

**Reviewer 2**

The authors have addressed my comments
